# ANTIAGE-DB: A Database and Server for the Prediction of Anti-Aging Compounds Targeting Elastase, Hyaluronidase, and Tyrosinase

**DOI:** 10.3390/antiox11112268

**Published:** 2022-11-17

**Authors:** Christina D. Papaemmanouil, Jorge Peña-García, Antonio Jesús Banegas-Luna, Androniki D. Kostagianni, Ioannis P. Gerothanassis, Horacio Pérez-Sánchez, Andreas G. Tzakos

**Affiliations:** 1 Department of Chemistry, Section of Organic Chemistry and Biochemistry, University of Ioannina, 45110 Ioannina, Greece; 2 Structural Bioinformatics and High Performance Computing Research Group (BIO-HPC), Computer Engineering Department, Universidad Católica de Murcia (UCAM), 30107 Guadalupe, Spain; 3 Institute of Materials Science and Computing, University Research Center of Ioannina (URCI), 45110 Ioannina, Greece

**Keywords:** natural products, medicinal plants, anti-aging, anti-melanogenic, antioxidants, elastase, tyrosinase, hyaluronidase, natural inhibitors, database

## Abstract

Natural products bear a multivariate biochemical profile with antioxidant, anti-inflammatory, antibacterial, and antitumoral properties. Along with their natural sources, they have been widely used both as anti-aging and anti-melanogenic agents due to their effective contribution in the elimination of reactive oxygen species (ROS) caused by oxidative stress. Their anti-aging activity is mainly related to their capacity of inhibiting enzymes like Human Neutrophil Elastase (HNE), Hyaluronidase (Hyal) and Tyrosinase (Tyr). Herein, we accumulated literature information (covering the period 1965–2020) on the inhibitory activity of natural products and their natural sources towards these enzymes. To navigate this information, we developed a database and server termed ANTIAGE-DB that allows the prediction of the anti-aging potential of target compounds. The server operates in two axes. First a comparison of compounds by shape similarity can be performed against our curated database of natural products whose inhibitory potential has been established in the literature. In addition, inverse virtual screening can be performed for a chosen molecule against the three targeted enzymes. The server is open access, and a detailed report with the prediction results is emailed to the user. ANTIAGE-DB could enable researchers to explore the chemical space of natural based products, but is not limited to, as anti-aging compounds and can predict their anti-aging potential. ANTIAGE-DB is accessed online.

## 1. Introduction

Skin is the largest organ of the human body and is a great shield, as it protects it from external infections (environmental and chemical pollutants) as well as from UV irradiation. However, it is vulnerable since its degradation can occur both due to extrinsic and intrinsic factors, leading to early aging [1]. Among all, extrinsic skin aging, called photoaging, is a remarkable result of oxidative stress caused by UV irradiation. In addition, reactive oxygen species (ROS) have also been found to contribute to skin aging, as they are produced in skin cells through UV irradiation, although at low concentrations they could be beneficial for some signaling pathways. Environmental and chemical pollutants also produce ROS, triggering a number of pathologies [2,3]. Skin’s connective tissue includes a number of constituents, including collagen fibrils, elastic fibers, glycoproteins, and glycosaminoglycans [3]. Among all, proteins like elastin, collagen, the glycosaminoglycan hyaluronic acid, and a polymeric pigment called melanin play pivotal roles in the regulation of skin’s elasticity as well as its protection against UV irradiation.

Specifically, elastin is able to maintain the original shape of the tissues after stretching or contracting. Hyaluronic acid (HA) [4], a long chained linear molecule that is negatively charged, consists of sugar moieties connected with repeated [(1→3)-*β*-D] and [(1→4)-*β*-D] glycosidic bonds. Its main role is to stimulate the elasticity of the soft connective tissue and the joints in order to maintain the fluid of the eye vitreous, as well as to regulate the moisture through the tissues, the muscles, and the extracellular matrix [4]. HA is also involved in many cellular metabolic pathways such as cell detachment, mitosis, and migration, while it plays a lethal role in tumor development and metastasis [4]. Melanin, a dark colored polymeric pigment that is formed in melanocytes through chemical reactions caused by oxidases like tyrosinases, has the ability to protect the skin against UV irradiation as well as to form vitamin D_3_ [5]. The continuous exposure to UV irradiation causes inflammatory skin disorders, as the ROS that are produced induce damage in the neutrophils leading to the uncontrolled release of elastases. These enzymes degrade elastin, collagen, and a number of other molecules and tissues, causing not only skin aging but also many pathologies, mainly in the lung. ROS also induce the function of hyaluronidases. These are enzymes which play a pivotal role in wound healing as they regulate cell migration, differentiation, and proliferation [6]. Their main role is the degradation of hyaluronic acid into smaller saccharides. However, their uncontrolled activity on HA could lead to loss of skin moisture, triggering skin aging and various disorders. In addition, the extensive exposure to UV irradiation causes a high production of melanin in skin melanocytes through the activity of tyrosinases. During melanin synthesis, ROS are produced in melanocytes, as they include intermediates of this biochemical synthesis. However, the non-regulated production of melanin could lead to various skin disorders including hyperpigmentation, development of wrinkles, brown spots, and even melanoma [7,8]. Thus, the regulation or even the inhibition of the activity on these enzymes is of major importance for treatments against skin disorders and skin aging.

Inhibitors of HNE, Hyal, and Tyr have demonstrated effective antioxidant, anti-aging, and anti-wrinkle potential. Nature has delivered a huge library of natural compounds like polyphenols, flavonoids, cinnamic acid derivatives, lipid acids, sesquiterpenes, and numerous other groups which have elucidated effective activity against these three enzymes. Their molecular structure, along with their structural substitutions, are of great importance, as they can interact with the enzymes’ catalytic sites, enabling their inhibition. The sources of these inhibitors are mainly plants, trees, or herbs that have already been used for the treatment of various diseases. It is of importance that low concentrations of Red Cedar tree extracts were tested extensively for their potential to enhance fungi proliferationin the early 1940s [9,10]. This process has been called “hormesis”, coming from the Greek term “to excite” and is remarked by its biphasic dose responsiveness. This term implies that low doses of studied agents could promote stimulation whereas high doses could lead to inhibition [11]. Hormesis is related to aging in a physical way; despite the molecular damage that is caused by mild oxidative stress, in the first steps of the procedure many pathophysiological metabolic pathways of repair, including immune and stress response are stimulated in the cells [12,13,14]. Hormesis was widely used in the development of traditional medicinal chemistry and other fields such as biology, pharmacology, or toxicology, as a great number of polyphenolic and flavonoid compounds, called “hormetics”, were studied for the way their involvement in many cell signaling pathways could lower the rhythm of cell aging [12,15].

The study of natural products’ potential is an active research field focusing on exploring different plants, including extracts and isolated bioactive compounds, and their inhibitory properties against these targeted enzymes. Furthermore, cosmetic industries have an interest in developing new skin care products made of natural constituents as well as their originating sources, so as to promote an eco-friendly product enriched with natural antioxidants, anti-inflammatory, and anti-aging agents.

The exploitation of the chemical space of natural products as rich source of potent bioactive compounds is of supreme importance and the success rate in discovering novel bioactive agents is significant. This is especially the case for enzymes whose overexpression leads not only to skin-aging, but also to potent changes in many metabolic pathways, resulting in inflammatory disorders and even in tumor development. Human Neutrophil Elastase, Hyaluronidase, and Tyrosinase include such targets, and numerous studies on their inhibition with natural products has already been reported. Specifically, plant extracts and isolated secondary metabolites have been extensively explored from 1965 as potent anti-aging and anti-melanogenic compounds.

In this work, we navigate on information that has been accumulated on plant-based natural products for the period 1965–2020 that have been identified as inhibitors against HNE, Hyal and Tyr activity. We provide structural information on the scaffolds that have illustrated inhibitory activities on the targeted enzymes, their inhibitory values, their interaction profile with the targeted enzymes, structure-activity relationship studies, the inhibitory mechanism of secondary metabolites, as well as their binding efficiency. This knowledge with detailed information has been shaped in a user-friendly, navigable electronic database where the user could extract information concerning the natural product based inhibitors of HNE, Hyal, and Tyr. The server operates in two axes. On the first axis, a comparison by shape similarity can be performed against a curated database of natural products whose activity against these enzymes has been established in the literature. In addition, an inverse virtual screening can be performed by the user for a chosen molecule against these targeted enzymes. The server ANTIAGE-DB is accessible through https://bio-hpc.ucam.edu/anti-age-db.

## 2. Enzymes Related to Early Skin-Aging

### 2.1. Human Neutrophil Elastase (HNE)-A Serine Protease

Neutrophil elastase [EC 3.4.21.37] (known as Human Leukocyte Elastase (HLE) or elastase 2) is a glycoprotein belonging to chymotrypsin serine proteases family [16]. The active form of HNE can be found at a concentration of ~5 mM in neutrophil lysosomes (azurophils), which act as HNE carriers [17]. HNE has specificity for protein hydrolysis and is responsible for the degradation of proteins located in the azurophils, as well as extracellular matrix (ECM) proteins [17]. HNE hydrolyzes all the prime proteins constituting the connective tissue, such as collagen-IV, elastin, fibronectin, proteoglycans, keratins, and other ECM proteins [18,19]. However, elastase shows great specificity in degrading elastin, a protein responsible for the elasticity in arteries, lungs, ligaments, and skin [18,20,21]. When bacteria and microorganisms invade the ECM through wounds, the mechanism of inflammation is activated, leading to the secretion of HNE, resulting in tissue repair, proving the vital role of elastin [21,22]. Along with collagenase, elastase regulates skin tissue homeostasis. In contrast, if there is no homeostatic control, elastase degrades several connective tissue proteins [23], causing early skin aging. Furthermore, elastase plays a vital role in conditions damaging the lung tissues, leading to the development of many inflammatory disorders, such as acute respiratory distress syndrome, pulmonary emphysema, lung inflammation, and even rheumatoid arthritis.

HNE is a 30-kD glycoprotein consisting of 218 amino acids and four disoulfide bridges (Cys: 42–58, 136–201, 168–182 and 191–220) that form two stable antiparallel cylindrical domains of beta-barrels [24]. HNE shows specificity in degrading peptide bonds between small hydrophobic amino acids. The potent catalytic activity of HNE depends on a catalytic triad that contains the amino acids Histidine (His57), Serine (Ser195), and Aspartate (Asp102) (Figures S1 and S2). HNE’s structure consists of five distinguished hydrophobic pockets, four substrate-binding pockets (S2, S3, S4, and S5), and one catalytic pocket (S1) (Figure S2b) [24,25]. The S1 pocket is hemispheric and hydrophobic and binds to medium-sized aliphatic amino acids. The S2 pocket has a bowl-like shape and prefers medium-sized aliphatic amino acids like Pro. S3, S4, and S5; the pockets take part in the bond formation of HNE with the appropriate ligand. HNE’s catalytic mechanism is similar to that of the homologous serine protease chymotrypsin [26,27] and is described in Supplementary Material, Section 1.

#### 2.1.1. Natural Sources as HNE Inhibitors

Numerous natural compounds have been used in cosmetics, against skin diseases, or as anti-agings. A study involving the HNE inhibition of grape pomace polyphenols showed that the extraction method defines the extract’s inhibitory potency against HNE [28]. The polarity of the solvent also plays a major role in the inhibitory effects of a plant extract [29]. Methanol is an efficient solvent for extracting a satisfying amount of polyphenols, whereas lipophilic solvents extract constituents with higher HNE inhibitory effect [30,31,32,33]. According to a study on the HNE inhibition of bark extracts [34] from various trees, n-heptane extracts showed inhibition over 50%, implying that nonpolar bark phytochemical constituents are very active. In contrast, methanol/water extracts of the barks *F. sylvatica*, *Q. robur*, *P. avium*, and *L. decidua* also had efficient inhibitory activity. In contrast, *F. sylvatica*, *Q. robur*, *A. glutinosa*, *P. avium*, *L. decidua*, and *P. abies* methanolic extracts showed the highest PPE (Porcine Pancreatic Elastase) inhibitory activity [30,35]. However, in cosmetics there is a restriction regarding extraction solvents as they should be non-toxic to the skin, thus ethanolic and aqueous solvents are more preferred.

Experiments in the following plant extracts showed satisfying HNE inhibitory activity [21]: white tea (89%), cleavers (57.9%), burdock root (50.9%), bladderwrack (50.2%), anise (31.9%), angelica (31.6%), rose aqueous extract (24.15%), rose tincture (22.08%), pomegranate (14.64%), and green tea (9.99%). Furthermore, extracts from 150 medicinal plants have been studied for their anti-HNE potency along with their anti-inflammatory and anti-aging properties [30]. Among all the samples, 80 plant extracts showed the most potent anti-PPE activity, whereas *Areca catechu*, *Cinnamomum cassia*, *Myristica fragrans*, and *Curcuma longa* extracts showed over 30% PPE inhibition (at 100 mg/mL) and 80% PPE inhibition (at 1000 mg/mL). Additionally, six methanolic extracts from nine medicinal plants showed over 65% inhibition against different types of elastase at 1000 mg/mL: seed of *Areca catechu*, cortex of *Cinnamomum cassia*, seed of *Myristica fragrans*, radix of *Curcuma longa*, radix of *Dryopteris crassirrhizoma* and seed of *Alpinia katsumadai*. *Areca catechu* showed 33.2% PPE inhibition at 10 mg/mL, 59% PPE inhibition at 50 mg/mL, and 95% PPE inhibition at 500 mg/mL. In addition, these plant extracts showed different values of inhibition against the hydrolytic activity for both PPE and HNE. Among all, *Areca catechu* had the strongest inhibitory activity against both PPE and HNE (IC_50_: 42.4 mg/mL and 51.3 mg/mL).

Various studies reported that barks from conifers and broadleaved trees include plenty bioactive compounds that are used in cosmetics [34,36,37,38]. Barks contain numerous secondary metabolites that protect the cambium and the inner part of the trunk, which are important factors for tree growth as well as for treatment against various diseases. In a relevant study, ten barks from common deciduous and coniferous plants growing in temperate forests were studied for their PPE inhibitory activity: *Fagus sylvatica* L. (Fagaceae), *Quercus robur* L. (Fagaceae) (pedunculated oak), *Alnus glutinosa* (L.) *Gaertn.* (Betulaceae) (black alder), *Prunus avium* L. (Rosaceae) (wild cherry), *Larix decidua Mill.* (Pinaceae) (European larch), and *Populus tremula* L. (Salicaceae) (American aspen). Among all, the methanolic extracts from *F. sylvatica*, *Q. robur*, *A. glutinosa*, *P. avium*, *L. decidua*, and *P. abies* demonstrated PPE inhibitory activity.

Lastly, in 1983, Hojima et al. [39] studied a family of legumes such as Soybean (*Glycine max*), Lima bean (*Phaseolus limensis*), Red kidney bean (*Phaseolus vulgaris*), Adzuki bean (*Phaseolus angularis*), and Lentil (*Lens sculenta*) and proved that these plant species are abundant in HNE natural proteinaceous inhibitors. They isolated PSP1-21, a 21 kDa protein, from potato tubers, which showed effective inhibition towards HNE.

We present a detailed table of the reporting plants, their extracts, and the bioactive compounds which have been reported as HNE inhibitors, both in the ANTIAGE-DB and in the following link: https://bio-hpc.ucam.edu/anti-age-db/web/Info/Info.php#TableS1 (accessed on 27 September 2022). This information is also available in Supplementary Material Table S1.

#### 2.1.2. Natural Secondary Metabolites as HNE Inhibitors

**Figure 1:**
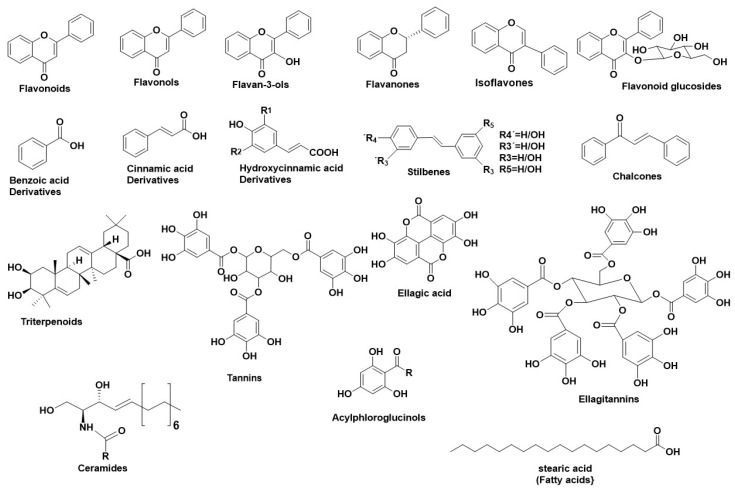
Main structural scaffolds which have been marked as potent HNE inhibitors.

Figure 1 illustrates the main structural scaffolds that have been marked as potent HNE inhibitors. Polyphenols [40] phenolic acids, cinnamic acids (and derivatives), caffeic acids (and derivatives), [18,41,42,43] stilbenes, chalcones, elagitannins [44] pentacyclic triterpenes, fatty acids [45], ceramides [46], and acylphloroglucinols have been studied extensively for their anti-HNE properties. The characteristic flavonoid structure and the number of the -OH groups affect their interaction with the HNE’s active site [47]. Gallic acid derivatives, which were estimated as potent elastase inhibitors, interacted with HNE’s active site through hydrophobic forces and hydrogen bonds [28]. Caffeic acid derivatives, such as bornyl caffeate [40,41] (a bicyclic caffeic acid derivative), also inhibited HNE efficiently (IC_50_ = 1.6 μΜ). Additionally, in silico studies revealed an interaction between the caffeic acid moiety with the amino acids of the “oxyanion hole” of HNE^40,^. Similarly, bornyl coumarate and bornyl ferulate (hydroxycinnamic acid derivatives) interacted with HNE’s active site as bornyl caffeate [40,41]. *N*-octylcaffeic acid (an alkylated caffeic acid derivative) also demonstrated strong HNE inhibition (IC_50_ = 1 μΜ). The silico studies showed that *N*-octylcaffeic acid interacted with HNE’s catalytic triad [42].

Many studies reported that the substitution of an –OH group on the C-3′ place of the flavonoid ring affects its inhibitory potency. Quercetin-3-methylether, an isolated bioactive compound from *Grindelia robusta Nutt.* [48], showed the highest inhibitory potency (IC_50_ = 19 μΜ). Additional studies on the structural properties of the isolated compounds from *G. robusta* have demonstrated that a free -OH group on the C-3′ increases the anti-HNE activity, while the substitution of other groups (such as a methylic group), reduces the anti-HNE activity. The isolated compound quercetin-3, 3′-dimethylether, which lacks -OH groups at the C-3′, showed lower anti-HNE potency (IC_50_ = 129 μM), while quercetin-3, 6-dimethylether, which contains a free -OH group at the C-3′, shows higher anti-HNE activity (IC_50_ = 115 μΜ). In contrast, a -OH substitution at the C-2 of flavonoids also enhances their inhibitory potency. Studies made on the isolated secondary metabolites of *Campylotropis hirtella* (Leguminosae) [49] proved that fact. Additional studies on the isolated compounds of *C. hirtella* revealed also that the presence of a geranylated group enhances their anti-HNE activity. Furthermore, substituted prenyl A-rings of flavonoids showed strong inhibition towards HNE. Studies on the isolated bioactive compounds of *Flemingia Philippinensis* [50] revealed several elastase inhibitors. Further studies correlated the inhibitory potency with the structure; for example, the presence of two prenyl groups in the A-ring of 8-γ, γ-dimethylallylwighteone, osajin, and flemingsin enhanced their anti-HNE potency (IC_50_ = 6.0 μΜ, 26 μΜ, and 12 μΜ respectively) compared to the main compound genistein (IC_50_ = 51.4 μΜ). In contrast, the presence of the prenyl group in the B-ring decreased the anti-HNE potency, as in 5, 7, 3′, 4′-tetrahydroxy-2′, 5′-di (3-methylbut-2-enyl) isoflavone (IC_50_ = 213.1 μΜ). Additionally, the presence of a catecholic moiety enhances the inhibitory potency. The results showed that the catecholic group in the B-ring of 6, 8-diprenylorobol enhanced its inhibitory activity to 1.3 μΜ. Further studies showed that a resorcinol group in the B ring, as well as a 4-hydroxy group in flavanones, increased their inhibitory activity, as in flemichin D (IC_50_ = 5.3 μΜ) and lupinifolin (IC_50_ = 13.3 μΜ).

Essential oils have also demonstrated efficient HNE inhibition. Citral, thymol, geranial, and geraniol consist of main HNE inhibitors according to studies made on their inhibitory potency towards *Pseudomonas* elastase [51]. Docking results showed that thymol had the best binding profile, whereas geranial, geraniol, and citral also showed great affinity towards *Pseudomonas* elastase. According to this study, essential oils from plants such as *Cymbopogon citratus*, *Cymbopogon martini*, *Rosmarinus officinalis*, *Mentha piperita*, *Pelargonium odoratissimun*, and *Virtex negundo* are important elastase targets [51].

Stilbenes also include potent elastase inhibitors. Resveratrol, an abundant compound in grapes, showed strong HNE inhibition (IC_50_ = 12–31 μΜ) [40,43]. According to the in silico studies, resveratrol interacted with HNE through a phosphorylated tyrosine residue [40,43]. In addition, resveratrol was also found to act as a homertic agent regarding cell aging [52].

Regarding fatty acids, X-ray crystallographic techniques showed that a small chained (up to 15 carbon atoms) saturated fatty acid has effective interaction with HNE, leading to a 50% inhibition. The interactions take place through hydrophobic bonds with at least one Arg [39]. Thus, fatty acids which bind with more than one Arg are more appropriate HNE inhibitors.

Ceramides (a family of waxy lipids) [46] are found in high concentrations within the cell membrane and participate in several cellular signaling processes, such as regulating differentiation, proliferation, and programmed cell death. They also balance the function of the skin barrier as well as maintain the water levels in the stratum corneum [53,54]. Ceramides have reported to form hydrophobic interactions with the hydrophobic pockets near HNE’s active site [55]. Studies have indicated that the plant ceramides dehydrophytosphingosine, phytosphingosine, and dihydrosphingosine, along with polyunsaturated fatty acids [56,57], could be HNE inhibitors. In vitro and ex vivo experiments performed using glucosylated and non-glucosylated ceramides extracted from wheat showed that the non-glycosylated ceramides are more potent HNE inhibitors than the glycosylated ones.

Lastly, phloroglucinols, especially acylphloroglucinols, were also studied against HNE activity. Three acylphloroglucinols have been evaluated for their anti-HNE potency when stimulated with fMLP from neutrophils: myrtucommulone, semimyrtucommulone (*Myrtus communis* leaves extracts), and hyperforin (*Hypericum perforatum* extracts) [55] with low IC_50_ (0.4–3.8 μΜ) [58,59].

We present the most important secondary metabolites which were studied for their inhibitory properties towards HNE in ANTIAGE-DB at the following link: https://bio-hpc.ucam.edu/anti-age-db/web/Info/Info.php#TableS2 (accessed on 27 September 2022). This information can also found in Supplementary Material Table S2.

### 2.2. Hyaluronidase (Hyal)—A Glycosyl Hydrolase

Hyalouronidases (Hyals) [E.C. 3.2.1.35] consist of a subclass of glycosyl hydrolases mainly responsible for degrading the glycosaminoglycan Hyaluronic Acid (HA) [60] by cleaving its *β*-1→4 glycosidic bonds into oligomers. HA can be found in abundance in synovial fluid, in cartilage (mainly in the soft part of connective tissue), and in the vitreous part of the eye [61]. Furthermore, HA can be found in the extracellular matrix as a connecting fluid of protein filaments, collagen fibers, and connective tissue cells [62].

Studies made on Hyals by Karl Meyer (1971) [63] differentiated them into three groups according to their reaction mechanism: (1) bacterial hyaluronidases (E.C. 4.2.99.1) (endo-*β*-acetyl-hexosaminidases), (2) hyaluronidases (E.C. 3.2.1.36) (endo-*β*-glucuronidases), and (3) mammalian hyaluronidases (E.C. 3.2.1.35) (hydrolases, producing tetrasaccharides and hexasaccharides).

Hyals contain five homologous molecules expressed in the human gene: Hyal-1, Hyal-2, Hyal-3, Hyal-4, and Hyal-5 (SPAM-1 or PH-20), as well as a pseudogene (pHYAL1) [60]. Hyal-1, Hyal-2, and Hyal-3 are found in chromosome 3p21.3 [64], whereas Hyal-4, PHYAL1, and SPAM1 are found in chromosome 7q31.3 [65]. Hyal-1, Hyal-2, and PH-20 regulate HA’s stability [66], whereas Hyal-3, Hyal-4, and PHYAL1 do not affect HA’s metabolism [67]. Among all Hyals, human Hyal-1 is abundant in the somatic tissue and in human serum (~60 ng/mL) as well as in liver, kidney, heart, plasma etc. This lysosomal enzyme contains three *N*-glycosylated sites at places N-99, N-216, and N-350 [68] and degrades HA into GlcA-GlcNAc-GlcA-GlcNAc tetrasacharides [61]. According to numerous studies, Hyal-1 is expressed in various cancer cells, like in the prostate, bladder, and brain [69,70], and is also connected to the human cancer cell lines MCF-7 and MDA-MB-231 [71]. Hyal-1 is of great interest, as a well-developed study is determining compounds which act as natural Hyal-1 inhibitors against diseases like arthritis and gingivitis [72,73]. Hyal-2 is widely found in connective tissue at high concentration and decomposes HA into oligomers of 50 saccharide units (~20 kDa) [74]. The activity of Hyal-2 ranges between various pH values, whereas Hyal- 1 and Hyal-3 are active in acidic conditions. Hyal-4 behaves also as a chondroitinase, using Chondroitin (Ch) and Chondroitin Sulfate (ChS) as substrates. Additionally, SPAM1 is located in the acrosome of spermatids and enables the adhesion of sperm to the cumulus mass protecting the ovum [74,75].

#### 2.2.1. Structure of Hyals

Hyals’ catalysis (mechanism described in Supplementary Material Section 2) depends on an acidic active site Glu-131, which donates H [60]. Hyal’s active site also contains Tyr75 Trp141, Tyr201, Tyr208, Tyr210, Tyr247, Tyr261, Tyr286, and Trp321, which are responsible for its catalysis (Figure S3). The active site, positively charged hydrophobic amino acids enable the complete interaction not only with HA, but also with other negatively charged substrates (e.g., Chondroitin and Chondroitin Sulfate) [74]. Furthermore, Hyal’s structure contains two arginines (Arg134 and Arg265), which interact with -COOH groups of HA, as well as a Ser245, which is stable and enables bond formation with the −OH group of Tyr202 [61,68,74]. Hyal-4 has Cys263 instead of Tyr247, which enables the interaction with Chondroitin and Chondroitin Sulfate [74].

#### 2.2.2. Natural Sources as Hyal Inhibitors

Numerous plants and their extracts have been studied for their anti-Hyal properties. *Padina pavonica* Macroalga [76], a species of brown alga found in Mediterranean and Atlantic ocean, has shown efficient anti-Hyal activity through enzymatic studies. Another study made on the anti-Hyal activity of brans, like sorghum bran (highly antioxidant grain), wheat bran, and rice bran [77], showed efficient anti-Hyal activity. The bioactive constituents of sorghum include phenolic acids (benzoic and cinnamic acid derivatives), flavonoids (3-deoxyanthocyanins, flavan-4-ols, flavones and flavanols), and condensed tannins. Some samples lacked tannins (Black, Fontanelle, Mycogen, and White), while the other group was abundant in tannins (Suman and Shanqui Red). The results evaluated a dose-dependent inhibition with Suman sorghum as the strongest inhibitor (50% inhibition at 90 μg dry weight/0.5 mL). The other samples inhibited Hyal in decreasing order: Sumac > Shanqui Red > Black > Mycogen > Fontanell > White sorghum. The results showed that the presence of condensed tannins enhanced their anti-Hyal activity. All the plants, their extracts, and their isolated bioactive compounds, which were evaluated for their anti-hyaluronidase activity, are represented in ANTIAGE-DB in the following link: https://bio-hpc.ucam.edu/anti-age-db/web/Info/Info.php#TableS3, as well as in Supplementary Material Table S3.

#### 2.2.3. Natural Secondary Metabolites as Hyaluronidase Inhibitors

**Figure 2:**
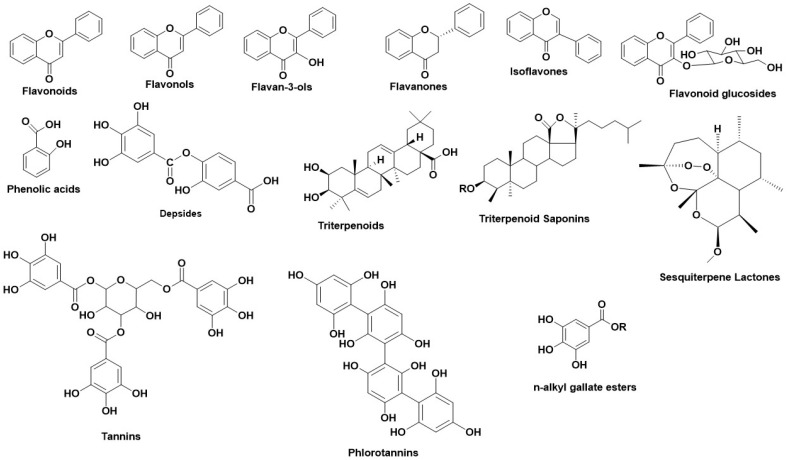
Main structural scaffolds which have been marked as potent Hyal inhibitors.

Numerous studies regarding the anti-Hyal potency of plants, their extracts, or their isolated metabolites have showed that the most potent natural inhibitors are terpenes, flavonoids and alkanoids [60]. Figure 2 illustrates the main structural scaffolds which have been marked as efficient Hyal inhibitors.

Terpenoids consist of a family with high anti-Hyal potential. Among all, glycyrrhizin and glycyrhetinic acid [60] have demonstrated efficient inhibition against bacterial Hyal B (*Streptococcus agalactiae*) and rHyal B (recombinant Hyal from *S. agalactiae*). Glycyrrhizin was found to be a strong, noncompetitive Hyal inhibitor (IC_50_ = 0.440 mM for Hyal B and IC_50_ = 0.455 mM for rHyal B), while glycyrhetinic acid inhibited Hyal B and rHyal B with an IC_50_ value of 0.060 mM and 0.080 mM, respectively. Additional structure–activity studies showed that glycyrrhizin formed stronger bonds with rHyal B due to the fact that the inhibitor was placed outside the active site.

Glycosidic triterpenoid saponins have also been studied for their anti-Hyal properties [65]. Potent human Hyal-1 inhibition was demonstrated for *Gypsophila* saponin 2 (IC_50_ = 108 μΜ) as well as for the saponins SA1657 (IC_50_ = 371 μΜ) and SA1641 (IC_50_ = 296 μΜ). The results implied that the –OH substitution at C-16 affected the inhibitory potential. Furthermore, sesquiterpene lactones were also reported as strong Hyal inhibitors [78].

The presence of metal ions in Hyal’s active site plays crucial role for its activity, as many chelating ligands have the ability to form coordinate covalent bonds that lead to Hyal’s inhibition. Studies made on the essential oils from *Melaleuca leucadendron Linn* [60,79] revealed a strong Hyal inhibitor: β-caryophyllene (IC_50_ = 4.16 μg/mL), as it interacted through chelated bonds with Hyal’s metal cofactor Cu^2+^ [80].

The vital antioxidant character of a phenolic -OH group has been studied extensively. Thus, phenolic acids and flavonoids, including their subclasses, have been evaluated for their anti-Hyal potency [60]. The number and the location of -OH groups in the flavonoid core have been reported to affect their inhibitory potential. The presence of an -OH group in C-3, C-3′ as in quercetin and C-5 as in myricetin, enhance Hyal’s inhibition [81]. Other studies showed that the presence of numerous free -OH groups in the flavonoid ring, as well as the length of the side-chains in the substituted positions, affect the inhibitory activity, implying that a free aglycone structure is a more efficient inhibitor than its respective glycoside [82]. Isoliquiritigenin, a flavone isolated from *Glycyrrhiza glabra* [83], showed potent Hyal inhibition (IC_50_ = 64 μΜ). According to docking results [84] liquiritigenin formed hydrophobic forces with Hyal’s active site through Tyr75, Val127, Tyr202, Tyr247, Tyr286, and Trp321. Additionally, both hydrophobic and electrostatic forces were formed through Asp129, Glu131, Gln288, and Asp292 in Hyal’s active site. These results were in accordance with the thermodynamic values calculated from different spectroscopic methods [84].

Depsides, a subclass of phenolics have also been studied as Hyal inhibitors. Chlorogenic acid (esterified derivative of caffeic and (-) quinic acid) [60], found in abundance in many plants, has shown efficient anti-Hyal potency. Chlorogenic acid interacts efficiently with Hyal’s active site, as, according to in silico docking results, the phenyl moiety induces hydrophobic interaction with Ala407 and Val411 [60]. Furthermore, caffeic acid also showed potent Hyal inhibition [60]. The two catecholic -OH groups interact through hydrogen bonds with Glu477 and Glu582, leading to Hyal’s inhibition [60]. Experiments on gallic acid showed that it also performs anti-Hyal potential. In addition, gallic acid ester derivatives are also reported to be potent Hyal inhibitors. Although gallic acid esters are lacked in nature, several *n*-alkanol gallate esters have been synthesized and evaluated for their anti-Hyal activity [85].

Tannins [60] have also been studied for their inhibition towards Hyal. According to in silico studies, phlorotannins [60], due to their molecular size, cover exclusively the active site of Hyal leading to its inhibition [60].

All the natural secondary metabolites which have been tested for their anti-Hyal activity are represented in ANTIAGE-DB at the following link: https://bio-hpc.ucam.edu/anti-age-db/web/Info/Info.php#TableS4 (accessed on 27 September 2022), as well as in Supplementary Material Table S4.

### 2.3. Tyrosinase—A Polyphenol Oxidase

Tyrosinases (catechol oxidases, catecholases, diphenol oxidases, *o*-diphenolases, phenolases and polyphenol oxidases) [ΕC 1.14.18.1] are copper-containing glycoproteins and can be found in various active forms in living organisms (mammals, plants, fungi and animal tissues) [86]. The most vital activity of Tyrs is their implication in melanin and other pigments synthesis, as well as the brown color in fruits and vegetables, caused by the oxidation of the present Tyr in their flesh with the atmospheric molecular oxygen. In humans, Tyrs are located in the melanosomes, which are synthesized in the skin melanocytes. Tyrs have a double enzymatic activity, in the presence of O_2_: (1) they act as monophenolases (cresolases) and hydroxylate monophenols in their ortho-position and (2) they act as diphenolases (catecholases) and oxidize *o*-diphenols into *o*-quinones. Tyr oxidizes L-tyrosine into dopaquinone, a product which leads to the formation of eumelanin (brown or black melanin) and pheomelanin (red to yellow melanin) [86].

#### 2.3.1. Structure of Tyrosinase

Specifically, Tyr structure consists of three domain parts. The central, and most characteristic domain, is the active site binuclear copper moiety, which enables the bond formation between Tyr and molecular O_2_, as well as with Tyr’s substrate [87]. The other domains contain six histidines, which form bonds with the cuprous pair inside the active site (Figure S4). The Tyr structure is stabilized through disoulfide bridges, formed by cysteines around the active site. Tyrs from different plants differ in their number of cysteines; human and mouse Tyrs have 17 cysteines, whereas plant Tyrs have 11 cysteines. Furthermore, plant Tyrs contain one cysteine in their Carboxylic terminal moiety, whereas Tyrs from *N. crassa*, *A. bisporus*, and prokaryotic Tyrs have 0 or 1 cysteines [88].

#### 2.3.2. Natural Sources as Tyr Inhibitors

All the identified natural plants and their extracts which have been studied as potent Tyr inhibitors, are represented in ANTIAGE-DB at the following link: https://bio-hpc.ucam.edu/anti-age-db/web/Info/Info.php#TableS5 (accessed on 27 September 2022), as well as in Supplementary Material Table S5.

#### 2.3.3. Natural Secondary Metabolites as Tyr Inhibitors

**Figure 3:**
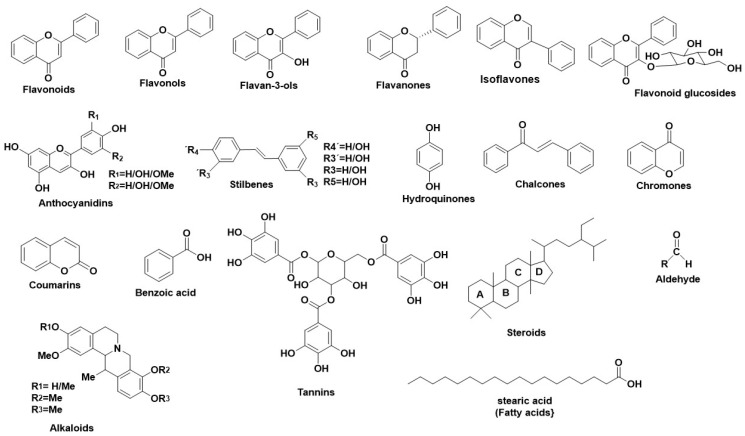
Main structural scaffolds which have been marked as potent Tyr inhibitors.

Numerous studies revealed various natural metabolites as Tyr inhibitors, along with their interacting properties with Tyr’s active site through combined studies on docking simulations and kinetics [89]. The structure of Tyr is similar to hemoqyanin, and a lot of correlated results regarding the binding properties of Tyr can be excluded [89,90,91]. Wang et al., developed a fluorescent probe with which they determined the mechanism of Tyr reaction [92] finding that the inhibitors react with Tyr irreversibly [86]. Tyr inhibition is achieved either by covering the active site or by producing undesirable dopaquinone derivatives or by completing multisite reactions. For this reason, several studies have been performed regarding the inhibitory activity of natural compounds, their type of inhibition, including also their interaction profile. Figure 3 illustrates the main structural scaffolds which have been marked as efficient Tyr inhibitors.

Flavonoids [86]: Representative Tyr inhibitors are kaempferol [93] quercetin [94,95]. Flavonoids with a free 3-OH group in their structure form chelated copper complexes within Tyr’s active site [96]. Flavonoids without 3-OH group (luteolin 4′-*O*-glucoside, luteolin 7-*O*-glucoside), act as Tyr inhibitors [97]. Additional studies on flavonoids [98] reported that the presence of a keto group affects Tyr inhibition. Of importance is the fact that the difference in the conjugation of the A and B flavonoid rings and the number and type of the substituted positions (hydroxyl, methoxy, glycosides etc.) affect the function of the flavonoid; it can act either as a Tyr inhibitor or substrate. Kaempferol [93], quercetin, and morin act as a Tyr inhibitor [99], whereas catechin acts as a substrate [99].

Gallic acid [100] showed significant anti-Tyr activity, both in its free and in its esteric form. Gallic acid esters with D-glucose are abundant in green tea [101] and *Galla rhois* and are common food additives [102,103]. According to studies, the presence of a galloyl group in C-3 of a flavon-3-ol structure, affects Tyr inhibition. 1,2,3,4,6-Penta-*O*-galloyl-D-glucose (PGG, extracted from *G. rhois*) was found to be strong Tyr inhibitor, although some studies supported that esterified, hydroxylated, and methylated aromatic carboxylic acids have weak anti-Tyr activity [104,105]. Additionally, the length of gallic esters’ carbon chain regulates Tyr inhibition. Hydrophobic long chained (above 10 carbon atoms) gallic acid esters act as Tyr inhibitors, whereas smaller chained gallic acids behave as Tyr substrates and get oxidized due to the fact that large sized gallates can’t fit in the tetartiary structure of Tyr [100]. According to reaction mechanism analysis on carboxylic acid derivatives, they develop inhibitory character through an interaction between the carboxylic acid and Tyr’s binuclear site, leading to a copper-carboxylic complex [106]. Furthermore, a *para*-substituted phenolic compound enhances the anti-Tyr activity [107]. In addition, various phenolic acids, such as gallic acid or protocatechuic acid, have also been studied for their homertic activity against signaling pathways related to aging [108].

Rhamnetin (*O*-methylated flavonol) has been tested for its anti-Tyr potency. According to a study made on the murine Tyr inhibition on B16 cells [109], rhamnetin inhibited murine Tyr by 30.6% at 5 μΜ, 63.3% at 20 μΜ, and 75.5% at 40 μΜ, respectively.

Studies on bioactive compounds like cardol derivatives [94] indicated that the –OH groups increases their anti-Tyr potency, whereas their replacement by –CH_3_ groups decreases their anti-Tyr potency. Furthermore, the presence of an unsaturated alkyl side chain increases the anti-Tyr activity when compared with the relevant saturated chain.

The presence of a 4-substituted resorcinol moiety in the phenolic ring enhances the anti-Tyr activity. Resorcinol reacts with monooxygenase, leading to the formation of 3-hydroxy-ortho-quinone which can form metal bonds with the active sited copper atoms [86,110].

Three highly antioxidant flavanols found in abundance in tea, (+) catechin, (−)-epicatechin gallate (ECG) and (−)-epigallocatechin-3-*O*-gallate (EGCG) have demonstrated potent anti-Tyr potential (IC_50_: 57.12 μΜ, 22.63 μΜ, and 142.40 μΜ, respectively). [111] The structure of each inhibitor affects the rate of inhibition [111]. Fluorescence spectroscopy confirmed that these flavanols bind to Tyr’s active site. Additional cyclic voltammetry studies showed that each flavanol follows different inhibitory mechanism; catechin binds to Tyr with low affinity and inhibits it irreversibly, though it behaves as a Tyr substrate. (−)-epicatechin gallate also behaves as a substrate, though it shows higher affinity and inhibits Tyr either through binding to ring B (catechol moiety) or ring G (pyrogallol moiety). (−)-epigallocatechin-3-*O*-gallate follows competitive inhibition. Another study proved that (−)-epigallocatechin-3-*O*-gallate eliminated the function of B-16 melanoma cells [112]. Further molecular docking studies showed that these three flavanols interact with the Tyr’s catalytic center through ring B, directly affecting the strength of inhibition.

Two flavonoids isolated from *Artocarpus xanthocarpus* Merr. [113], steppogenin and norartocerpetin, were found to present enhanced anti-Tyr potential due to the presence of a resorcinol moiety. Although the substitution in C-3 enhances the anti-Tyr activity [114], the presence of a resorcinol moiety without a substituted C-3 place strengthens the anti-Tyr activity [5]. In contrast, a resorcinol moiety in ring B also enhances the anti-Tyr activity, as in albanin A and cudraflavone C.

Studies on 3-hydroxyphloretin and catechol (flavonoids extracted from Malus doumeri) [115] found that the catecholic moiety interacts with the copper ions of Tyr, leading to inhibition [116,117]. In addition, these compounds reduced cell viability by 17% for 3-hydroxyphloretin and 2% for catechol. In the same study, additional kinetic studies for 3-hydroxyphloretin and catechol on mushroom Tyr utilized their mechanism. 3-hydroxyphloretin performed competitive inhibition in a dose-dependent way using L-tyrosine as substrate. The K_m_ and V_max_ values without an inhibitor were estimated as 3.9 × 10^2^ μΜ and 1.7 × 10^−2^ μΜ/min, respectively, and with 3-hydroxyphloretin 1.8 × 10^3^ μΜ and 1.2 × 10^−2^ μΜ/min, respectively. In contrast, catechol lacked mushroom Tyr inhibitory character in vitro [118]. Without L-tyrosine, catechol can produce a derivative with 27% efficiency, behaving as a substrate in in vitro assays. Furthermore, 3-hydroxyphloretin and catechol were tested with a model docking analysis for their interaction mechanism with Human Tyrosinase, using L-DOPA as substrate. According to docking results, the two -OH groups of 3-hydroxyphloretin formed hydrogen bonds with Ser380 and Ala486, whereas one catecholic -OH group formed chelated bonds in a monophenolase or ortho-diphenolase way [119]. In contrast, catechol can use both its catecholic -OH groups for metal binding with the copper ions, as it has small molecular structure and can adjust to Tyr’s active site.

Puerarin (hydroxyisoflavone isolated from *Pueraria lobata* Ohwi) [120] has also been studied for its anti-Tyr properties. The numerous -OH groups in puerarin enhanced the interaction with Tyr. The 6″-OH, 1″-OH, 4′-OH, and 7-OH groups of puerarin formed hydrogen bonds with His244, Arg268, His244, and Glu322, respectively, while the guanidyl cation of Arg268 formed π-cation binding with the ring B.

Flavanones substituted with a geranyl moiety demonstrated potent Tyr inhibition, as it has been shown for an isolated geranyl flavanone, along with a neuroflavone from *Campylotropis hirtella* (Leguminosae) [49]. According to in silico docking results, neuroflavone binds to the active site, as well as to the two copper ions, through the 7-OH group of ring B. This interaction was enhanced due to p-p stacking forces between ring A with H263 and V283. The ring D formed hydrophobic bonds with V283. In contrast, the 20-OH group of ring B of an isolated geranyl isoflavanone interacted through hydrogen bonds with H244 [49].

Glycosidic flavonoids isolated from Cakile Maritina Scop [121] were studied for their interaction profile with Tyr. In silico docking studies were performed on quercetin-hexoside-dihexoside, quercetin-dihexoside, kaempferol-hexoside-rutinoside, and kaempferol-dihexoside-hexoside. Quercetin-hexoside-dihexoside formed a coordinated bond with Cu401 and π-π stacking forces with His61 and His244. Hydrogen bonds were also formed with His263 and Arg268 (ΔG = −26.46, Chem Score: 1.29). Kaempferol-hexoside-rutinoside formed metallic bonds with Cu400, hydrogen bonds with His85 and His296, π-π stacking with Phe264, and cationic π-stacking with Arg268 (ΔG = −24.33, Chem Score: 5.16). Kaempferol-dihexoside-hexoside formed metallic bond with Cu401, hydrogen bonds with His61, His85, and Arg268 (ΔG = −25.98, Chem Score: 15.44). In contrast, quercetin-dihexoside formed hydrogen bonds with Asn81, Gly281, and Ser282; π-π stacking with His263 and Phe264; and cationic π bond with Arg268 (ΔG = −8.87, Chem Score:1.29). Kaempferol-hexoside-rutinoside had the best score as its structure lacked steric factors.

Hydroquinone and derivatives also consist of potent Tyr inhibitors [86]. 1,4 hydroquinone (widely distributed in tea, berries, beer, and coffee), is a very common anti-hyperpigmentating agent [122]. Studies showed that hydroquinone forms covalent bonds with histone, and it connects with copper in Tyr’s active site [123]. However, its use as cosmetic agent is forbidden in many countries because of its toxicity, whereas hydroquinone derivatives like β-arbutin (β-D-glucopyranoside of hydroquinone), deoxyarbutin (synthetic derivative of hydroquinone), and mequinol (monomethyl ether of hydroquinone) are used as cosmetic agents [124]. Studies on β-arbutin showed that its Tyr inhibition rate depends on its concentration [124]. Other studies showed that if arbutin is used with aloesin, they both act synergistically, although arbutin inhibited Tyr competitively, whereas aloesin inhibited Tyr non-competitively [125]. Though the hydrophilicity of aloesin disables penetration into the human skin, approximate concentration of aloesin inhibits Human Tyr, according to studies [126]. Concerning mequinol, it acted as a Tyr substrate [127].

Chalcones [86] (specifically 1, 3-diaryl-2-propen-1-ones) also include efficient Tyr inhibitors. Their aromatic rings contain hydroxyl, methoxy, alkyl substituents, which play a vital role in chalcones’ biological activities [128]. Licochalcone A, a phytochemical extracted from *Glycyrrhize* species, was found to be a strong mushroom Tyr inhibitor. Kuraridin [129], kuraridinol [130], and 2,4,2′,4′-tetrahydroxy-3-(3-methyl-w-butenyl) chalcone were found to be potent Tyr inhibitors. According to studies [131], a 4-substituted B ring was evaluated as more preferable than a substituted A aromatic ring. In contrast, a study showed that the presence of a resorcinol moiety in ring B increases its inhibitory potency [117]. According to SAR results, the catecholic moiety on ring A forms chelic bonds with copper and can behave as a competitive Tyr inhibitor, whereas a catecholic moiety in ring B causes oxidation of o-quinone. It was found that the most potent Tyr inhibitor is 2,4,2′,4′-tetrahydroxychalcone (IC_50_ = 0.02 μΜ). A study made on prenylated chalcones and flavones extracted from wood of *Artocarpus heterophyllus* [132] found the phytochemical morachalcone A, which showed efficient anti-Tyr potency (IC_50_ = 13 nM). Chalcones isolated from *Flemingia philippinensis* [133] (known as fleminchalcones A-C) showed structure dependence inhibition on Tyr. Despite the lack of an α, β-unsaturated ketonic moiety, these compounds present strong monophenolase inhibition. Additionally, the isolated Flemichin D has 2 -OH groups in the resorcinolic ring B, reporting the strongest inhibitory activity. On the other hand, khonklonginol H, with an -OMe group, showed lower inhibition than flemichin D.

Resveratrol [134], oxyresveratrol [135], chlorophorin [136], and andalasin A [137] (members of stilbenes family) have shown potent anti-Tyr activity with resveratrol to be the strongest Tyr inhibitor (IC_50_ value 32-times higher than kojic acid). The strong inhibitory activity of oxyresveratrol depends on the presence of a 4-resorcinol moiety in ring B, a 5-resorcinol unit in A ring, and several -OH groups in both rings [137]. On the contrary, resveratrol lacks a 4-resorcinol moiety and shows weaker inhibitory potential at about 50-times lower than oxyresveratrol. Dihydroresveratrol demonstrated weaker inhibition than resveratrol [138], but it showed stronger inhibitory activity towards mushroom Tyr than oxyresveratrol [139].

Kojic acid [86] is abundant in fungi and is used widely in cosmetics as a skin-whitening agent and in the food industry as an anti-browning agent [140,141]. Cu^2+^, Fe^2+^ and other transition metal ions, form chelic bonds with kojic acid, leading to both competitive inhibition of monophenolase and mixed type inhibition of diphenolase activity [142,143]. Because of its instability, kojic acid is not used as a cosmetic agent, but a derivative; kojic dipalmitate has fruitfully replaced it, though not validated as a relevant inhibitor. Due to its strong inhibitory behavior, kojic acid is used as a control sample when phytochemicals are studied as Tyr inhibitors.

Chromones also include potent Tyr inhibitors. Aloesin (a hydroxychromone glucoside) is extracted from *Aloe vera* [144] and has been found to show anti-hyperpigmentation activity. In addition, it can react as a co-factor with arbutin [145]. According to studies, aloesin performed more murine anti-Tyr activity than mushroom Tyr. However, it is a common substance in cosmetics.

Coumarins (2H-1benzopyran-2-ones) [87] and their derivatives have been studied as Tyr inhibitors. According to the studies, esculetin (a 6,7-dihydroxyl coumarin, extracted from *Euphorbia lathyris*) [146] was found to be a substrate of mushroom Tyr. Its inhibitory value is 25% lower than kojic acid [146]. 9-hydroxy-4-methoxypsoralen (extracted from *Angelica dahurica*) [147] demonstrated 6-times higher inhibition than kojic acid, whereas 8′-epi-cleomiscosin (an extract from *Rhododendron collettianum*) [148] demonstrated 13-times higher inhibition than kojic acid. P-coumaric acid was found to show mushroom anti-Tyr activity 10-times higher than kojic acid, and caffeic acid showed mushroom anti-Tyr activity 3-times higher than kojic acid. According to studies, p-coumaric acid [149] eliminates both the monophenolase and diphenolase function. An -OH group in the *para* position of p-coumaric acid enhances the anti-monophenolase activity but eliminates the anti-diphenolase activity.

Benzoic acid and derivatives [86] (benzaldehyde [150], benzoic acid [151], anisic acid [152], anisaldehyde [153], cinnamic acid [154], methoxycinammic acid [155], and vanillic acid [156] also perform anti-Tyr potency through different mechanisms, though lower tan kojic acid. Benzaldehyde compounds, when interacting with the amino groups of Tyr, produce a Schiff base [157], whereas benzoate compounds chelate the copper ions in Tyr’s active site, leading to its inhibition [158].

A furan derivative, (2′R)-2′,3′-dihydro-2′-(1-hydroxy-1-methylethyl)-2,6′-bibenzofuran-6,4′-diol (DHMB) [159], isolated from *Morus notabilis schneid,* has been demonstrated potent to mushroom Tyr. In a mushroom Tyr inhibition assay [160], DHMB acted as a Tyr inhibitor in a dose-dependent way. Moreover, kinetics results reported that DHMB inhibits Tyr competitively. According to docking results, DHMB interacted with the one active sited copper ions with a bond length of 2.7Å. In contrast, DHMB acted as a competitive inhibitor due to its ability to form chelated bonds with its –OH group and the copper ion, as well as to bind through p-p interaction to His263 [161,162].

Lipids and fatty acids [86] like trilinolein [163], soyacerebroside I [164], and *trans*-geranic acids [165] demonstrated low anti-Tyr activity. According to studies, lipids interact with Tyr through a free radical scavenging mechanism by unsaturated alkenes or through bonds with the outer part of the catalytic domain.

Steroids [86] are of important biological interest as they are implicated in vital metabolic reactions. Representative steroidic Tyr inhibitors are stigmast-5-ene-3β, 26-diol [166], isolated from *Trifolium balansae*; 3β,21,22,23-tetrahydroxycycloart-24(31),25(26)-diene [167], isolated from *Amberboa ramose*; and arjunilic acid [168], isolated from *Rhododendron collettianum*. All of them showed anti-Tyr activity with IC_50_ values of 12-, 13-, and 17- times higher than kojic acid, respectively. Steroids containing hydrophobic big-chained lipids, could be effectively used as skin-whitening agents, though they are not so commonly used in cosmetics.

Anthraquinones are known anti-inflammatory, wound healing, analgesic, antipyretic, antimicrobial, and antitumor agents. Additionally, they showed anti-Tyr properties. Physcion demonstrated similar anti-Tyr activity to kojic acid [168]. On the contrary, 1,5-dihydroxy-7-methoxy-3-methylanthaquinone consists of hydroxyl and methoxy groups in different positions, which enhanced its anti-Tyr activity (72-times stronger than kojic acid) [169]. Furthermore, isolated lignans from *Vitex negundo*, like (+)-lyoniresinol, showed 5-times higher Tyr inhibitory activity than kojic acid [170].

Aldehydic derivatives [86] also include efficient Tyr inhibitors as their carbonyl groups form characteristic nucleophilic reactions with the primary amino moieties of Tyr, leading to a Schiff base. Tyrosinase inhibitors from this group are the following: *trans*-cinnamaldehyde [171], (2E)-alkenals [172], 2-hydroxy-4-methoxybenzaldehyde [173], anisaldehyde [157], cuminaldehyde, cumic acid [157,174], 3, 4-dihydroxycinnamic acid, and 4-hydroxy-3-methoxycinnamic acid [155]. According to a study made between different aldehydes and their carboxylic acid derivatives like cinnamic acid, anisic acid, cummic acid, and benzoic acid, it has been showed that cuminaldehyde had the most potent anti-Tyr activity [157] as the nucleophilic cuminaldehydic para-substituents held steady the Schiff base product in Tyr’s active site. Regarding the (2E)—alkenals, their successful interaction with Tyr was due to hydrophobic forces between the long carbon chain with the binuclear copper domain [107,158,172]. Additionally, a study by Jimenez et al. [106] confirmed that 4-substituted aromatic aldehyde derivatives oxidate L-DOPA competitively, whereas the former aldehydes were shown to behave as noncompetitive Tyr inhibitors [157,173,175]. Catechin and epicatechin [112,176], two major tannins abundant in green tea and also widely distributed in various plants such as *Vigna angularis* [177], have demonstrated excellent anti-Tyr activity. According to molecular docking studies, catechin interacted with the B chain of Glu356 through hydrogen bonds and formed hydrophobic interactions with the B chain of Trp358, Lys376, and Lys379 of Tyr. On the contrary, epicatechin interacted with the A chain of Gln44 and Lys158 through hydrogen bonds, while it formed hydrophobic interactions with the A chain of Lys180 [177].

Several plant triterpenoids have also shown anti-Tyr potency. An isolated triterpenoid from *Dillenia indica* [178] has been studied for its anti-Tyr potency, whereas its interaction profile showed binding with Tyr through hydrogen bonds with His259, His263, Met280, and Asn260.

Alkaloids also include various potent Tyr inhibitors. According to docking stimulations, caffeine, a major component of *Camellia Pollen* [179], forms hydrogen bonds with Lys379 and Lys376; hydrophobic forces with Trp358; and Van der Walls interactions with Asp357, Gln307, Glu356, Asp312, and Thr308, whereas it moves one of the two Cu^2+^ ions. Additional Circular Dichroism (CD) studies revealed that the secondary structure of Tyr changed when interacting with caffeine. The binding of caffeine to Tyr promoted a conformational change of a loop so as to fit in Tyr’s active site. Two pseudoalkaloids isolated from the herb *Leonurus japonicas* (Yi Mu Cao) [180] (10-methoxy-leonurine and leonurine) interacted with Tyr’s active site, according to docking simulations. 10-methoxy-leonurine was totally fitted into Tyr’s active site through hydrophobic bonds between the aromatic ring and the amino acids, while the ketonic ester moiety interacted with His244 at a distance of 3.5 Å. In contrast, leonurine’s aromatic ring formed hydrogen bonds with His85, Cys83, and Ala323, whereas the leonurine guanidine moiety was out of the active center due to steric factors.

All the natural secondary metabolites which have been valued for their anti-Tyr activity are represented in ANTIAGE-DB at the following link: https://bio-hpc.ucam.edu/anti-age-db/web/Info/Info.php#TableS6 (accessed on 27 September 2022), as well as in Supplementary Material Table S6. A description regarding the importance of present –OH groups in the flavonoids’ structure is also analysed in Supplementary Material, Section 3.2.

## 3. Software Development: The Anti-Age Database

### 3.1. The Anti-Age Database (ANTIAGE-DB)

Based on the information reported above and accumulated from the international literature for the period 1965–2000 we developed a database (https://bio-hpc.ucam.edu/anti-age-db (accessed on 27 September 2022)) composed of the natural products that have been reported to interact with the three targeted enzymes (Human Neutrophil Elastase, Hyaluronidase, and Tyrosinase). Description of their interaction profiles with each of these enzyme targets is delivered. This inspired us to develop also additional tools that can be used to explore this chemical space in two ways: (a) a structure-based approach and (b) a ligand-based approach. Through the ligand-based approach, the studied natural compound is screened against known inhibitors towards each enzyme. This ligand-based similarity approach of the targeted compound (either natural, seminynthetic, or even synthetic), with known inhibitory potency towards each of the studied enzymes, could reveal unprecedented scaffold similarity that could imply common inhibitory activity. Since two isosteric molecules (retain similar volumes and shapes), this, could also be considered bioisosteres given the probability to recognize a similar or the same protein environment. This bioisosterism in chemical molecules could provide the predictive capacity power to determine for a given compound potent inhibitory potency towards Human Neutrophil Elastase, Hyaluronidase, and Tyrosinase. To provide flexibility to the user, the specific tool can be used through the submission of SMILES of the desired compound or via drawing the structure of the compounds in an appropriate drawing window and submitting it for the performance of similarity metrics. Moreover, a tool that can directly both add the name of the compound and search for a compound is offered. This database was created and curated manually for compounds with known inhibitory properties towards Human Neutrophil Elastase, Hyaluronidase, and Tyrosinase.

### 3.2. Methods

#### 3.2.1. Ligand Based Virtual Screening (LBVS)

ANTIAGE-DB uses a ligand based virtual screening (LBVS) approach based on two methods. A global three-dimensional shape similarity search is performed by means of WEGA [181] by pure shape scoring function comparison where all parameters are set as default. Additionally, a hybrid similarity search method based on 3D global shape and pharmacophore fitting can be performed using a SHAFTS tool [182]. All ligands in the database were prepared for LBVS using a molconvert tool (ChemAxon, Budapest, Hungary) for conformer generation. Molecular parameters for the ligands in the database were calculated by removing salts and neutralizing their protonation state, computing partial charges from the MMFF94 force field, adding hydrogen atoms, and minimizing energies (default parameters) [183] using AmberTools [184]. A maximum of 100 conformations for every compound stored in ANTIAGE-DB were generated with Omega [185], keeping default parameters. For 12 compounds of the database, stereoisomery and strict atom typing were set to false so conformations could be generated. For 9 compounds, conformer generation failed due to either containing metal or Boron atoms not supported by MMFF94 or being peptides not fitted for this ligand-based approach. Conformation generation for compounds submitted by ANTIAGE-DB users are generated by means of Cyndi [186], using default parameters and MMFF94 force field.

#### 3.2.2. Structure Based Virtual Screening (SBVS)

Through the ANTIAGE-DB tool, a user can submit structure-based calculations for an input compound in order to perform in silico docking to the following selected ageing protein targets: (a) elastase (PDB:1B0F) [187], (b) hyaluronidase (PDB 2PE4) [68], and (c) tyrosinase (PDB: 3NM8) [188]. Docking calculations are carried out using Autodock Vina, setting a grid of 25 Å^3^ centered in the binding site of every protein target and default parameters. Protein targets and ligands are set for docking with AutoDock tools as in Forli [189].

### 3.3. Anti-Age Database Design

This section explains the ANTIAGE-DB database structure and its content, the steps to access the database, and how the calculations are submitted to the web server.

#### 3.3.1. Database Structure and Content

ANTIAGE-DB database is composed of two interrelated blocks, namely Compounds and Experiments, which are represented by a set of tables. The former stores information about cosmetic and therapeutic compounds: name, structure, SMILES (Simplified Molecular-Input Line-Entry System), etc. The Experiments’ related tables store information about docking experiments and structure similarity comparison requests.

**Figure 4:**
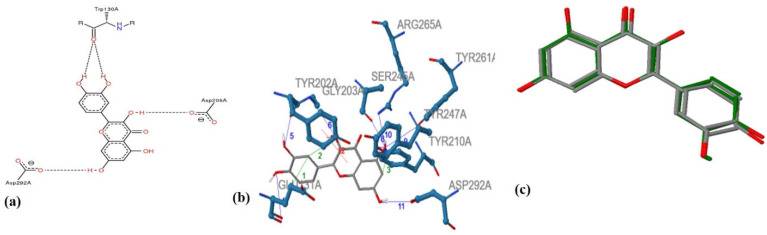
Representation of interactions formed between Hyaluronidase (PDB ID: 2PE4)- quercetin: (**a**) Hydrogen bonds between quercetin and Trp130A, Asp206A and Asp292A, (**b**) Hydrogen bonds, pi-stacking and Hydrophobic Interactions between Hyaluronidase-quercetin, (**c**) Ligand similarity result between quercetin (green) and naringenin (grey), as reported by ANTIAGE-DB.

The docking and similarity comparison experiments exploit the molecular data included in the Compounds tables (see Figure 4a–c).

#### 3.3.2. Submitting Calculations

One of the most important aspects of ANTIAGE-DB is the representation of information about cosmetic and therapeutic compounds and the ability to actively interact with the user since it offers two fundamental tools of virtual screening, which can be used for the prediction of biological activity. Running a prediction using ANTIAGE-DB is a very trivial and straightforward procedure (Figures S5 and S6). On the Calculations page, the user has to submit either the SMILES code of the desired compound or the structure of the molecule using the DRAW MOLECULE option (Figure S5, 2nd arrow). If so, another window opens, which can be used for the design of the molecule. Next, the user inserts his/her e-mail (Figure S5, 3rd arrow) and presses the SUBMIT DATA button (Figure S5, 5th arrow). As a security check, the user will have to complete a CAPTCHA form to ensure the user is a human (Figure S5, 4th arrow).

ANTIAGE-DB features 2 different types of calculations: docking experiments and structure similarity comparison (Figure S5, 1st arrow). In the first case, the interaction between the desired compound and the three protein targets will be calculated, whereas on the second case the desired compound will be compared with other known cosmetic and therapeutic compounds, and the global shape similarity between them will be measured.

After the chosen experiment is finished, the user will receive an e-mail containing an access code for that specific experiment. By inserting his/her e-mail address and the received code in the RESULTS page (Figure S6b), the user will be able to access the results. The user will see a table-formatted summary of the results, but he/she can obtain more detailed information by clicking on the ADDITIONAL INFO button (Figure S6c).

### 3.4. Implementation and Web Access

ANTIAGE-DB system has been deployed on an Apache Tomcat Web server running on an Ubuntu 14.05.5 LTS Linux Server. The user interfaces have followed a friendly and interactive web design obtained by means of the combination of several front-end technologies such as JavaScript [190], JQuery [191], PHP, HTML, and CSS. Data managed by ANTIAGE-DB is stored in a relational database through the MySQL database management system [192]. The content of this database will be continuously maintained since newly discovered compounds will be added. All docking and shape similarity queries are sent to a remote computer cluster where a SLURM [192] based queue manager distributes the jobs. Results are sent to the user by an e-mail with a link to a web page where those are displayed in HTML format.

The system Is available at https://bio-hpc.ucam.edu/anti-age-db (accessed on 27 September 2022) where registration is not necessary. A set of detailed tutorials, examples, frequently asked questions, and related publications, among others, are offered.

There are several ways to perform queries in ANTIAGE-DB: searching by name or SMILES or by a request of a docking study. In the first case, a detailed view of compounds can be obtained online in real time. In the second case, for docking studies, a detailed report with the prediction docking results are sent to the user by email once simulations are completed. ANTIAGE-DB server will send an email to the user with a link where the results will be displayed. This can take from several hours to two or three days depending on the query compound and cluster occupation.

## 4. Validation of ANTIAGE-DB

Molecular docking experiments were performed via Maestro software for validating the results simulated by ANTIAGE-DB. For the molecular docking studies, structures of the selected phytochemicals (quercetin, betulinic acid, caffeic acid) were built via Maestro 10.2 utility 1, prepared with LigPrep, ionized at pH 7.0, minimized using the OPLS3 force field [193], while ConfGen in the thorough mode was used to generate possible bioactive conformations [194]. The crystal structures of Hyaluronidase (PDB ID: 2PE4), Human Neutrophil Elastase (PDB ID: 1BF0), and Tyrosinase (PDB ID: 3NM8) were prepared using the Protein Preparation Wizard utility [195]. The receptor grid was generated centroid the catalytic site of each enzyme. The GlideXP algorithm was used for ligands’ docking [196]. All the created poses were minimized post-docking (Table S7). In order to reassure our predictions, we selected a phytochemical compound described in recent literature [50] as potent elastase inhibitor, and we compared the interactions via ANTIAGE-DB and Maestro software (Table S8).

## 5. Conclusions

Oxidative stress caused by UV irradiation is highly connected to skin inflammatory responses. Along with the produced ROS in skin cells, early aging is being developed as vital constituents of skin tissues such as collagen, elastin, hyaluronic acid, etc are being continuously degraded. Thus, the discovery of novel anti-aging products is of immense importance. Along these lines, natural products have served as a constant reservoir for the discovery of new anti-aging agents. Indeed, several studies on the inhibitory activity of various natural compounds, as well as their natural sources, against Human Neutrophil Elastase, Hyaluronidase, and Tyrosinase have unveiled numerous natural product inhibitors which can be effectively used in skin care cosmetics. To further assist the discovery process of potent anti-aging compounds, we developed the ANTIAGE-DB where for a first time we integrated the inhibitory properties for natural compounds against HNE, Hyal, and Tyr as collected for the period 1965–2020. This database has been developed to allow a flexible and easy interrogation of the inhibition properties of natural products as also to perform in real time in silico prediction of compounds that can interact with the studied enzymes. The server provides information on their atomic level interactions with the three enzymes as also the calculated binding affinity towards each enzyme. ANTIAGE-DB is freely accessible and is directed to scientists of various fields. The results can be obtained in less than 2 h and the user can acquire information simultaneously for the ligand and its interaction with the three enzymes. This information has been accumulated for the first time, and the associated tool we developed to explore and exploit the chemical space of natural products against HNE, Hyal, and Tyr could pave the way towards the development of novel inhibitors with antiaging potential.

## Supplementary Materials

The following supporting information can be downloaded at: https://www.mdpi.com/article/10.3390/antiox11112268/s1, Table S1: Plants, extracts and isolated compounds that have been studied for their inhibitory properties towards HNE; Table S2: Studied natural secondary metabolites for their inhibitory activity towards HNE; Table S3: Plants, extracts and isolated compounds that have been studied for their inhibitory properties towards Hyaluronidase; Table S4: Studied natural secondary metabolites for their inhibitory potency towards Hyaluronidase; Table S5: Plants, extracts and isolated compounds that have been studied for their inhibitory properties towards Tyrosinase; Table S6: Studied natural secondary metabolites for their inhibitory properties towards Tyrosinase; Table S7: Illustration of the results given with the two softwares (NC-DB and Maestro) (a) Elastase with caffeic acid, (b) Hyaluronidase with quercetin and (c) Tyrosinase with betulinic acid; Table S8: Illustration of the results given with the two softwares (NC-DB and Maestro) for the compound Auricoulasin, potent Elastase inhibitor; Figure S1: (a) Structure of Human Neutrophil Elastase (PDB ID: 1B0F) and its catalytic triad: Ser195-His57-Asp102 (b) Structure of Human Neutrophil Elastase (PDB ID: 1B0F) and its catalytic Ser195 which forms hydrogen bonds with Gly193, creating the “oxyanion hole”; Figure S2: (a) Illustration of the Human Neutrophil Elastase’s catalytic triad (PDB ID:1B0F) (b) Illustration of the main chain interaction between a peptide (substrate or inhibitor) with Human Neutrophil Elastase’s subsite pockets (SI-S5); Figure S3: (a) Structure of Hyaluronidase (PDB ID: 2PE4), indicating the catalytic sited amino acids, (b) Illustration of Hyaluronidase’s catalytic sited amino acids (PDB ID:2PE4); Figure S4: (a) Structure of Tyrosinase (PDB ID: 3NM8), indicating the catalytic sited amino acids, (b) Illustration of Tyrosinase’s catalytic sited amino acids (PDB ID: 3NM8); Figure S5: Representation of the first step of the determination of calculations in ANTIAGE-DB, by inserting the respective SMILES of the studied compound; Figure S6: Detailed representation of the whole procedure for the submission of calculations in the ANTIAGE-DB. The respective arrows are explained in Section 2.3.

## References

[1] Li X. (2015). Anti-aging cosmetics and its efficacy assessment methods. IOP Conf. Ser. Mater. Sci. Eng..

[2] Heck D.E., Vetrano A.M., Mariano T.M., Laskin J.D. (2003). UVB light stimulates production of reactive oxygen species—Unexpected role for catalase. J. Biol. Chem..

[3] Rinnerthaler M., Bischof J., Streubel M.K., Trost A., Richter K. (2015). Oxidative stress in aging human skin. Biomolecules.

[4] Necas J., Bartosikova L., Brauner P., Kolar J. (2008). Hyaluronic acid (hyaluronan): A review. Vet. Med..

[5] Chang T.S. (2009). An updated review of tyrosinase inhibitors. Int. J. Mol. Sci..

[6] Mainka M., Czerwińska M.E., Osińska E., Ziaja M., Bazylko A. (2021). Screening of antioxidative properties and inhibition of inflammation-linked enzymes by aqueous and ethanolic extracts of plants traditionally used in wound healing in Poland. Antioxidants.

[7] Kim K., Lee Y.S., Kim N., Choi H.D., Lim K.M. (2022). 5G Electromagnetic Radiation Attenuates Skin Melanogenesis In Vitro by Suppressing ROS Generation. Antioxidants.

[8] Myung C.H., Kim K., Park J.I., Lee J.E., Lee J.A., Hong S.C., Lim K.-M., Hwang J.S. (2020). 16-Kauren-2-beta-18, 19-triol inhibits melanosome transport in melanocytes by down-regulation of melanophilin expression. J. Dermatol. Sci..

[9] Southam C.M. (1943). Effects of extracts of western red-cedar heartwood on certain wood-decaying fungi in culture. Phytophathology.

[10] Calabrese E.J., Dhawan G., Kapoor R., Iavicoli I., Calabrese V. (2015). What is hormesis and its relevance to healthy aging and longevity?. Biogerontology.

[11] Calabrese E.J. (2014). Hormesis: A fundamental concept in biology. Microb. Cell.

[12] Rattan S.I.S. (2004). Aging, anti-aging, and hormesis. Mech. Ageing Dev..

[13] Minois N. (2001). Applying hormesis in aging research and therapy: A commentary. Hum. Exp. Toxicol..

[14] Heiflick L. (2000). The future of ageing. Nature.

[15] Calabrese E.J. (2013). Hormetic Mechanisms. Crit. Rev. Toxicol..

[16] Bieth J.G. (2001). The elastases. J. Soc. Biol..

[17] Takahashi H., Nukiwa T., Yoshimura K., Quick C.D., States D.J., Holmes M.D., Whang-Peng J., Knutsen T., Crystal R.G. (1988). Structure of the human neutrophil elastase gene. J. Biol. Chem..

[18] Melzig M.F., Löser B., Ciesielski S. (2001). Inhibition of neutrophil elastase activity by phenolic compounds from plants. Pharmazie.

[19] Siedle B., Cisielski S., Murillo R., Lo B., Castro V., Klaas C.A., Hucke O., Labahn A., Melzig M.F., Merfort I. (2002). Sesquiterpene Lactones as Inhibitors of Human Neutrophil Elastase. Bioorg. Med. Chem..

[20] Kim Y.-J., Uyama H., Kobayashi S. (2004). Inhibition effects of (+)-catechin-aldehyde polycondensates on proteinases causing proteolytic degradation of extracellular matrix. Biochem. Biophys. Res. Commun..

[21] Thring T.S., Hili P., Naughton D.P. (2009). Anti-collagenase, anti-elastase and anti-oxidant activities of extracts from 21 plants. BMC Complement. Altern. Med..

[22] Belaaouaj A., Kim K.S., Shapiro S.D. (2000). Degradation of outer membrane protein A in Escherichia coli killing by neutrophil elastase. Science.

[23] Ying Q.L., Rinehart A.R., Simon S.R., Cheronis J.C. (1991). Inhibition of human leucocyte elastase by ursolic acid. Evidence for a binding site for pentacyclic triterpenes. Biochem. J..

[24] Bode W., Edgar Meyer J.C.P. (1989). Perspectives in Biochemistry. Human Leukocyte and PorcinePancreatic Elastase: X-ray Crystal Structures, Mechanism, Substrate Specificity. Biochemistry.

[25] Feng L., Liu X., Zhu W., Guo F., Wu Y., Wang R., Chen K., Huang C., Li Y. (2013). Inhibition of human neutrophil elastase by pentacyclic triterpenes. PLoS ONE.

[26] Tamada T., Kinoshita T., Kurihara K., Adachi M., Ohhara T., Imai K., Kuroki R., Tada T. (2009). Combined high-resolution neutron and X-ray analysis of inhibited elastase confirms the active-site oxyanion hole but rules against a low-barrier hydrogen bond. J. Am. Chem. Soc..

[27] Hess G.P., McConn J., Ku E., McConkey G. (1970). Studies of the activity of chymotrypsin. Philos. Trans. R. Soc. Lond. B Biol. Sci..

[28] Wittenauer J., Mäckle S., Sußmann D., Schweiggert-Weisz U., Carle R. (2015). Inhibitory effects of polyphenols from grape pomace extract on collagenase and elastase activity. Fitoterapia.

[29] Alasbahi R., Matthias M. (2008). The in vitro inhibition of human neutrophil elastase activity by some Yemeni medicinal plants. Planta Med..

[30] Lee K.K., Kim J.H., Cho J.J., Choi J.D. (1999). Inhibitory Effects of 150 Plant Extracts on Elastase Activity, and Their Anti-inflammatory Effects. Int. J. Cosmet. Sci..

[31] Pinelo M., Rubilar M., Jerez M., Sineiro J., Núñez M.J. (2005). Effect of solvent, temperature, and solvent-to-solid ratio on the total phenolic content and antiradical activity of extracts from different components of grape pomace. J. Agric. Food Chem..

[32] Chiang H.-M., Lin T.-J., Chiu C.-Y., Chang C.-W., Hsu K.-C., Fan P.-C., Wen K.-C. (2011). Coffea arabica extract and its constituents prevent photoaging by suppressing MMPs expression and MAP kinase pathway. Food Chem. Toxicol..

[33] Kammerer D., Claus A., Carle R., Schieber A. (2004). Polyphenol Screening of Pomace from Red and White Grape Varieties (*Vitis vinifera* L.) by HPLC-DAD-MS/MS. J. Agric. Food Chem..

[34] Hubert J., Angelis A., Aligiannis N., Rosalia M., Abedini A., Bakiri A., Reynaud R., Nuzillard J.M., Gangloff S.C., Skaltsounis A.L. (2016). In vitro dermo-cosmetic evaluation of bark extracts from common temperate trees. Planta Med..

[35] Sumantran V.N., Kulkarni A.A., Harsulkar A., Wele A., Koppikar S.J., Chandwaskar R., Gaire V., Dalvi M., Wagh U.V. (2007). Hyaluronidase and collagenase inhibitory activities of the herbal formulation Triphala guggulu. J. Biosci..

[36] Yang X.W., Li S.M., Shen Y.H., Zhang W.D. (2008). Phytochemical and biological studies of Abies species. Chem. Biodivers..

[37] Kostova I., Iossifova T. (2007). Chemical components of Fraxinus species. Fitoterapia.

[38] Nahrstedt A., Schmidt M., Jäggi R., Metz J., Khayyal M.T. (2007). Willow bark extract: The contribution of polyphenols to the overall effect. Wien. Med. Wochenschr..

[39] Tyagi S.C., Simon S.R. (1990). Inhibitors directed to binding domains in neutrophil elastase. Biochemistry.

[40] Siedle B., Hrenn A., Merfort I. (2007). Natural compounds as inhibitors of human neutrophil elastase. Planta Med..

[41] Löser B., Kruse S.O., Melzig M.F., Nahrstedt A. (2000). Inhibition of neutrophil elastase activity by cinnamic acid derivatives from Cimicifuga racemosa. Planta Med..

[42] Melzig M.F., Löser B., Lobitz G.O., Tamayo-Castillo G., Merfort I. (1999). Inhibition of granulocyte elastase activity by caffeic acid derivatives. Pharmazie.

[43] Hamburger M., Riese U., Graf H., Melzig M.F., Ciesielski S., Baumann D., Dittmann K., Wegner C. (2002). Constituents in Evening Primrose Oil with Radical Scavenging, Cyclooxygenase, and Neutrophil Elastase Inhibitory Activities. J. Agric. Food Chem..

[44] Xing X., Yang X., Cao Y. (2016). Study of Ellagic Acid as a Natural Elastase Inhibitor by Spectroscopic Methods. J. Appl. Spectrosc..

[45] Rennert B., Melzig M.F. (2002). Free fatty acids inhibit the activity of Clostridium histolyticum collagenase and human neutrophil elastase. Planta Med..

[46] Bizot-Foulon Godeau G., Guessous F., Lati E., Rousset G., Roch-Arveillier M., Hornebeck W.V. (1995). Inhibition of human neutrophil elastase by wheat ceramides. Int. J. Cosmet. Sci..

[47] Sim G.S., Lee B.C., Cho H.S., Lee J.W., Kim J.H., Lee D.H., Kim J.-H., Pyo H.B., Moon D.C., Oh K.W. (2007). Structure activity relationship of antioxidative property of flavonoids and inhibitory effect on matrix metalloproteinase activity in UVA-irradiated human dermal fibroblast. Arch. Pharmacol. Res..

[48] Krenn L., Wollenweber E., Steyrleuthner K., Görick C., Melzig M.F. (2009). Contribution of methylated exudate flavonoids to the anti-inflammatory activity of Grindelia robusta. Fitoterapia.

[49] Tan X., Song Y.H., Park C., Lee K.W., Kim J.Y., Kim D.W., Kim K.D., Lee K.W., Curtis-Long M.J., Park K.H. (2016). Highly potent tyrosinase inhibitor, neorauflavane from Campylotropis hirtella and inhibitory mechanism with molecular docking. Bioorgan. Med. Chem..

[50] Kim J.Y., Wang Y., Uddin Z., Song Y.H., Li Z.P., Jenis J., Park K.H. (2018). Competitive neutrophil elastase inhibitory isoflavones from the roots of Flemingia philippinensis. Bioorg. Chem..

[51] Sivamani P., Singaravelu G., Thiagarajan V., Jayalakshmi T., Kumar G.R. (2012). Comparative molecular docking analysis of essential oil constituents as elastase inhibitors. Bioinformation.

[52] Calabrese E.J., Mattson M.P., Calabrese V. (2010). Resveratrol commonly displays hormesis: Occurrence and biomedical significance. Hum. Exp. Toxicol..

[53] Grubauer G., Elias P.M., Feingold K.R. (1989). Transepidermal water loss: The signal for recovery of barrier structure and function. J. Lipid Res..

[54] Nakajima K., Powers J.C., Ashe B.M., Zimmerman M. (1979). Mapping the extended substrate binding site of cathepsin G and human leukocyte elastase. Studies with peptide substrates related to the alpha 1-protease inhibitor reactive site. J. Biol. Chem..

[55] Baici A., Diczházi C., Neszmélyi A., Móczár E., Hornebeck W. (1993). Inhibition of the human leukocyte endopeptidases elastase and cathepsin G and of porcine pancreatic elastase by N-oleoyl derivatives of heparin. Biochem. Pharmacol..

[56] Fujino Y., Ohnishi M. (1976). Constituents of ceramide and ceramide monohexoside in rice bran. Chem. Phys. Lipids.

[57] Fujino Y., Ohnishi M. (1983). Sphingolipids in wheat grain. J. Cereal Sci..

[58] Feisst C., Franke L., Appendino G., Werz O. (2005). Identification of molecular targets of the oligomeric nonprenylated acylphloroglucinols from Myrtus communis and their implication as anti-inflammatory compounds. J. Pharmacol. Exp. Ther..

[59] Feisst C., Werz O. (2004). Suppression of receptor-mediated Ca^2+^ mobilization and functional leukocyte responses by hyperforin. Biochem. Pharmacol..

[60] Załuski D., Cieśla Ł., Janeczko Z. (2015). Chapter 7—The Structure—Activity Relationships of Plant Secondary Metabolites with Antimicrobial, Free Radical Scavenging and Inhibitory Activity toward Selected Enzymes. Stud. Nat. Prod. Chem..

[61] Marković-Housley Z., Miglierini G., Soldatova L., Rizkallah P.J., Müller U., Schirmer T. (2000). Crystal structure of hyaluronidase, a major allergen of bee venom. Structure.

[62] Laurent T.C., Fraser J.R. (1992). Hyaluronan. FASEB J..

[63] Meyer K. (1971). Hyaluronidases. The Enzymes.

[64] Stern R. (2003). Devising a pathway for hyaluronan catabolism: Are we there yet?. Glycobiology.

[65] Orlando Z., Lengers I., Melzig M.F., Buschauer A., Hensel A., Jose J. (2015). Autodisplay of human hyaluronidase Hyal-1 on Escherichia coli and identification of plant-derived enzyme inhibitors. Molecules.

[66] Jedrzejas M.J., Stern R. (2005). Structures of vertebrate hyaluronidases and their unique enzymatic mechanism of hydrolysis. Proteins.

[67] Csoka A.B., Frost G.I., Stern R. (2001). The six hyaluronidase-like genes in the human and mouse genomes. Matrix Biol..

[68] Chao K.L., Muthukumar L., Herzberg O. (2007). Structure of human hyaluronidase-1, a hyaluronan hydrolyzing enzyme involved in tumor growth and angiogenesis. Biochemistry.

[69] Lokeshwar V.B., Rubinowicz D., Schroeder G.L., Forgacs E., Minna J.D., Block N.L., Nadji M., Lokeshwar B.L. (2001). Stromal and epithelial expression of tumor markers hyaluronic acid and HYAL1 hyaluronidase in prostate cancer. J. Biol. Chem..

[70] Lokeshwar V.B., Obek C., Pham H.T., Wei D., Young M.J., Duncan R.C., Soloway M.S., Block N.L. (2000). Urinary hyaluronic acid and hyaluronidase: Markers for bladder cancer detection and evaluation of grade. J. Urol..

[71] Tan J.X., Wang X.Y., Li H.Y., Su X.L., Wang L., Ran L., Zheng K., Ren G.S. (2011). HYAL1 overexpression is correlated with the malignant behavior of human breast cancer. Int. J. Cancer.

[72] Mio K., Stern R. (2002). Inhibitors of the hyaluronidases. Matrix Biol..

[73] Jentsch H., Pomowski R., Kundt G., Göcke R. (2003). Treatment of gingivitis with hyaluronan. J. Clin. Periodontol..

[74] Stern R., Jedrzejas M.J. (2006). Hyaluronidases: Their genomics, structures, and mechanisms of action. Chem. Rev..

[75] Hunnicutt G.R., Primakoff P., Myles D.G. (1996). Sperm surface protein PH-20 is bifunctional: One activity is a hyaluronidase and a second, distinct activity is required in secondary sperm-zona binding. Biol. Reprod..

[76] Fayad S., Nehmé R., Tannoury M., Lesellier E., Pichon C., Morin P. (2017). Macroalga Padina pavonica water extracts obtained by pressurized liquid extraction and microwave-assisted extraction inhibit hyaluronidase activity as shown by capillary electrophoresis. J. Chromatogr. A.

[77] Bralley E., Greenspan P., Hargrove J.L., Hartle D.K. (2008). Inhibition of hyaluronidase activity by select sorghum brans. J. Med. Food.

[78] Pessini A.C., Takao T.T., Cavalheiro E.C., Vichnewski W., Sampaio S.V., Giglio J.R., Arantes E.C. (2001). A hyaluronidase from Tityus serrulatus scorpion venom: Isolation, characterization and inhibition by flavonoids. Toxicon.

[79] Pujiarti R., Ohtani Y., Ichura H. (2012). Antioxidant, Anti-Hyaluronidase and Antifungal Activities of Melaleuca leucadendron Linn. Leaf Oils. J. Wood Sci..

[80] Załuski D., Olech M., Kuźniewski R., Verpoorte R., Nowak R., Smolarz H.D. (2017). LC-ESI-MS/MS profiling of phenolics from Eleutherococcus spp. inflorescences, structure-activity relationship as antioxidants, inhibitors of hyaluronidase and acetylcholinesterase. Saudi Pharm. J..

[81] Moon S.H., Lee J.H., Kim K.T., Park Y.S., Nah S.Y., Ahn D.U., Paik H.D. (2009). Antimicrobial Effect of 7-O-Butylnaringenin, a Novel Flavonoid, and Various Natural Flavonoids against Helicobacter Pylori Strains. Food Sci. Biotechnol..

[82] Hertel W., Peschel G., Ozegowski J.-H., Müller P.-J. (2006). Inhibitory Effects of Triterpenes and Flavonoids on the Enzymatic Activity of Hyaluronic Acid-Splitting Enzymes. Arch. Pharm..

[83] Hisao K., Matsumoto H., Satoh T. (1992). Inhibitory Effects of Some Natural Products on the Activation of Hyaluronidase and Their Antiallergic Actions. Chem. Pharm. Bull..

[84] Zeng H.J., Yang R., You J., Qu L.B., Sun Y.J. (2016). Spectroscopic and Docking Studies on the Binding of Liquiritigenin with Hyaluronidase for Antiallergic Mechanism. Scientifica.

[85] Barla F., Higashijima H., Funai S., Sugimoto K., Harada N., Yamaji R., Fujita T., Nakano Y., Inui H. (2009). Inhibitive effects of alkyl gallates on hyaluronidase and collagenase. Biosci. Biotechnol. Biochem..

[86] Lee S.Y., Baek N., Nam T.G. (2016). Natural, semisynthetic and synthetic tyrosinase inhibitors. J. Enzym. Inhib. Med. Chem..

[87] Jackman M.P., Hajnal A., Lerch K. (1991). Albino mutants of Streptomyces glaucescens tyrosinase. Biochem. J..

[88] van Gelder C.W., Flurkey W.H., Wichers H.J. (1997). Sequence and structural features of plant and fungal tyrosinases. Phytochemistry.

[89] Himmelwright R.S., Eickman N.C., Lu Bien C.D., Lerch K., Solomon E. (1980). Chemical and Spectroscopic Studies of the Binuclear Copper Active Site of Neurospora Tyrosinase: Comparison to Hemocyanins. J. Am. Chem. Soc..

[90] Magnus K.A., Hazes B., Ton-That H., Bonaventura C., Bonaventura J., Hol W.G.J. (1994). Crystallographic Analysis of Oxygenated and Deoxygenated States of Arthropod Hemocyanin Shows Unusual Differences. Genet. Proteins Struct. Funct..

[91] Volbeda A., Hol W.G. (1989). Crystal Structure of Hexameric Haemocyanin from Panulirus Interruptus Refined at 3.2 A Resolution. J. Mol. Biol..

[92] Wang C., Yan S., Huang R., Feng S., Fu B., Weng X., Zhou X. (2013). A turn-on fluorescent probe for detection of tyrosinase activity. Analyst.

[93] Kubo I., Kinst-Hori I., Chaudhuri S.K., Kubo Y., Sánchez Y., Ogura T. (2000). Flavonols from Heterotheca inuloides: Tyrosinase inhibitory activity and structural criteria. Bioorg. Med. Chem..

[94] Kubo I., Kinst-Hori I., Yokokawa Y. (1994). Tyrosinase inhibitors from Anacardium occidentale fruits. J. Nat. Prod..

[95] Chen Q.-X., Kubo I. (2002). Kinetics of Mushroom Tyrosinase Inhibition by Quercetin. J. Agric. Food Chem..

[96] Kubo I., Kinst-Hori I., Kubo Y., Yamagiwa Y., Kamikawa T., Haraguchi H. (2000). Molecular design of antibrowning agents. J. Agric. Food Chem..

[97] Kubo I., Yokokawa Y., Kinst-Hori I. (1995). Tyrosinase inhibitors from Bolivian medicinal plants. J. Nat. Prod..

[98] Badria F.A., elGayyar M.A. (2001). A new type of tyrosinase inhibitors from natural products as potential treatments for hyperpigmentation. Boll. Chim. Farm..

[99] Xie L.-P., Chen Q.-X., Huang H., Wang H.-Z., Zhang R.-Q. (2003). Inhibitory effects of some flavonoids on the activity of mushroom tyrosinase. Biochemistry..

[100] Kubo I., Chen Q.X., Kenichi N. (2003). Molecular Design of Antibrowning Agents: Antioxidative Tyrosinase Inhibitors. Food Chem..

[101] No J.K., Soung D.Y., Kim Y.J., Shim K.H., Jun Y.S., Rhee S.H., Yokozawa T., Chung H.Y. (1999). Inhibition of tyrosinase by green tea components. Life Sci..

[102] Kim J.H., Sapers G.M., Choi S.W. (1998). Identification of Tyrosinase Inhibitor from Galla Rhois. Food Sci. Biotechnol..

[103] Parvez S., Kang M., Chung H.-S., Bae H. (2007). Naturally occurring tyrosinase inhibitors: Mechanism and applications in skin health, cosmetics and agriculture industries. Phytother. Res..

[104] Menon S., Fleck R.W., Yong G., Strothkamp K.G. (1990). Benzoic acid inhibition of the alpha, beta, and gamma isozymes of Agaricus bisporus tyrosinase. Arch. Biochem. Biophys..

[105] Kermasha S., Goetghebeur M., Monfette A., Metche M., Rovel B. (1993). Inhibitory effects of cysteine and aromatic acids on tyrosinase activity. Phytochemistry.

[106] Jiménez M., Chazarra S., Escribano J., Cabanes J., García-Carmona F. (2001). Competitive Inhibition of Mushroom Tyrosinase by 4-Substituted Benzaldehydes. J. Agric. Food Chem..

[107] Wilcox D.E., Porras A.G., Hwang Y.T., Lerch K., Winkler M.E., Solomon E.I. (1985). Substrate analog binding to the coupled binuclear copper active site in tyrosinase. J. Am. Chem. Soc..

[108] Dilberger B., Weppler S., Eckert G.P. (2021). Phenolic acid metabolites of polyphenols act as inductors for hormesis in *C. elegans*. Mech. Ageing Dev..

[109] Kim Y.J. (2013). Rhamnetin attenuates melanogenesis by suppressing oxidative stress and pro-inflammatory mediators. Biol. Pharm. Bull..

[110] Stratford M.R.L., Ramsden C.A., Riley P.A. (2013). Mechanistic studies of the inactivation of tyrosinase by resorcinol. Bioorg. Med. Chem..

[111] Tang H., Cui F., Li H., Huang Q., Li Y. (2018). Understanding the inhibitory mechanism of tea polyphenols against tyrosinase using fluorescence spectroscopy, cyclic voltammetry, oximetry, and molecular simulations. RSC Adv..

[112] Liang Y.R., Kang S., Deng L., Xiang L.P., Zheng X.Q. (2014). Inhibitory effects of (-)-epigallocatechin-3-gallate on melanogenesis in ultraviolet A-induced B16 murine melanoma cell. Trop. J. Pharm. Res..

[113] Jin Y.J., Lin C.C., Lu T.M., Li J.H., Chen I.S., Kuo Y.H., Ko H.H. (2015). Chemical constituents derived from Artocarpus xanthocarpus as inhibitors of melanin biosynthesis. Phytochemistry.

[114] Arung E.T., Shimizu K., Kondo R. (2007). Structure-activity relationship of prenyl-substituted polyphenols from Artocarpus heterophyllus as inhibitors of melanin biosynthesis in cultured melanoma cells. Chem. Biodivers..

[115] Lin Y.P., Hsu F.L., Chen C.S., Chern J.W., Lee M.H. (2007). Constituents from the Formosan apple reduce tyrosinase activity in human epidermal melanocytes. Phytochemistry.

[116] Briganti S., Camera E., Picardo M. (2003). Chemical and instrumental approaches to treat hyperpigmentation. Pigment Cell Res..

[117] Khatib S., Nerya O., Musa R., Shmuel M., Tamir S., Vaya J. (2005). Chalcones as potent tyrosinase inhibitors: The importance of a 2,4-substituted resorcinol moiety. Bioorg. Med. Chem..

[118] Li N., Xue M.-H., Yao H., Zhu J.-J. (2005). Reagentless biosensor for phenolic compounds based on tyrosinase entrapped within gelatine film. Anal. Bioanal. Chem..

[119] Matoba Y., Kumagai T., Yamamoto A., Yoshitsu H., Sugiyama M. (2006). Crystallographic evidence that the dinuclear copper center of tyrosinase is flexible during catalysis. J. Biol. Chem..

[120] Zhang G., Guo X.H., Wang S.S., Li Y.Q., Li G.Z., Zhao W.J. (2017). Screening and identification of natural ligands of tyrosinase from: Pueraria lobata Ohwi by a combination of ultrafiltration and LC-MS. Anal. Methods.

[121] Placines C., Castañeda-Loaiza V., Rodrigues M.J., Pereira C.G., Stefanucci A., Mollica A., Zengin G., Llorent-Martínez E.J., Castilho P.C., Custódio L. (2020). Phenolic profile, toxicity, enzyme inhibition, in silico studies, and antioxidant properties of *Cakile maritima* scop. (brassicaceae) from Southern Portugal. Plants.

[122] Amer M., Metwalli M. (1998). Topical hydroquinone in the treatment of some hyperpigmentary disorders. Int. J. Dermatol..

[123] Draelos Z.D. (2007). Skin lightening preparations and the hydroquinone controversy. Dermatol. Ther..

[124] Parejo I., Viladomat F., Bastida J., Codina C. (2001). A single extraction step in the quantitative analysis of arbutin in bearberry (*Arctostaphylos uva-ursi*) leaves by high-performance liquid chromatography. Phytochem. Anal..

[125] Jin Y.H., Lee S.J., Chung M.H., Park J.H., Park Y.I., Cho T.H., Lee S.K. (1999). Aloesin and arbutin inhibit tyrosinase activity in a synergistic manner via a different action mechanism. Arch. Pharm. Res..

[126] Jones K., Hughes J., Hong M., Jia Q., Orndorff S. (2002). Modulation of melanogenesis by aloesin: A competitive inhibitor of tyrosinase. Pigment Cell Res..

[127] Wolverton S.E., Wu J.J., Wolverton S.E. (2019). Cosmetic therapy. Comprehensive Dermatologic Drug Therapy.

[128] Batovska D.I., Todorova I.T. (2010). Trends in utilization of the pharmacological potential of chalcones. Curr. Clin. Pharmacol..

[129] Kim S.J., Son K.H., Chang H.W., Kang S.S., Kim H.P. (2003). Tyrosinase inhibitory prenylated flavonoids from Sophora flavescens. Biol. Pharm. Bull..

[130] Hyun S.K., Lee W.-H., Jeong D.M., Kim Y., Choi J.S. (2008). Inhibitory effects of kurarinol, kuraridinol, and trifolirhizin from Sophora flavescens on tyrosinase and melanin synthesis. Biol. Pharm. Bull..

[131] Nerya O., Musa R., Khatib S., Tamir S., Vaya J. (2004). Chalcones as potent tyrosinase inhibitors: The effect of hydroxyl positions and numbers. Phytochemistry.

[132] Nguyen N.T., Nguyen M.H.K., Nguyen H.X., Bui N.K.N., Nguyen M.T.T. (2012). Tyrosinase inhibitors from the wood of Artocarpus heterophyllus. J. Nat. Prod..

[133] Wang Y., Curtis-Long M.J., Lee B.W., Yuk H.J., Kim D.W., Tan X.F., Park K.H. (2014). Inhibition of tyrosinase activity by polyphenol compounds from Flemingia philippinensis roots. Bioorgan. Med. Chem..

[134] Aggarwal B.B., Bhardwaj A., Aggarwal R.S., Seeram N.P., Shishodia S., Takada Y. (2004). Role of resveratrol in prevention and therapy of cancer: Preclinical and clinical studies. Anticancer Res..

[135] Shin N.-H., Ryu S.Y., Choi E.J., Kang S.-H., Chang I.-M., Min K.R., Kim Y. (1998). Oxyresveratrol as the Potent Inhibitor on Dopa Oxidase Activity of Mushroom Tyrosinase. Biochem. Biophys. Res. Commun..

[136] Shimizu K., Kondo R., Sakai K., Lee S.-H., Sato H. (1998). The Inhibitory Components from *Artocarpus incisus* on Melanin Biosynthesis. Planta Med..

[137] Likhitwitayawuid K., Sritularak B. (2001). A new dimeric stilbene with tyrosinase inhibitiory activity from Artocarpus gomezianus. J. Nat. Prod..

[138] Ohguchi K., Tanaka T., Ito T., Iinuma M., Matsumoto K., Akao Y., Nozawa Y. (2003). Inhibitory effects of resveratrol derivatives from dipterocarpaceae plants on tyrosinase activity. Biosci. Biotechnol. Biochem..

[139] Likhitwitayawuid K., Sornsute A., Sritularak B., Ploypradith P. (2006). Chemical transformations of oxyresveratrol (trans-2,4,3′,5′-tetrahydroxystilbene) into a potent tyrosinase inhibitor and a strong cytotoxic agent. Bioorgan. Med. Chem. Lett..

[140] Burdock G.A., Soni M.G., Carabin I.G. (2001). Evaluation of health aspects of kojic acid in food. Regul. Toxicol. Pharmacol..

[141] Bentley R. (2006). From miso, saké and shoyu to cosmetics: A century of science for kojic acid. Nat. Prod. Rep..

[142] Kim Y.-J.J., Uyama H. (2005). Tyrosinase inhibitors from natural and synthetic sources: Structure, inhibition mechanism and perspective for the future. Cell. Mol. Life Sci..

[143] Kahn V., Ben-Shalom N., Zakin V. (1997). Effect of Kojic Acid on the Oxidation of N-Acetyldopamine by Mushroom Tyrosinase. J. Agric. Food Chem..

[144] Kim H., Choi J., Cho J.K., Kim S.Y., Lee Y.-S. (2004). Solid-phase synthesis of kojic acid-tripeptides and their tyrosinase inhibitory activity, storage stability, and toxicity. Bioorgan. Med. Chem. Lett..

[145] Choi S., Lee S.-K., Kim J.-E., Chung M.-H., Park Y.-I. (2002). Aloesin inhibits hyperpigmentation induced by UV radiation. Clin. Exp. Dermatol..

[146] Masamoto Y., Ando H., Murata Y., Shimoishi Y., Tada M., Takahata K. (2003). Mushroom Tyrosinase Inhibitory Activity of Esculetin Isolated from Seeds of *Euphorbia lathyris* L. Biosci. Biotechnol. Biochem..

[147] Piao X.L., Baek S.H., Park M.K., Park J.H. (2004). Tyrosinase-inhibitory furanocoumarin from Angelica dahurica. Biol. Pharm. Bull..

[148] Ito S., Wakamatsu K. (2015). A convenient screening method to differentiate phenolic skin whitening tyrosinase inhibitors from leukoderma-inducing phenols. J. Dermatol. Sci..

[149] Lim J.Y., Ishiguro K., Kubo I. (1999). Tyrosinase inhibitory p-coumaric acid from ginseng leaves. Phytother. Res..

[150] Maghsoudi S., Adibi H., Hamzeh M., Ashrafi-Kooshk M.R., Rezaei-Tavirani M., Khodarahmi R. (2013). Kinetic of Mushroom Tyrosinase Inhibition by Benzaldehyde Derivatives. J. Rep. Pharm. Sci. Med. Sci..

[151] Duckworth H.W., Coleman J.E. (1970). Physicochemical and kinetic properties of mushroom tyrosinase. J. Biol. Chem..

[152] Kubo I., Chen Q.-X., Nihei K.-I., Calderón J.S., Céspedes C.L. (2003). Tyrosinase inhibition kinetics of anisic acid. Z. Naturforsch. C.

[153] Ha T.J., Tamura S., Kubo I. (2005). Effects of mushroom tyrosinase on anisaldehyde. J. Agric. Food Chem..

[154] Shi Y., Chen Q., Wang Q., Song K., Qiu L. (2005). Inhibitory effects of cinnamic acid and its derivatives on the diphenolase activity of mushroom (*Agaricus bisporus*) tyrosinase. Food Chem..

[155] Lee H.-S. (2002). Tyrosinase inhibitors of Pulsatilla cernua root-derived materials. J. Agric. Food Chem..

[156] Miyazawa M., Oshima T., Koshio K., Itsuzaki Y., Anzai J. (2003). Tyrosinase inhibitor from black rice bran. J. Agric. Food Chem..

[157] Isao K., Kinst-Hori I. (1998). Tyrosinase Inhibitors from Cumin. J. Agric. Food Chem..

[158] Conrad J.S., Dawso S.R., Hubbard E.R., Meyers T.E., Strothkamp K.G. (1994). Inhibitor binding to the binuclear active site of tyrosinase: Temperature, pH, and solvent deuterium isotope effects. Biochemistry.

[159] Zhu J., Yan G., Xu Z., Hu X., Wang G., Wang T., Zhu W., Hou A., Wang H. (2015). Inhibitory Effects of (2′R)-2′,3′-dihydro-2′-(1-hydroxy-1-methylethyl)-2,6′-bibenzofuran-6,4′-diol on Mushroom Tyrosinase and Melanogenesis in B16-F10 Melanoma Cells. Phytother. Res..

[160] Kim Y.M., Yun J., Lee C.-K., Lee H., Min K.R., Kim Y. (2002). Oxyresveratrol and Hydroxystilbene Compounds. J. Biol. Chem..

[161] Chen J.S., Wei C.I., Marshall M.R. (1991). Inhibition mechanism of kojic acid on polyphenol oxidase. J. Agric. Food Chem..

[162] Rho H.S., Goh M., Lee J.K., Ahn S.M., Yeon J.H., Yoo D.S., Kim D.H., Kim H.G., Cho J.Y. (2011). Ester Derivatives of Kojic Acid and Polyphenols Containing Adamantane Moiety with Tyrosinase Inhibitory and Anti-Inflammatory Properties. Bull. Korean Chem. Soc..

[163] Jeon H.J., Noda M., Maruyama M., Matoba Y., Kumagai T., Sugiyama M. (2006). Identification and Kinetic Study of Tyrosinase Inhibitors Found in Sake Lees. J. Agric. Food Chem..

[164] Magid A.A., Voutguenne-Nazabadioko L., Bontemps G., Litaudon M., Lavaud C. (2008). Tyrosinase Inhibitors and Sesquiterpene Diglycosides from Guioa Villosa. Planta Med..

[165] Masuda T., Odaka Y., Ogawa N., Nakamoto K., Kuninaga H. (2008). Identification of geranic acid, a tyrosinase inhibitor in lemongrass (*Cymbopogon citratus*). J. Agric. Food Chem..

[166] Sabudak T., Khan H.T.M., Choudhary M.I., Oksuz S. (2006). Potent Tyrosinase Inhibitors from Trifolium Balansae. Nat. Prod. Res..

[167] Khan M.T.H., Khan S.B., Ather A. (2006). Tyrosinase inhibitory cycloartane type triterpenoids from the methanol extract of the whole plant of Amberboa ramosa Jafri and their structure-activity relationship. Bioorgan. Med. Chem..

[168] Ullah F., Hussain H., Hussain J., Bukhari I.A., Khan M.T.H., Choudhary M.I., Gilani A.H., Ahmad V.U. (2007). Tyrosinase inhibitory pentacyclic triterpenes and analgesic and spasmolytic activities of methanol extracts of Rhododendron collettianum. Phytother. Res..

[169] Devkota K.P., Khan M.T.H., Ranjit R., Meli Lannang A., Samreen, Iqbal Choudhary M. (2007). Tyrosinase inhibitory and antileishmanial constituents from the rhizomes of *Paris polyphylla*. Nat. Prod. Res..

[170] Azhar-Ul-Haq, Malik A., Khan M.T.H., Anwar-Ul-Haq A., Khan S.B., Ahmad A., Choudhary M.I. (2006). Tyrosinase inhibitory lignans from the methanol extract of the roots of Vitex negundo Linn. and their structure-activity relationship. Phytomedicine.

[171] Lee S., Kim M., Lee S., Ahn Y., Lee H., Lee S.E., Kim M.K. (2000). Inhibitory effects of Cinnamomum cassia bark-derived materials on mushroom tyrosinase. Food Sci. Biotechnol..

[172] Kubo I., Yokokawa Y. (1992). Two tyrosinase inhibiting flavonol glycosides from Buddleia coriacea. Phytochemistry.

[173] Kubo I., Kinst-Hori I. (1999). 2-Hydroxy-4-methoxybenzaldehyde: A potent tyrosinase inhibitor from African medicinal plants. Planta Med..

[174] Ya W., Chun-Meng Z., Tao G., Yi-Lin Z., Ping Z. (2015). Preliminary screening of 44 plant extracts for anti-tyrosinase and antioxidant activities. Pak. J. Pharm. Sci..

[175] Kubo I., Kinst-Hori I. (1999). Flavonols from saffron flower: Tyrosinase inhibitory activity and inhibition mechanism. J. Agric. Food Chem..

[176] Brewer M.S. (2011). Natural Antioxidants: Sources, Compounds, Mechanisms of Action, and Potential Applications. Compr. Rev. Food Sci. Food Saf..

[177] Chai W.M., Wei Q.M., Deng W.L., Zheng Y.L., Chen X.Y., Huang Q., Ou-Yang C., Peng Y.Y. (2019). Anti-melanogenesis properties of condensed tannins from: Vigna angularis seeds with potent antioxidant and DNA damage protection activities. Food Funct..

[178] Biswas R., Chanda J., Kar A., Mukherjee P.K. (2017). Tyrosinase inhibitory mechanism of betulinic acid from Dillenia indica. Food Chem..

[179] Yang Y., Sun X., Ni H., Du X., Chen F., Jiang Z., Li Q. (2019). Identification and Characterization of the Tyrosinase Inhibitory Activity of Caffeine from Camellia Pollen. J. Agric. Food Chem..

[180] Kim J.H., Leem H.H., Lee G.Y. (2020). The guanidine pseudoalkaloids 10-methoxy- leonurine and leonurine act as competitive inhibitors of tyrosinase. Biomolecules.

[181] Yan X., Li J., Liu Z., Zheng M., Ge H., Xu J. (2013). Enhancing Molecular Shape Comparison by Weighted Gaussian Functions. J. Chem. Inf. Modeling.

[182] Liu X., Jiang H., Li H. (2011). SHAFTS: A Hybrid Approach for 3D Molecular Similarity Calculation. 1. Method and Assessment of Virtual Screening. J. Chem. Inf. Model..

[183] Halgren T.A. (1995). Potential energy functions. Curr. Opin. Struct. Biol..

[184] Case D., Cerutti D.S., Cheatham T., Darden T., Duke R., Giese T.J., Gohlke H., Götz A., Greene D., Homeyer N. (2017). Amber 2017.

[185] Hawkins P.C.D., Skillman A.G., Warren G.L., Ellingson B.A., Stahl M.T. (2010). Conformer Generation with OMEGA: Algorithm and Validation Using High Quality Structures from the Protein Databank and Cambridge Structural Database. J. Chem. Inf. Model..

[186] Liu X., Bai F., Ouyang S., Wang X., Li H., Jiang H. (2009). Cyndi: A multi-objective evolution algorithm based method for bioactive molecular conformational generation. BMC Bioinform..

[187] Cregge R.J., Durham S.L., Farr R.A., Gallion S.L., Hare C.M., Hoffman R.V., Janusz M.J., Kim H.O., Koehl J.R., Mehdi S. (1998). Inhibition of human neutrophil elastase. 4. Design, synthesis, X-ray crystallographic analysis, and structure-activity relationships for a series of P2-modified, orally active peptidyl pentafluoroethyl ketones. J. Med. Chem..

[188] Sendovski M., Kanteev M., Ben-Yosef V.S., Adir N., Fishman A. (2011). First structures of an active bacterial tyrosinase reveal copper plasticity. J. Mol. Biol..

[189] Forli S., Huey R., Pique M.E., Sanner M., Goodsell D.S., Olson A.J. (2016). Computational protein–ligand docking and virtual drug screening with the AutoDock suite. Nat. Protoc..

[190] Flanagan D. (2006). JavaScript: The Definitive Guide.

[191] De Volder K., Query J. (2006). A generic code browser with a declarative configuration language. International Symposium on Practical Aspects of Declarative Languages.

[192] Welling L., Thomson L. (2003). PHP and MySQL Web Development.

[193] Harder E., Damm W., Maple J., Wu C., Reboul M., Xiang J.Y., Wang L., Lupyan D., Dahlgren M.K., Knight J.L. (2016). OPLS3: A Force Field Providing Broad Coverage of Drug-like Small Molecules and Proteins. J. Chem. Theory Comput..

[194] Watts K.S., Dalal P., Murphy R.B., Sherman W., Friesner R.A., Shelley J.C. (2010). ConfGen: A conformational search method for efficient generation of bioactive conformers. J. Chem. Inf. Model..

[195] Madhavi Sastry G., Adzhigirey M., Day T., Annabhimoju R., Sherman W. (2013). Protein and ligand preparation: Parameters, protocols, and influence on virtual screening enrichments. J. Comput. Aided. Mol. Des..

[196] Friesner R.A., Murphy R.B., Repasky M.P., Frye L.L., Greenwood J.R., Halgren T.A., Sanschagrin P.C., Mainz D.T. (2006). Extra precision glide: Docking and scoring incorporating a model of hydrophobic enclosure for protein-ligand complexes. J. Med. Chem..

