# Supplementary material for: ANTIAGE-DB: A Database and Server for the Prediction of Anti-Aging Compounds Targeting Elastase, Hyaluronidase, and Tyrosinase
Source: Antioxidants (Basel). 2022 Nov 17;11(11):2268. doi: 10.3390/antiox11112268 (PMC9686885; doi:10.3390/antiox11112268)
Supplement: Supplementary file 1 [file antioxidants-11-02268-s001.zip › antioxidants-1970655-supplementary.pdf]

antioxidants

MDPI

Supplementary Material

ANTIAGE-DB: A Database and Server for the Prediction of Anti-Aging Compounds Targeting Elastase, Hyaluronidase, and Tyrosinase

Christina D. Papaemmanouil [1], Jorge Peña-García [2], Antonio Jesús Banegas-Luna [2], Androniki D. Kostagianni [1], Ioannis P. Gerothanassis [1], Horacio Pérez-Sánchez [2,*] and Andreas G. Tzakos [1,3,*]

[1] Department of Chemistry, Section of Organic Chemistry and Biochemistry, University of Ioannina, 45110 Ioannina, Greece
[2] Structural Bioinformatics and High Performance Computing Research Group (BIO-HPC), Computer Engineering Department, Universidad Católica de Murcia (UCAM), 30107 Guadalupe, Spain
[3] Institute of Materials Science and Computing, University Research Center of Ioannina (URCI), 45110 Ioannina, Greece
* Correspondence: hperez@ucam.edu (H.P.-S.); atzakos@uoi.gr (A.G.T.)

Table of Contents

1. Catalytic Mechanism of Human Neutrophil Elastase (HNE) 4

Figure S1: **(a)** Structure of Human Neutrophil Elastase (PDB ID: 1B0F) and its catalytic triad: Ser195-His57-Asp102 **(b)** Structure of Human Neutrophil Elastase (PDB ID: 1B0F) and its catalytic Ser195 which forms hydrogen bonds with Gly193, creating the "oxyanion hole"........4

Figure S2: **(a)** Illustration of the Human Neutrophil Elastase's catalytic triad (PDB ID: 1B0F) **(b)** Illustration of the main chain interaction between a peptide (substrate or inhibitor) with Human Neutrophil Elastase's subsite pockets (S1-S5)..5

Table S1: Plants, extracts and isolated compounds that have been studied for their inhibitory properties towards HNE...5

Table S2: Studied natural secondary metabolites for their inhibitory activity towards HNE. 12

2. Catalytic activity of Hyaluronidase (Hyal) 15

Figure S3: **(a)** Structure of Hyaluronidase (PDB ID: 2PE4), indicating the catalytic sited amino acids, **(b)** Illustration of Hyaluronidase's catalytic sited amino acids (PDB ID: 2PE4)16

Table S3: Plants, extracts and isolated compounds that have been studied for their inhibitory properties towards Hyaluronidase. 16

Table S4: Studied natural secondary metabolites for their inhibitory potency towards Hyaluronidase. 25

3. Activity of Tyrosinases 25

3.1 Biological activity of Tyrosinases[111] 26

Figure S4: (a) Structure of Tyrosinase (PDB ID: 3NM8), indicating the catalytic sited amino acids. **(b)** Illustration of Tyrosinase's catalytic sited amino acids (PDB ID: 3NM8)...26

3.2 The importance of the number of –OH towards Tyrosinase inhibition.26

Table S5: Plants, extracts and isolated compounds that have been studied for their inhibitory properties towards Tyrosinase..28

Table S6: Studied natural secondary metabolites for their inhibitory properties towards Tyrosinase. 36

4. Software development: the ANTI-AGE Database...37

Figure S5: Representation of the first step of the determination of calculations in ANTIAGE-DB, by inserting the respective SMILES of the studied compound. 38

Figure S6: Detailed representation of the whole procedure for the submission of calculations in the ANTIAGE-DB. The respective arrows are explained in **Section C.2.3**.............39

Table S7: Illustration of the results given with the two softwares (NC-DB and Maestro) a) Elastase with caffeic acid, b) Hyaluronidase with quercetin and c) Tyrosinase with betulinic acid. 39

Table S8: Illustration of the results given with the two softwares (NC-DB and Maestro) for the compound Auricoulasin[42], potent Elastase inhibitor......................................40

REFERENCES 41

1. Catalytic Mechanism of Human Neutrophil Elastase (HNE)

The catalytic mechanism of Human Neutrophil Elastase (HNE) is similar to that of the homologous serino protease chymotrypsin: The first step is a nucleophilic attack of the Oγ atom of Ser195, to the carbonyl group of its substrate[1,2] forming a covalent bond, leading to the formation of a tetrahedral intermediate. This tetrahedral intermediate forms hydrogen bonds with the backbone amides of Gly193 and Ser195 residues, and, thus, its charge becomes stable.[3,4] This hydrogen bonds between the aminoacid residued Gly193 and Ser195 create a structure, called "oxyanion hole".[5] The side chains of His57 and Asp102 form a weak hydrogen bond, enhancing, the nucleophilicity of Ser195. The proton of His57 repels the leaving group of the tetrahedral intermediate, forming the acyl-enzyme intermediate. The enzyme's activation is achieved by two separate amino-terminal processing steps in an optimum pH of 8.0-8.5. Elastase has a positive charge and a basic isoelectric point.[6] Its high basic character is due to the presence of 19 Arg residues, and stabilized by 9 acidic Asp residues, of which Asp-102, Asp-194 and Asp-226 are located in the inner site of its backbone.[7] All Arg residues (except of Arg-80) are located on the enzyme surface, around the active site as clusters of two, three or four arginines. This is the reason for the enzyme's efficiency to bind with linear sulfated polysaccharides.[7,8] In addition, more than 40% of the enzyme's residues are hydrophobic, enabling an easier bond with molecules of high lipophilicity.[7,9]

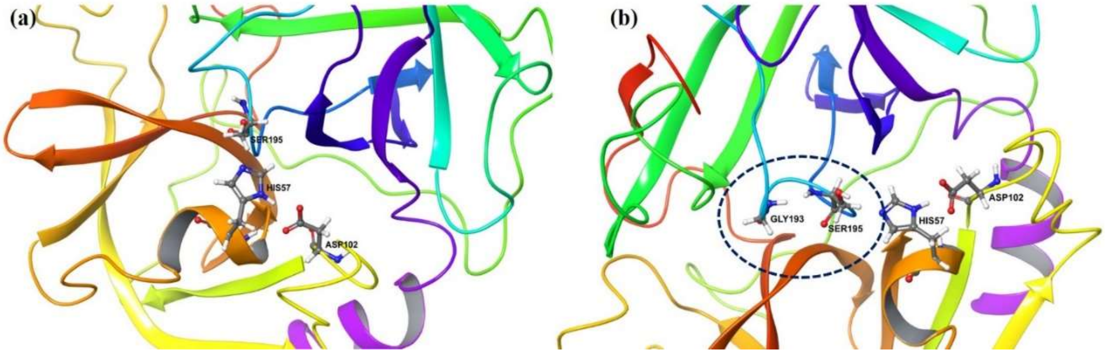

Figure S1. (a) Structure of Human Neutrophil Elastase (PDB ID: 1B0F) and its catalytic triad: Ser195-His57-Asp102 **(b)** Structure of Human Neutrophil Elastase (PDB ID: 1B0F) and its catalytic Ser195 which forms hydrogen bonds with Gly193, creating the "oxyanion hole".

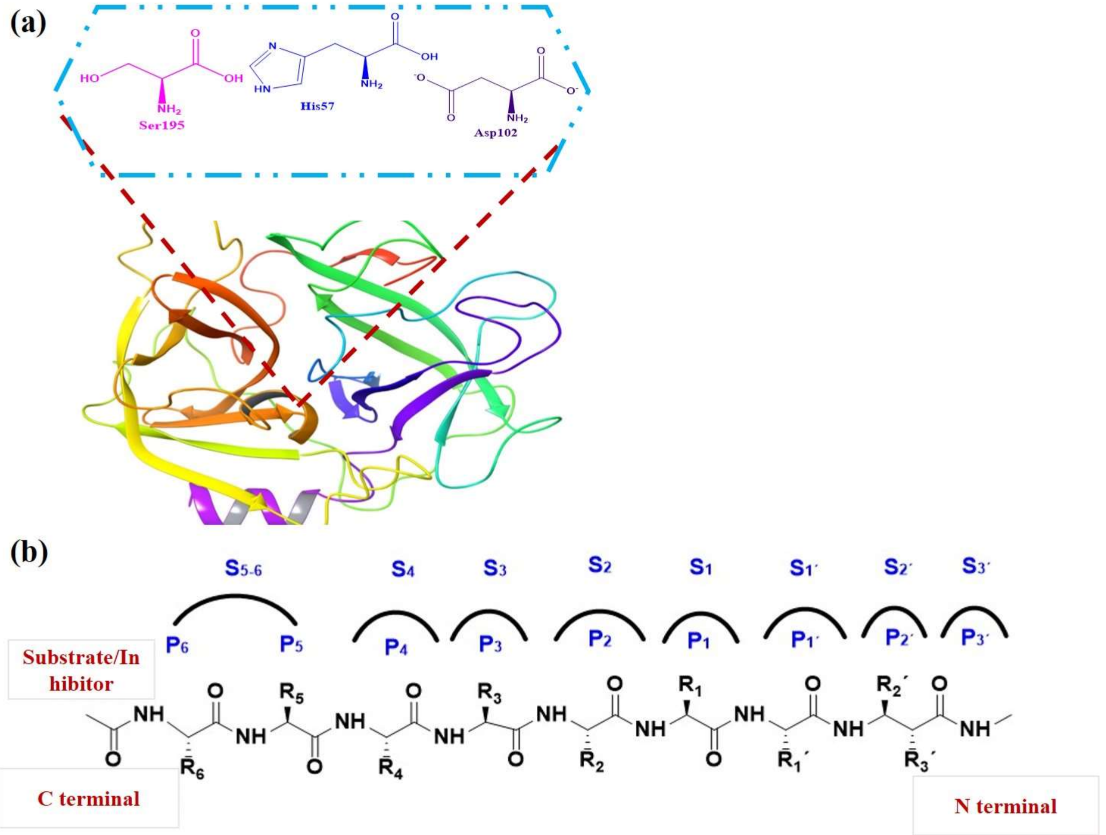

Figure S2. (a) Illustration of the Human Neutrophil Elastase's catalytic triad (PDB ID: 1B0F) **(b)** Illustration of the main chain interaction between a peptide (substrate or inhibitor) with Human Neutrophil Elastase's subsite pockets (S1-S5).

Table S1. Plants, extracts and isolated compounds that have been studied for their inhibitory properties towards HNE.

Plant	*Medicinal use*	*Inhibition towards HNE*	*Ref.*
Camelia Sitensis (Green tea)	Antoxidant, antifungal, antimutagenic, anticarcinogenic, antidiabetic agent, treatment against cardiovascular diseases, and many types of cancer (colon, lung, mouth, esophagus, stomach, kidney, small intestine, pancreas, mammary glands, excellent food intake agent	ECGC (IC_{50}=250 μM)	[10]
Boswellia spp.	Anti-inflammatory properties	Potent inhibition	[10]
Tagetes erecta L. (Marigold) (Compositae)	Skin disorders(sores, wounds, burns, ulcers, eczema etc), kidney problems, muscular pain boils, carbuncles, earache	Methanolic extract:IC_{50}=4.13±0.93 mg/ml<br>Butanoic extract:IC_{50}=4.01±1.37 mg/ml<br>Syringic acid (IC_{50}=34.29±<br>Amyrin (IC_{50}=33.98±1.82 mg.mL	[11]
Ilex paraquariensis St. Hil (Used in Yerba Mate tea) (Aquifoliaceae)	Antioxidant, cellular protective, anti-obesity, thermogenic, circulatory system, hypocholesterolemic and bile	Ethanolic extract (IC_{50}=0.5 μg/ml)<br>Methanolic extract (IC_{50}= 1.38 μg/ml)<br>**Dicaffeoylquinic acid derivatives:**<br>3,5 dicaffeoylquinic acid methyl ester (IC_{50}= 1.4 μM)	[12]

	stimulant regulator, use as tonic and stimulant beverage agent	3,4-dicaffeoylquinic acid methyl ester (IC_{50}=4.2 μM)<br>3,5-dicaffeoylquinic acid (IC_{50}= 2.4 μM)<br>4,5-dicaffeoylquinic acid methyl ester (IC_{50}=1.7 μM)<br>3,4-dicaffeoylquinic acid (IC_{50}=7.3 μM)<br>1,5-dicaffeoylquinic acid (IC_{50}=151 μM)<br>**Monocaffeoylquinic acid derivatives:**<br>Neochlorogenic acid methyl ester, cryptochlorogenic acid methyl ester, chlorogenic acid methyl ester: weak HNE inhibition<br>Quercetin (IC_{50}=1.5 μM)<br>Rutin (IC_{50}= 6.9 μM)<br>Kaempferol 3-*O*-rutinoside: no inhibition	
Cucumis sativus L. (Cucumber) (Cucurbitaceae)	Skin irritations ans disorders (swelling under the eyes, sunburn), healing agent against cooling, healing, soothing, emollient, lenitive and anti itching effects, hyperpigmentation	Juice of C. sativus inhibits 50% of HNE activity at a concentarion of 6.14 μg/ml	[13]
Cimicifuga Racemosa (Black Cohosh) (Ranunculaceae)	Analgesic, sedative and anti-inflammatory agent	Caffeic acid: (IC_{50}=93 μM)<br>Fukinolic acid (IC_{50}=0.23 μM)<br>cimicifugic acid A (IC_{50}= 2.2 μM)<br>cimicifugic acid B (IC_{50}=11.4 μM)<br>cimicifugic acid E (20% HNE inhibition at 50 μM)<br>cimicifugic acid F (IC_{50}= 18 μM)<br>isoferulic acid (IC_{50}> 50 μM)<br>Ferulic acid (IC_{50}>> 500 μM)<br>Elastinal (IC_{50}= 1150 μM)	[8]
Olea europaea L. (Oleaceae)	Diuretic, hypotensive, emollient agent, used for urinary and bladder infections and skin disorders	(E) -2- octenal (potent HNE inhibition)<br>(E) -2-nonenal (potent HNE inhibition)	[14]
Diospyros kaki folium (Persimmon leaf)	Agent against skin disorders, anti-wrinkle agent	Ethanolic fraction II (flavonoid content) (78.1% HNE inhibition at 500 μM)<br>Ethanolic fraction III (polyphenolic content) (28.8% HNE inhibition at 500 μM)	[15]
Ginseng	Antioxidant properties	Extracts inhibit 90% of HNE activity at 0.14 mg/ml	[16]
Actinodaphne lancifolia	Treatments against urinary disorders and diabetes, antioxidant, cytotoxic antidiarrheal, thrombolytic properties	IC_{50}= 103.10 μg/ml)	[17]
Aesculus turbinata	Anti-inflammatory, anti-edematous, capillaro-protective properties, cosmetics and food agent	IC_{50}= 43.10 μg/ml	[17]
Cleyera japonica	Antioxidant, free radical scavenging properties	IC_{50}=205.90 μg/ml	[17]
Cornus controversa	Free radical scavenger, anti-tyrosinase and anti-elastase properties	IC_{50}= 163 μg/ml	[17]

Cornus walteri	Skin anti-inflammatory agent, antioxidant, antidiarrheal, antihyperglycemic, anti-obesity properties	IC_{50}= 26.1 µg/ml	[17]
Cryptomeria japonica	Protection of human keratinocytes	(IC_{50}=108.2 µg/ml)	[17]
Euscaphis japonica	Antioxidant, antitumor agent	IC_{50}=455.90 µg/ml	[17]
Machilus japonica (Kusanoi)	Antimicrobial, anti-α-glucosidase, anti-inflammatory properties	IC_{50}=108.2 µg/ml	[17]
Melia azedarach	Antidiarrheal, ant-malaria, antidiabetic, antidiabetic properties, treatments against rheumatism, asthma, leprosy, eczema, piles, ulcers, toothaches, fevers, snake bites, treatment against skin disorders	(IC_{50}=293.20 µg/ml	[17]
Oenothera erythrosepala	Atioxidant, anti-inflammatory, antidiabetic, anti-bacterial, anti-neuropathic, anti-fungal, anti-diarrheic, cariostatic, antiviral, anti-ulcerogenic, antihelmintic properties, anti-cancer. Anti-tumor agent, treatment against kidney disordes, hepatic disorders, cardiac disorders nematicidal activity, immune response activity, hypocholesterolemic activity, vasorelaxation activity	IC_{50}=87.80 µg/ml	[17]
Rhus javanica	Antioxidant, anti-inflammatory, antibacterial, antiviral, anticancer, antidiarrhoeal, hepatoprotective properties, treatment against dysentery and coughs	IC_{50}=70.5 µg/ml	[17]
Rosa multiflora	Antioxidant, antibacterial properties, skin care cosmetics agent	IC_{50}= 371.90 µg/ml	[17]
Sophora flavescens	Analgesic, antipyretic, anthemintic and stomachic properties	IC_{50}=219.5 µg/ml	[17]
Taxillus yadoriki	Antioxidant, anti-inflammatory, anti-aging, skin-whitening agent, anti-elastase and anti-tyrosinase activity, skin care cosmetics agent	IC_{50}=36.4 µg/ml	[17]

Viburnum odoratissimum	Antioxidant, antiwrinkle properties, skin care cosmetics agent	IC_{50}=80.80 μg/ml	[17]
Areca catechu	Anti-inflammatory, anti-aging properties	IC_{50}=28.10 μg/ml	[17]
Centella asiatica (L.) Urban (Gptu Kola) (Apiaceae)	Various health disorders, anti-aging agent in cosmetics	Methanolic extract (IC_{50}=14.54±0.39 μg/ml)<br>n-butanolic extract (IC_{50}=29.15±0.31 μg/ml)<br>Asiaticoside (IC_{50}=19.45±0.25 μg/ml)	[10,18]
Clitoria ternates L. (Butterfly pea) (Fabaceae)	Central nervous system (CNS) disorders (depression, anxiety, stress etc), skin disorders, antipyretic, anti-inflammatory, analgesic, local anesthetic and antidiabetic agent	Methanolic extract (IC_{50}= 9.61±0.36 μg/ml)	[19]
Grape pomace	Skin anti-aging agent	Polyphenolic extracts (73% HNE inhibition at 35.3 μg/ml, 63% HNE inhibition at 23.5 μg/ml, 49% HNE inhibition at 14.1 μg/ml, 36% HNE inhibiton at 8.8 μg/mland 20% HNE inhibition at 7.1 μg/ml.<br>Fraction abundant in gallic acid (IC_{50}= 47%)<br>Fraction abundant in catechins (IC_{50}= 17%)<br>Fraction abundant in procyanidins (IC_{50}= 19%)<br>Fraction abundant in flavonol-glucosides (IC_{50}=2%)<br>Catechin (IC_{50}=12.0% at 1mmol/l)<br>Epigallocatechin gallate (EGCG) (IC_{50}=7.3% at 1 mmol/l)<br>Procyanidin B2 (6.4% at 1mmol/l)	[20]
Harpagophytum procumbens (Devil's claw)	Skin disorders	6'-O-acetylacteoside (IC_{50}= 70 μM)<br>Isoacteoside (IC_{50}= 286 μM)<br>8-PCHG (IC_{50}= 331 μM)<br>Pagoside (IC_{50}= 260 μM)<br>Harpagoside (IC_{50}~>800 μM)<br>Acteoside (IC_{50}~>800 μM)<br>Cinnamic acid (IC_{50}~>800 μM)	[21]
Vitis vinifera (Grape vine)	Anti-inflammatory and antioxidant agent, free radical scavenger, treatment against cardiovascular diseases	Seeds extract (IC_{50}= 5.4 μM)	[20]
Polypodium species	Treatment against peptic ulcer, kidney disorders, rheumatoid arthritis, psoriasis, skin disorders (dermatitis, vitiligo)	Selligueain (IC_{50}=40 μM in leukocytes)	[3]
Lythrum salicaria L. (Lythraceae)	Anti-inflammatory properties, treatment against haemorrhoidal disease, dysentert, chronic intestinal catarrh, eczema, periodontidis, varicose veins, gingivitis, skin care agent	IC_{50}=37.80±5.9% at 10 μg/ml	[22]
Geum urbanum L. (Rosaceae)	Treatment against periodontitis stomach disorders, anti-	IC_{50}=30.4±4.8% at 10 μg/ml	[22]

	bleeding, anti-inflammatory properties for gums and mucous membranes,		
Rubus idaeus L. (Rosaceae)	Anti-inflammatory, antimicrobial agent, treatments against common cold, fever and flu-like infections	IC_{50}=36.10±0.4% at 10 μg/ml	[22]
Rubus fruticosus L. (Rosaceae)	Antibacterial, antinociceptive, antiproliferative, analgesic properties	IC_{50}=30.70±5.6% at 10 μg/ml	[22]
Potentilla erecta L Raeusch.(Rosaceae)	Antidiarrheal, anti-ulcerogenic, hemostatic, antihemorrhoidal, wound- healing, skin photoprotecting, free radican scavenging agents	IC_{50}=37.40±3.9% at 10 μg/ml	[22]
Filipendula ulmaria L. (Rosaceae)	Dugestive agent, treatment against hetburn, hyperactivity, diarrhoea, gastritis, peptic ulceration, rheumatism, elimination of excess acidity and nauesa	IC_{50}=57.4±5.3% at 10 μg/ml	[22]
Maxim Potentilla anserina L. (Rosaceae)	Anti-inflammatory, wound healing, antitumor, antibacterial, antifungal, antivirues, antidiarrhetic and antidiabetic properties	IC_{50}=7.50±1.0% at 10 μg/ml	[22]
Agrimonia eupatoria L. (Rosaceae)	Antiadhesive, antibacterial, antioxidant, astringent, anti-inflammatory, hepatoprotective properties, treatment against bed wetting, hemorrhagic colitis, liver and urinary disease, cancer, acute diarrhea, diabetes mellitus, inflammation of oral and pharyngeal mucosa, hepatitis B virus	IC_{50}=55.2±4.1% at 10 μg/ml	[22]
Geranium pretense L. (Geraniaceae)	Antidiarrheic, diuretic, tonic, hemostatic, stomachic and antidiabetic agent	IC_{50}=16.10±3.6% at 10 μg/ml	[22]
Geranium robertianum L (Geraniaceae)	Antioxidant, antimicrobial, antidiabetic, antiulcer, neuroprotective, cytotoxic against tumor cells properties, pro-inflammatory agent, food additive	IC_{50}=34.70±4.5% at 10 μg/ml	[22]
Aesculus hippocastanum L. (Hippocastanaceae)	Anti-inflammatory, anti-elastase, venotonic, lymphagogue, anti-oedematous properties	IC_{50}=62.0±6.9% at 10 μg/ml	[22]

Campylotropis hirtella L. (Leguminosae)	Dysmenorrhea, metrorrhagia, metrostaxis, gastric ucers, prostate hyperplasia. Food agent	Ethyl acetate extract (80% HNE inhibition at 100 μg/ml) (2R, 3R)-6-methyl-30-geranyl-2,3-trans-5,7,40-trihydroxy-flavonol (IC^{50}=17.9±1.5 μM, noncompetitive inhibition) (E)-3-(3-(3,7-dimethylocta-2,6-dienyl)-2,4-dihydroxyphenyl)-3,5,7-trihydroxy-chroman-4-one (IC^{50}=8.4±0.8 μM, competitive inhibition) 3'-geranyl-5, 7, 2', 4'-tetrahydroxyisoflavanone (IC^{50}=30.8±1.3 μM, mixed inhibition).	[23-26]
Phillanthus Emblica L. (Amla)	Antioxidant, anti-tyrosinase, anti-wrinkle, antibacterial, anti-inflammatory properties, cosmetic agent	IC^{50}=387.85±8.78 μg/ml	[27]
Manilkara zapota L. (Sapota)	Antixidant, anti-collagenase and anti-elastase properties	Methanolic extract (IC^{50}=35.73±0.61 μg/ml)	[27,28]
Silibum Marianum	Antioxidant, anti-inflammatory, skin photoprotective properties, treatments against skin aking and melanoma development	IC_{50}=38.57±0.04 μg/ml μg/ml	[27,28]
Dodonea viscosa L. (Jack) (Sapindaceae)	Skin, disorders, diabetes, antibacterial, antifungal and anti-inflammattory agent	Aerial parts methanolic extract (75% HNE inhibition at 100 μg/ml) Visconata (IC_{50}=2.4±0.2 μM, noncompetitive inhibition), penduletin (IC_{50}=65.4±0.1 μM, mixed inhibition), 5,6-dihydroxy-3,4',7-trimethoxyflavone (IC50=25.4±0.4 μM, mixed inhibition), viscosine (IC_{50}=150.2±1.2 μM, non responding inhibition), isokaemferide (IC_{50}=93.9±0.6 μM, mixed inhibition), viscosol (IC_{50}=10.9±0.3 μM, mixed inhibition), 5,7-dihydroxy-3'-(2-hydroxy-3-methylbutenyl)-3,6,4'-trimethoxy-flavone (IC_{50}=114.7±0.2 μM, not reported inhibition, 5,7-dihydroxy-3'-(3-hydroxy-methylbutyl)-3,6,40-trimethoxyflavone (IC_{50}=33.4±0.5 μM, mixed inhibition), and 5,7,4'-trihydroxy-3'-(3-hydroxymethylbutyl)-3,6-dimethoxyflavone (IC_{50}=74.7 ±0.3 μM, mixed inhibition)	[29,30]
Grindelia robusta Nutt. (Asteraceae)	Anti-inflammatory, antimicrobial and expectorant agent, catarrhs of the respiratory tract	Quercetin-3-methylether (IC_{50}=19 μM) Quercetin-3, 3'-dimethylether (IC_{50}=129 μM) Quercetagetin-3,6-dimethylether (IC_{50}=115 μM)	[31,32]
Chelidonium majus L. (Papaveraceae)	Gastric ukcer, oral infection, liver disease, anti-cancer, anti-inflammatory and antiviral agent	Aeg/ml)rial part methanolic extract (88% HNE inhibition at 100 **Alkaloids: Isoquinoline** spallidamine (IC_{50} = 11.6 μM) dihydrosanguinarine (IC_{50} =>200 μM), (s)-stylopine (IC_{50}=51.0±0.4 μM, reversible mixed type I), amottianamide (IC_{50}=>200), (+)-chelidonine (IC_{50}>200 μM), spallidamine (IC_{50}= 11.6±1.1 μM, reversible	[33]

		mixed type I) N-trans-feruloyltyramine (IC_{50}=20.7±0.9 μM, reversible mixed type I)	
Epimedium koreanum Nakai (Berberidaceae)	Interfertility, cardiovascular disease, amnesia, lumbago, neurasthenia, arthritis, tonic, immune-modulatory diseases. anti-inflammatory, anti-osteoporosis, anti-oxidant, antidepressant and neuroprotective agent	Ethyl acetate extract (IC_{50}= 35 μg/ml)<br>**Prenylated flavonoids:**<br>epimedokoreanin B (IC_{50}=6.06 μM, reversible mixed type I)<br>5, 7, 4′-trihydroxy-8, 3′-prenylflavone (IC_{50} = of 6.28 μM, reversible mixed type I)	[34–36]
Thuja orientalis L. (Cupressaceae)	Rheumatism, diarrhea, chronic trachetis	Methanolic extract (IC_{50}=5.68 mg/ml)<br>**Flavonoids:**<br>Cupressuflavone (IC_{50} = 8.09±0.92 μM), amentoflavone (IC_{50}=1.27±0.16 μM), robustflavone (IC_{50}= 1.33±0.21 μM respectively)	[37]
Herniaria glabra L. (Caryophyllaceae)	Diuretic disorders, cystitis, irritable bladder, urinary tract infections, urolithiasis	Plant Extract (7.35±1.59% HNE inhibition)<br>Saponin fraction (2.39±1.03% HNE inhibition)<br>Herniariasaponin 14 (HS4) (1.84±0.53% HNE inhibition)	[16]
Rhizophora mucrinata Lam. (Mangrove plant) (Rhizophoraceae)	Antidiabetic, antioxidant, anti-inflammatoryantimicrobial and anti-viralagent, anguna, dysentery, haematuria, ulcers, haemorrhage, diarrhea, nausea, fever, hypertension, constipation, menstruation disorders, leprosy, food agent	Methanolic leaf extract (4.58±0.04 mg CAE/g (catechin equivalent) , methanolic root extract (4.50±0.16 mg CAE/g (catechin equivalent), methanolic twig extract (4.68±0.08 mg CAE/g (catechin equivalent), ethyl acetate fruit extract (4.25±0.25 mg CAE/g (catechin equivalent)	[38]
Campylotropis hirtella (Leguminosae)	Amenorrhea, mestrorhagia, metrostaxis, gastric ulcers, benign prostate hyperplasia, food agent	Ethyl acetate extract (80% HNE inhibition at 100 μg/ml)<br>**Isolated flavonoids:**<br>(2R, 3R)-6-methyl-3'-geranyl-2,3-trans-5,7,4'-trihydroxy-flavonol (IC_{50} =17.9±1.5 μM,<br>(E)-3-(3-(3,7-dimethylocta-2,6-dienyl)-2,4-dihydroxyphenyl)-3,5,7-trihydroxy-chroman-4-one (IC_{50}= 8.4±0.8 μM)<br>3'-geranyl-5,7,2',4'-tetrahydroxyisoflavanone (IC50=30.8±1.3 μM) 3(S)-2',4'-dihydroxy-5,5'dimethoxy-(6'',6''-dimethylpyanoi)-(2'',3'':7,6)-isoflavanon (IC_{50}> 200 μM)<br>3'-geranyl-5,7,2',5'-tetrahydroxyisoflavone (IC_{50} > 200 μM)	[23]
Eriobotrya japonica (Loquat leaves)	Antioxidant, anti-onflammatory agent, treatment	Terpenoid extract (IC_{50}=3.26±0.56 μg/ml)<br>**Isolated Triterpenoids:**<br>Ursolic acid (IC_{50}=8.49±0.42 μg/ml)	[39,40]

	of chronic bronchitis and coughs		
Flemingia Philippinensis (Legumes)	Rheumatism, improvement of bones density, food agent	Methanolic extract (IC_{50}= 87 μg/ml) **Isolated prenylated isoflavones:** genistein IC_{50}=51.4±0.5 μM, noncompetitive inhibition), auriculasin (IC_{50}=3.1±0.2 μM, competitive inhibition), 6,8-diprenylorobol (IC_{50}=1.3±0.3 μM, competitive inhibition), 5,7,3',4'-tetrahydroxy-2',5'-di(3-methylbut-2-enyl) isoflavone (IC_{50}=213.1±1.9 μM, competitive inhibition), flemiphilippinin A (IC_{50}=8.3±0.4 μM, competitive inhibition), 5,7,3'-trihydroxy-2'-(3-methylbut-2-enyl)-4',5'-(3,3-dimethylpyrano)isoflavone (IC_{50}=22.4±0.7 μM, noncompetitive inhibition), 8-γ,γ-dimethylallylwighteone (IC_{50}=6.0±0.3 μM, competitive inhibition), osajin (IC_{50}=26.0±0.6 μM, competitive inhibition), flemingsin (IC_{50}=12.0±0.4 μM, competitive inhibition), **Isolated flavanones:** flemichin D (IC_{50}=5.3±0.5 μM, mixed type I inhibition), lupinifolin (IC_{50}=13.3±0.1 μM, mixed type I inhibition), khonklonginol H (IC_{50}=110.2±0.8 μM, mixed type I inhibition), **Isolated chalcones:** fleminchalcone C (IC_{50}=62.1±0.5 μM, mixed type I inhibition), fleminchalcone A (IC_{50}=76.6±0.9 μM, mixed type I inhibition), fleminchalcone B (IC_{50}=53.2±0.2 μM, mixed type I inhibition) and a flavanol: 6,8-diprenyl-kaempferol (IC_{50}=29.3±0.3 μM, mixed type I inhibition).	[41–43]

Table S2. Studied natural secondary metabolites for their inhibitory activity towards HNE.

Inhibitor	***Chemical Family***	***Plant Source***	***IC_{50}***	***Ref.***
Luteolin	Flavonoids		12 μM	[3]
Chrysin	Flavonoids		6.7 μM	[3]
Naringenin	Flavonoids		Weak inhibition	[3,44]
Eriocitrin	Flavonoids		Weak inhibition	[3]
Gallic Acid Derivatives	Phenolic acids		High inhibition	[45]
Bornylcinnamic acid ester derivatives	Cinammic acid derivatives		1.6-6.9 μM	[3,46]
Cinammic esters	Cinammic acid dervatives		Potent Inhibitor	[3,21,46]
Caffeic acid	Cinammic acid derivatives		93 μM	[8,21,44]
Dicaffeoylquinic acid derivatives	Caffeic acid derivatives	*Asteraceae Phangnalom rupestre*	4.8-10 μM	[12,47–49]

3,5-di-O-caffeoylquinic acid			50% at concentration of 0.2 µM	[50]
Bornyl caffeate	Bicyclic caffeic acid derivative		1.6 µM	[46]
N-octylcaffeic acid			1 µM	[3]
Resveratrol (3,5,4′-trihydroxy-trans-stilbene)	Stilbenes		31 µM and 12 µM	[20,51]
(-)-epigallocatechin-3-gallate	**Catechins**	**Green tea**	0.4µM and 25.3 µM	[52]
{3-[1-(tert-butyldimethylsiloxy)-ethyl]-4-oxo-1-[3, 4, 5-tris (benzyloxy) benzoyl]-azetidin-2-ylidene}-acetic acid ethyl ester	**Monocyclic β-lactam derivatives**		Weak inhibition	[3]
Genistein	**Isoflavone**		HNE release (99 µM when stimulated by Fmlp and 0.5 µM when stimulated by PAF	[51]
Diosmetin	**O-methylated flavone**		83 µM	[3]
Quercetin	Flavonoid		2.4 µM	[3]
Quercetin glycosides	Flavonoid glycosides		0.3-11.1 µM	[3]
Phloretin	Chalcone		>36.5 µM	[3,53]
Viscolin	Chalcone		9.48 µM	[3,54]
Agrimoniin	Elagittanins		0.9 µM	[51]
Pedunculagin	Elagittanins		2.8 µM	[51]
Ellagic acid	Phenolic dilactone	Tea, Red grapes, strawberries, blackberries	Potent inhibition (1.44 µg/ml)<br>88.6% inhibition at a concentration of 4.57 µg/ml	[45,54]
p-cymene	Monoterpene	Nigella Sativa seeds	25 µM	[55]
Thymoquinone	Monoterpene	Nigella Sativa Seeds	30 µM	[55]
Carvone	Monoterpene	Nigella Sativa Seeds	14 µM	[55]
Thymol	Monoterpene	Nigella Sativa Seeds	104 µM	[55]
			18.88±5.21% at 10 µg/ml and 33.25±3.73% at 20 µg/ml	[55]
Carvacrol	Monoterpene	Nigella Sativa Seeds	12 µM	[55]
Ursolic acid	Pentacyclic triterpenes		88.47±2.96% at 1000 µM<br>4.4 µM	[9,18,39,56]
Oleanolic acid	Pentacyclic triterpenes		88.14±3.72% at 1000 µM<br>6.4 µM	[11,18,19,56]
Glycyrrhetinic acid	Pentacyclic triterpenes		75.20±2.89% at 1000 µM	[9]
Glycyrrhizin	Pentacyclic triterpenes		78.66±1.99% at 1000 µM	[9]
Betulinic acid	Pentacyclic triterpenes		82.41±1.37% at 1000 µM	[9]
Lupeol	Pentacyclic triterpenes		93.56±1.19% at 1000 µM<br>1.9 µM	[9]
Canopkyllol	Pentacyclic triterpenes		2.5 µM	[9]

**Germacranolides**	Sesquiterpene lactones		7->200 µM	[57]
4β,15-Epoxy-miller-9Z-enolide	Sesquiterpene lactones		7->200 µM	[57]
15-(3′-Hydroxy)-methacryloyloxy-micrantholide	Sesquiterpene lactones		7->200 µM	[57]
15-(2′,3′-Epoxy)-isobutyryloxy-micrantholide	Sesquiterpene lactones		7->200 µM	[57]
15-(2′-Hydroxy)-isobutyryloxy-micrantholide	Sesquiterpene lactones		7->200 µM	[57]
eupatoripikrin	Sesquiterpene lactones		7->200 µM	[57]
molepantin	Sesquiterpene lactones		7->200 µM	[57]
4β, 15-Epoxy-miller-9E-enolide	Sesquiterpene lactones			[57]
parthenolide	Sesquiterpene lactones		25% at 20µM	[51,57]
scandenolide	Sesquiterpene lactones			[57]
3-acetoxy-costunolide	Sesquiterpene lactones		7->200 µM	[57]
7-hydroxy-costunolide	Sesquiterpene lactones		7->200 µM	[57]
Guaianolides	Sesquiterpene lactones		7->200 µM	[57]
2-oxo-Guai-1(5)-en-12,8α-olide	Sesquiterpene lactones		7->200 µM	[57]
thieleanin	Sesquiterpene lactones		7->200 µM	[57]
eminensin	Sesquiterpene lactones		7->200 µM	[57]
Podachaenin	Sesquiterpene lactones		7 µM	[57]
Pseudoguaianolides	Sesquiterpene lactones		7->200 µM	[57]
11α,13-Dihydrohelenalin-methacrylate	Sesquiterpene lactones		7->200 µM	[57]
11α,13-Dihydrohelenalin-acetate	Sesquiterpene lactones		7->200 µM	[57]
Eudesmanolide	Sesquiterpene lactones		7->200 µM	[57]
1β-Acetoxy-4α-hydroxy-15-isobutyryloxy-eudesma-11(13)-en-12,8β-olide	Sesquiterpene lactones		7->200 µM	[57]
11α, 13- Dihydrohelenalin acetate	Sesquiterpene lactones		-2-2%	[57]
Eudesmanolides	Sesquiterpene lactones		-2-2%	[57]
alantolactone/isoalantolactone 3:1	Sesquiterpene lactones		-2-2%	[57]
Bolinaquinone	Sesquiterpene	Dysidea spec.	5.3 µM	[57]
Aminoquinone dysidine	Sesquiterpenes	Dysidea spec.	1.3 µM	[57]
Dysidone A:Dysidone B (1:1)	Sesquiterpenes	Dysidea spec.	10 µM	[57]
Dehydrocostic acid	Sesquiterpenic acid	Inula Viscosa	Potent inhibition	[57]
Erucic acid (22:1, cis-13)	Fatty acids		0.45 µM	[44]
Oleic acid (18:1, cis-9)	Fatty acids		5 µM	[44]
Stearic acid (18:00)	Fatty acids		10 µM	[44]
Palmitic acid (16:00)	Fatty acids		15 µM	[44]
Eicosapentaenoic acid (20:5)	Fatty acids		No inhibition	[44]
Docosahexaenoic acid (22:6)	Fatty acids		No inhibition	[44]
Myristic acid (14:00)	Fatty acids		35 µM	[44]
Pentadecanoic acid (15:00)	Fatty acids		25 µM	[44]
Heptadecanoic acid (17:00)	Fatty acids		>50 µM	[44]
Nonadecanoic acid (19:00)	Fatty acids		>50 µM	[44]
Arachidic acid (16:01, cis-9)	Fatty acids		20 µM	[44]
Behenic acid (22:00)	Fatty acids		30 µM	[44]
Palmitoleic acid (16:1, cis-9)	Fatty acids		20 µM	[44]
Linoleic acid (18:2, cis-9,12)	Fatty acids		10 µM	[44]
Linolenic acid (18:3, cis-9,12,15)	Fatty acids		15 µM	[44]
γ-linolenic acid (18:03. cis-6,9,12)	Fatty acids		15 µM	[44]

Myrtucommulone	Acylphloroglucinols	*Myrtus Communis* leaves extracts	(0.4-3.8 μM)	[3]
Semimyrtucommulone	Acylphloroglucinols	*Myrtus Communis* leaves extracts	(0.4-3.8 μM)	[3]
Hyperforin	Acylphloroglucinols	*Hypericum Perforatum* extracts	(0.4-3.8 μM)	[3]

2. Catalytic activity of Hyaluronidase (Hyal)

The degradation of HA into its oligomeric fragments, takes main place by Hyal-1 and Hyal-2 at acidic pH conditions. When Hyal-1 interacts with HA, the *N*-acetylated carbonylic oxygen of HA, which is of high nucleophilicity, is rotated next to the C1 carbon, in order to form β-1, 4 glycosidic bond, leading to the formation of a new glycosidic product. Then, the protonated amino acidic residue Glu131 gives its H atom to the glycosidic oxygen, which consists the leaving group of HA. The next step includes hydrolysis of anomeric C1 in the active site, which reprotonates Glu131. The final result is the separation of HA from the active site of Hyal-1 leading to a new glycosidic product.[58] Hyals' activity is regulated by a lot of factors, the most important of which are the pH value and the concentration of the substrate,[60] as well as an activating ion which exists in the reaction mixture, for example Ca^{2+} (or Na^{2+}).[60–62] Hyals can be found in nature, in many living organisms, like mammals, as well as insects, leeches and bacteria.[63–65] The latter are also able of producing Hyals. Hyals produced by eukaryotes act both as hydrolases and transglycosidases, whereas Hyals broduced by bacteria form β-elimination reactions.[66]

All the Hyal structures have a domain similar to the $(\beta\alpha)_8$ /TIM barrel structure of bee venom hyaluronidase (BVHyal), although they differ in the number of sheets. One domain of this barrel forms a large, elongated cleft, where the amino acids are arranged into specific places, enabling the interaction with HA. Hyal-1 has the smallest amino acid sequence (435 amino acids). In contrast, PH-20 has the largest amino acid sequence (510 amino acid residues).[58] Hyal-1's amino terminal domain contains 28 amino acids, whereas PH-20's amino terminal domain contains 41 amino acids. In addition, Hyal-1 and Hyal-2 have a single helix domain, whereas Hyal-3, Hyal-4 and PH-20 have two helix domains. All the helixes are placed in the external side of the TIM barrel. The catalytic Carboxylic terminals of all the human hyaluronidases have a second domain next to them, smaller than the catalytic region, but bigger than the *N*-amino terminal sequence and differs in all the Hyals (68 amino acids in Hyal-1 whereas 122 amino acids in PH-20). This domain also differs among all the human Hyals: In Hyal-1 it is a triple antiparallel β-sheet surrounded by one helix on this side, whereas the carboxylic terminal has different structure. In Hyal-2 it has two helices, whereas in Hyal-3 it contains two helices covered with the catalytic region. In Hyal-4 it has three helices, covered by an antiparallel double β-sheet. In contrast, in PH-20 it contains eight helices, five of which are very long.[67] As glycosylphosphatidylinositol-(GPI)-anchored proteins, Hyal-2, Hyal-4 and PH-20 have a GPI-like sequence in their Carboxylic terminal. The different sequence of the carboxylic amino acids regulates the different mechanistic properties of each enzyme.

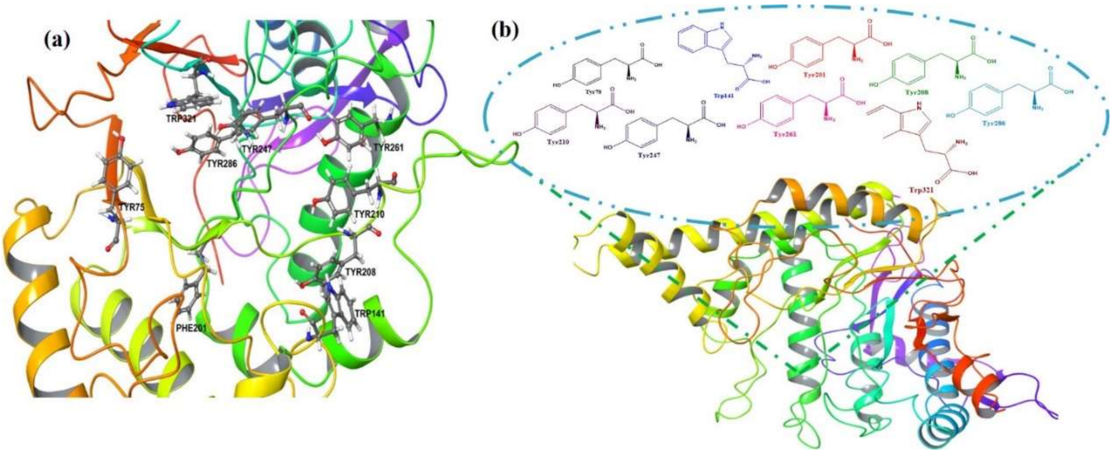

Figure S3. (a) Structure of Hyaluronidase (PDB ID: 2PE4), indicating the catalytic sited amino acids, **(b)** Illustration of Hyaluronidase's catalytic sited amino acids (PDB ID: 2PE4).

Table S3. Plants, extracts and isolated compounds that have been studied for their inhibitory properties towards Hyaluronidase.

Plant	*Medicinal use*	*Inhibition towards Hyal*	*Ref.*
Chamaerhodos erecta	Treatments againsts hepatic disorders, rheumatism, scurvy, high temperature, meal poisong, scorbutus, arthritis, tachycardia, face and foot swelling and hemorrhage	Aerial part butanolic extract potent Hyal inhibition (2R,3S)-3,4-dihydro-2-(3,4-dihydroxyphenyl)-2H-chromene-3,5,7-triol (IC_{50}= 0.842 mM), 1,2,3,4,6-penta-O-galloyl-b-D-glucopyranoside (IC_{50}=0.595 mM), eugeniin (IC_{50}=0.509 mM), 1,2,6-tri-O-galloyl-b-D-glucopyranoside (IC_{50}=0.792 mM), potentillin (IC_{50}=0.890 mM), agrimoniin (IC_{50}=0.578 mM) rosmarinic acid (IC_{50}=1.363 mM)	[68]
Chamaerhodos Altaica	Antiinflammatory properties, skin care cosmetics agetn	Aerial part aqueous extract potent Hyal inhibition	[68]
Dracocephalum foetidum	Antimicrobial properties, anti-hyalurondase agent	**Isolated compounds:** (R)-a-[[(2E)-3-[4-[[(1Z)-1-carboxy- 2-(3-hydroxy-4-methoxyphenyl)ethenyl]oxy] -3-hydroxyphenyl]-1-oxo-2-propen-1-yl]oxy]-3,4-dihydroxy-benzenepropanoic acid (IC_{50}=0.22± 0.01 mM), rosmarinic acid (IC_{50}=0.75±0.04 mM), acacetin-7-O-(3,6-O-dimalonyl)-b-D-glucopyranoside (IC_{50}= 0.25±0.01 mM), acacetin-7-O-(3-O-malonyl)-b-D-glucuronopyranoside (IC_{50}=0.19±0.02 mM), acacetin-7-O-b-D-glucuronide (IC_{50}=0.55±0.14 mM), apigenin 7-O-(6-malonyl-beta-D-glucoside) (IC_{50}=0.99±0.12 mM), apigenin 7-O-b-glucuronide (IC_{50}= 0.56± 0.07 mM) and luteolin-7-O-b-D-glucuronide (IC_{50}= 0.79±0.04 mM).	[69]
Gaultheria procumbens L. (Eastern teaberry)	Anti-inflammatory, analgesic properties, treatment against acute and chronic prostatitis, rheumatoid arthritis, chronic tracheitis, swelling pain	Ethyl acetate extract (IC_{50}= 21.83±0.82% at 100 μg/ml)	[70–72]
Oenothera biennis L.	Anti-diabetic, anti-inflammatory, antibacterial and	Aerial part methanolic extract (potent Hyal inhibition)	[73]

	antifulgal properties, Treatment against hyperlipidemia, atherosclerosis, atopic dermatitis, endothelial dysfunction, peptic ulcer, ulcerative colitis, Crohn's disease		
Payena Dasyphylla Bark	Anti-inflammatory and antioxidant properties	Methanolic extract (IC_{50}= 91.63% at 100 µg/ml)	[73,74]
		Ethyl acetate extract (Hyal-1 and Hyal-2 inhibition at 100 µg/ml)	
Borago officinalis L.(Borage) (Boraginaceae)	Antioxidant, antispasmodic, antihypertensive, antipyretic, aphrodisiac, demulcent, diuretic properties, treatment against asthma, bronchitis, cramps, diarrhea, palpitations, kidney ailments	Leaves extracts (IC_{50}=71.6±5.4%)	[75]
Spinacia oleracea L. (Spinach) (Chenopodiaceae)	Antioxidant, free radical scavenging, anti-cancer, anti-obesity, hypoglycemic and hypolipidemic properties, high nutraceutical value	Leaves extarcts (IC_{50}= 92.3±1.8%)	[75]
Lactuca sativa (Compositae)	Antioxidant, anticancer agent, neutraceutical agent	Leaves (IC_{50}=110.5±0.3%)	[75]
Arctium lappa L. (Lettuce) (Compositae)	Anti-diabetic, anti-obesity, anti-tumor properties	Roots (no Hyal inhibition)	[75]
Chrysanthemum coronarium L. (Compositae)	Anti-inflammatory, diuretic, nutritive, bllod purification, fluid retention properties, cosmetic agent	Leaves (no inhibition)	[75]
Lepidium sativum L. (Cress) (Gruciferae)	Analgesic, anti-spasmodic, hepatoprotective, anti-diarrhoeal, antioxidant, anti-inflammatory, diuretic and galactagogue properties	Leaves (IC_{50}=89.4±3.0%)	[75]
Eutrema wasabi Maxim. (Japanese horseradish stem) (Gruciferae)	Anti-inflammatory, anti-microbial, anti=platelet, anticancer, antioxidant and antidiabetic agent, high nutraceutical value	Stem (IC_{50}=91.4±1.1%)	[75]
Rapharus sativus L. (Japanese radish) (Gruciferae)	Antioxidant, antimicrobial properties. Treatment against respiratory urinary, gastrointestinal systems disorders, female and male infertilit, anemia, skin disorders	Root (IC_{50}=107.2±2.1%).)	[75]
Brassica oleracea L. (Gabbage) (Gruciferae)	Antioxidants and anticancer properties	Leaves (no Hyal inhibition)	[75]

Brassica campestris L. (Chinese cabbage)	Leucorrhoea, menstrual disorders, gleets, body weakness, internal pain	Leaves (no Hyal inhibion)	[75]
Melissa officinalis L. (Lemno balm) (Labiatae)	Hypoglycemix, hepatoprotective, antimicrobial, antidepressant, hypnotic and sedative agent, Treatment against breast cancer and colon carcinoma Food agent, uses in aromatotherapy	Leaves (IC_{50}= 1.0±0.3%),	[75]
Mentha piperita L. Peppermint) (Labiatae)	Biliary disorders, dyspepsia, enteritis, flatulence, gastritis, intestinal colic, spams of the bile duct, gallbladder and gastrointestinal tract	Leaves (IC_{50}=26.5±14.4%)	[75]
Perilla ocymoides L. (Perilla) (Labiatae)	Treatment against cold, headache, cough, abdominal fullness and distention, poisoning from fish and crabs, flavor agent	Leaves (IC_{50}=80.5±4.4%),	[75]
Rosmarinus officinalis L. (Rosemary) (Labiatae)	Antibacterial, antioxidant, antifungal and antitumor agent, Food agent, cosmetic agent	Leaves (IC_{50}=35.6±13.2%)	[75]
Salvia officinalis L. (Sage) (Labiatae)	Antioxidant, anti-inflammatory, hypoglycemic, antibacterial, antitumor agnet, Treatment against Alzheimer's disease, Flavor agent, cosmetic agent	Leaves (IC_{50}=15.5±10.6%)	[75]
Satureja hortensis L. (summer savoy) (Labiatae)	Antioxidant, antimicrobial, antiparasitic, pesticidal, anti-inflammatory, antinociceptive, hepatoprotective, anticancer agent.	Leaves (IC_{50}=30.8±8.1%),	[75]
Ocimum basilicum L. (Sweet basil) (Labiatae)	Antioxidant, anti-spasmodic, anti-diabetic, anti-bacterial, anti-fungal agent. Control of blodd pressure, treatments against coughs, headaches, infections, stomach aches and constipation.	Leaves (IC_{50}=60.2±7.1%)	[75]
Majorana hortensis Moench (Sweet marjoram) (Labiaatae)	Antioxidant, antiproliferative, antimutagenic, antimicrobial agent. Control of platelet aggregation. Treatments against cough, rheumatism, indigestion, toothache. Treatment against gastric and cardiovascular disorders,	Leaves (IC_{50}=23.5±7.2%)	[75]
Thymus vulgaris L. (Thyme) (Labiatae)	Antioxidant, antimibrobial, anti-inflammatory, antifungal agent.	Leaves (IC_{50}=35.5±12.8%).=	[75]

	Treatment against acne and other skin disorders, anxiety, laryngitis, coughs, liver disfunction, menstrual cramps, premenstrual syndrome, infections of urinary tract		
Vicia faba L. (Leguminosae)	Antioxidant, anti-fungal, anti-diabetic, anticancer agent.	Seeds (IC_{50}=90.0±2.1%)	[75]
Pisum sativum L. (Garden pea) (Leguminosae)	Antioxidant, antidiabetic, antifungal, anti0inflammatory, antilipidemic and anticancer properties. Cosmetic agent	Pods (no inhibition)	[75]
Vigna radiata R. Wilez. (Mung bean) Lequminosae)	Antioxidant, anti-inflammatory, antibacterial, antitumor, hypolipidemic, antidiabetic, detoxication, and hepatoprotective agent, Cosmetic agent, food agent	Sprout (IC_{50}=94.1±2.1%)	[75]
Phaseolus vulgaris L.(Snap bean) (Leguminosae)	Analgesic, anti-obesity, antibacterial, anticancer, antidiabetic, antifertility, anti-inflammatory, antioxidant, hepatoprotective, hypolipidemic, litholytic agent. Inhibitor of trypsin and α-amylase.	Pod (IC_{50}=89.1±2.1%)	[75]
Rheum rhaptonticum L. (Polygonaceae)	Antioxidant, antimicrobial, antifungal, anti-inflammatory agent	Stalk (IC_{50}=93.5±0.7%),	[75]
Fragaria Xananassa Duch.(Strawberry) (Rosaceae)	Antioxidant, cholesterol lowering, anticancer, antioxidant and anti-againg agent. Treatment against oral diseases and disorders of urinary tract. Treatment against leukemia	Root (IC_{50}=87.6±2.7%)	[75]
Daucus carota L. (Carrot) (Umbelliferae)	Antioxidant, anticancer, ati-diabetic-anti-hypertensive,hepatoprotective, wound healing, antibacterial, antifungal, cardioprotective, anti-inflammatory, analgesic, fertility properties.	*Root (*IC_{50}=104±1.7%),	[75]
Apium graveolens L. (Celery) (Umbelliferae)	Anti-diabetic, antifungal, anti-inflammatory, anticoagulant agent, reatment against cardiovascular and gastrointestinal disorders	*Stalk (*IC_{50}=108.8±3.9%)	[75]
Coriandrum sativum L. (Coriander) (Umbelliferae)	Antioxidant, antifungal and antibacterial agent, Treatment against cognition, dementia,	*Leaves (no inhibition)*	[75]

	anxiety, flavoring agent, cosmetic agent		
Anethum graveolens L. (Dill) (Umbelliferae)	Antimicrobial, anti-inflammatory, analgesic, hyperlipidemic, agent, treatments of gastrointestinal disorders, treatments against disorders of the reproductive system	*Leaves* IC_{50}=88.1±2.8%)	[75]
Foeniculum vulgare Mill. (Fennel) (Umbelliferae)	Antifungal, antibacterial, antioxidant agent	*Stalks* (IC_{50}=94.1±1.5%)	[75]
Petroselinum crispum Nym. (known as Parsley)	Antimicrobial, hypotensive, diuretic, laxative and antispasmolitic agent	*Leaves* (IC_{50}=88.1±3.4%)	[75]
Capsicum anmuum L. (Pepper) (Solanaceae),	Antioxidant, antimicrobial, antitumor properties. Treatment of rheumatism, stiff joints, bronchitis, chest colds, arthritis, heart arrhthmias, osteoarthritis	*Fruit* (IC_{50}=79.5±10.5%)	[75]
Solanum melongena L. (Eggplant) (Solanaceae).	Antihaemorrhoidal, astringent and hypotensive properties. Reduction of blood cholesterol levels, antidote to poisonous mushrooms, wound healing agent, treatment of intestinal hemorrhages, piles and toothache.	Fruit (no inhibition)	[75]
Lycopersicon esculentum mill.(Tomato) (Solanaceae)	Antioxidant, antimicrobial, antidiabetic, anti-inflammatory. Treatment of dysentery, back pain, rheumatism, lowering cholesterol, rheumatism, proper functioning of brain	Fruit (no inhibition)	[75]
Colocasia esculenta Schott (Taro) (Araceae)	Antimicrobial, antihepatotoxic, antidiabetic, anti-lipid, anti-inflammatory, antifungal, antimetastatic properties	Tuber extracts (no Hyal inhibiton)	[75]
Cucumis Sativus L. (Cucumber) (Cucurbitaceae)	Antioxidant, antimicrobial, anti-aging properties. Glycemic reduction, improving immunity and boosting metabolism properties	Fruit (no Hyal inhibiton)	[75]
Cucurbita maxima Duch. (Pumpkin) (Cucurbitaceae)	Antioxidant, antimicrobial, anticancer agent. Control of blood glucose levels. Treatmens of urinary tract diseases.	Fruit (no Hyal inhibition)	[75]
Asparagus officinalis L. (Asparagus) (Liliaceae)	Antibiotic, diuretic, antispasmodic, anticancer, laxative, sedative, aperient	Sprout (no Hyal inhibition)	[75]

	properties. Treatment against cardiovascular diseases.		
Allium tuberosum Rottler (Chinese chive)(Liliaceae)	Treatment against asthma, abdominal pain, diarrhea and diabetes. Aphrodisiac agent	Leaf (no Hyal inhibition)	[75]
Allium schoenoprasum L. (Chive) (Liliaceae)	Antioxidant properties	Leaf (no Hyal inhibition)	[75]
Allium sativum L. (Garlic) (Liliaceae)	Wound healing, anticancer, antioxidant, anti-inflammatory antidiabetic properties. Treatment against skin, disorders, urinary diseases, kidney stone, epilepsy, cataract. asthma, arthritis, bronchitis, chronic fever e.c Spice and flavoring agent	Buld (no Hyal inhibition)	[75]
Allium cepa L. (Onion) (Liliaceae)	Antioxidant, anti-inflammatory, antimicrobial, analgesic, anti-diabetic, anti-hypertensive, hypolipidemic and immunoprotective properties.	Buld (no Hyal inhibition)	[75]
Allium fistulosum L. (Welsh onion) (Liliaceae)	Anti-microbial, anti-fungal, anti-termite, wound healing agent. Treatment against common cold, eyesight disorders, headache, heart disorders. Reduces serum lipid concentrations and fat accumulation, food agent	Leaf (no Hyal inhibition)	[75]
Abelmoschus esculentus Moench (Okra) (Malvaceae)	Antioxidant, antitumor properties. Treatment against type 2 diabetes, cardiovascular disease and digestive disorders	Pod (no Hyal inhibition)	[75]
Zingiber officinale Rosc. (Ginger) (Zingiberaceae)	Antioxidant, anticancer, antimicrobial, antidiabetic, hepatoprotective, nephroprotecyive, immunomodulatory, anti-inflammatory, larvicidal, analgesic properties	Root (no Hyal inhibition)	[75]
Zingiber mioga Rose (Mioga ginger) (Zingiberaceae)	Anti-inflammatory, antimicrobial, anticancer, anti-platelet aggregation properties, treatment against rheumatism and coughing	Flower (no Hyal inhibition)	[75]
Areca catechu[77]⁷⁷⁷⁶⁷⁶⁷⁶<sup>67</sup	Skin disorders	Ethanolic extract (>57% Hyal inhibition at 250 μg/ml and >82% at 500 μg/ml. IC_{50}=330 μg/ml)	[76]

>⁶⁵⁶¹			
Glycyrrhiza uralensis	Skin disorders	Plant extract (10-78% Hyal inhibition at a range of 100-1000 µg/ml, IC_{50}=210 µg/ml)	[76]
Althaea officinalis (Marshmellow)	Pharygeal irritation, gastrointestinal disorders	Extracts (IC_{50}=7.7 mg/ml for Hyal-1) scopoletin-7-O-α-L-rhamnopyranosyl-(1″→6′)-β-D-glucopyranoside (IC_{50}=84%) hypolaetin-8-O-β-D-glucopyranosyl-(1‴→4″)-β-D-glucuronopyranoside (IC_{50}=73%) 4′-O-methylhypolaetin-8-O-β-D-(2″-O-sulfo)glucopyranoside (IC_{50}=73%) 4′-O-methylhypolaetin-8-O-β-D-(2″-O-sulfo)glucopyranoside (IC_{50}=83%)	[77]
Allium sativum L. (Garlic)	Metabolic disorders, food spice	quercetin (IC_{50}=23.0 mM), isoquercitrin (quercetin 3-O-β-D-glucopyranoside) (IC_{50}=20.9 mM) reynoutrin (quercetin-3-O-β-D-xylopyranoside) (IC_{50}=22.1 mM) kaempferol (IC_{50}=36.3 mM) astragalin (kaempferol 3-O-β-D-glucopyranoside) (IC_{50}=26.5 mM) isorhamnetin (IC_{50}=55.4%) isorhemnetin 3-O-β-D-glycopyranoside (IC_{50}=50.4 mM)	[78]
Hennae folium	Anti-inflammatory, antidiarrhetic properties, skin protective agent	(IC_{50}=no reported, Inhibition 0%) at 10 mg/ml	[79]
Equiseti herba	Anti-inflammatory, antibacterial properties, Treatment against urinary tract infections	(IC_{50}=1.5 mg/ml, inhibition 100%)	[79]
Betulae folium	Anti-inflammatory properties, treatmenst against arthritis	(IC_{50}=no reported, Inhibition 61%)	[79]
Ononidis radix	Anti-inflammatory and diuretic properties	(IC_{50}=1.7 mg/ml, Inhibition 81%)	[79]
Buchu folium	Anti-inflammatory, treatment against urinary tracy infections and kidney disorders	(IC_{50}=no reported, Inhibition 21%)	[79]
Maydis stigma	Antioxidant, diuretic agent, reduces hyperglycemia, anti-fatigue and anti-depressant properties	(IC_{50}= no reported, Inhibition 4%)	[79]
Malvae sylvestris flos	Anti-inflammatory, diuretic properties, Treatment against circulatory, central nervous system, dermatological, digestive, gynecological and metabolic disorders	(IC_{50}=1.4 mg/ml, inhibition 100%)	[79]
Solidaginis herba	Anti-inflammatory, antibacterial, treatments against the infections of the urinary tract	(IC_{50}=4.9 mg/ml, Inhibition 100%)	[79]
Chebulae fructus	Anti-inflammatory, treatments against diarrhea, bleeding,	(IC_{50}=no reported, Inhibition 0%)	[79]

	chronic bronchitis, chronic laryngitis, ulcers, bacillary dysentery and tonsillitis		
Coptis rhizome	Anti-inflammatory properties, treatments against typhoid, bacillary dysentery, tuberculosis, pertussis, epidemic cerebrospinal meningitis	(IC_{50}=no reported, Inhibition 0%)	[79]
Cranberry	Anticancer, diuretic, antipyretic, antiseptic, antidiabetic properties, treatment against chronic fatigue syndrome, pleurisy and scurv	(IC_{50}=no inhibition, Inhibition: 10%),	[79]
Althaeae radix	Anti-inflammatory, diuretic, astringent, cooling, febrifuge, expectorant, emmenagogue, demulcent agent, Tretment against skin, kidney and uterus disorders	(IC_{50}=no inhibition, Inhibition: 60%),	[79]
Hydrastis rhizoma	Anti-inflammatory agent, Treatment against circulatory, cardiovascular, central nervous system, dermatological, digestive, gynecological, metabolic, respiratory and urinary disorders	(IC_{50}=no inhibition, Inhibition: 7%),	[79]
Mahonia radix	Anti-inflammatory, wound healing agent, treatment against tuberculosis, dysentery, periodontitis, eczema, pharyngolaryngitis	(IC_{50}=no inhibition, Inhibition: 26%).	[79]
Palaquium gutta	Anti-inflammatory agents, treatment against mouth disorders	Methanolic bark extract: (IC50=88.2%),	[74]
Pouteria obovatta	Anti-inflammatory, treatments against skin disorders	Methanolic bark extract: (IC50=90.47%)	[74]
Payena dasyphylla	Anti-inflammatory agent, Treatment against arthritis	Methanolic bark extract: (IC50=91.63%)	[74]
Uncaria villosa	Anti-inflammatory and antioxidant properties	Methanolic bark extract: (IC50=55.20%)	[74]
Palaquium qutta	Anti-inflammatory agent, treatment against mouth disorders	Leaf extract: (IC50=51.35%)	[74]
Pauteria oobova	Anti-inflammatory, treatment against skin disorders	Leaf extract: (IC50=55.63%)	[74]
Onion	Antioxidant, antibacterial and anti-inflammatory agent, nutraceutical agent	Quercetin (IC_{50}=27% at 750 μM)<br>quercetin 3,4 diglucoside ((IC_{50}=38% at 750 μM)	[78]
Lythrum salicaria L. (Lythraceae)	Anti-inflammatory agent, treatment against dysentery,	(IC_{50}=64.9±6.3% at 10 μg/ml)<br>Flower extract (IC_{50}=94.4±0.6% at 20 μg/ml)	[22]

	eczema, haemorhoidal disease, chronic intestinal catarrh, periodontosis, gingivitis and varicose veins	**Isolated elagitannins:**<br>Salicarinin A (IC_{50}=1.06±0.1 µM)<br>Salicarinin (IC_{50}=1.6±0.2 µM)<br>Salicarinin C (IC_{50}=2.5±0.2 µM)<br>Vescalagin (IC_{50}=3.1±0.2 µM)<br>Castalagin (IC_{50}=3.1±0.2 µM)	
Geum urbanum L. (Rosaceae)	Treatment against periodontitis stomach disorders, anti-bleeding, anti-inflammatory properties for gums and mucous membranes	(IC_{50}=25.6±5.1% at 10 µg/ml)	[22]
Rubus idaeus L. (Rosaceae)	Antioxidant, antibacterial, antioxidant, antitumor properties, treatment against uterous disorders	(IC_{50}=21.2±2.0% at 10 µg/ml)	[22]
Rubus fruticosus L. (Rosaceae)	Antibacterial, antinociceptive, antiproliferative, analgesic properties	(IC_{50}=12.5±6.8% at 10 µg/ml)	[22]
Potentilla erecta L Raeusch (Rosaceae)	Antidiarrheal, anti-ulcerogenic, hemostatic, antihemorrhoidal, wound- healing, skin photoprotecting, free radican scavenging agents	(IC_{50}=5.8±4.1% at 10 µg/ml)	[22]
Filipendula ulmaria (L) (Rosaceae)	Anti-inflammatory, antipyretic, analgesic, anti-rheumatic and astringent properties	(no inhibition at 10 µg/ml)	[22]
Maxim Potentilla anserine L. (Rosaceae)	Wound healing, homeostatic agent, Treatment against tooth ache, dysentery, ulcers of the mouth, inflammations of the throat	(no inhibition at 10 µg/ml)	[22]
Agrimonia eupatoria L. (Rosaceae)	Antioxidant, anti-inflammatory, astringent and diuretic properties	(no inhibition at 10 µg/ml)	[22]
Geranium pratense L. (Geraniaceae)	Analgesic, febrifuge, anti-inflammatory agent, Treatment against inflammation of the lungs, influenza, pain and swellings of the limbs	(IC_{50}=16.1±3.6 at 10 µg/ml)	[22]
Geranium robertianum L. (Geraniaceae)	Anti-inflammatory, antibacterial, antidiabetic, anti-cancer, antiallergic, diuretic and haemostatic properties	(IC_{50}=7.2±3.8% at 10 µg/ml)	[22]
Aesculus hippocastanum L. (Hippocastanaceae)	Anti-inflammatory agent, treatment against venous bites, bronchitis, dysentery and hemorrhoids	(no inhibition at 10 µg/ml)	[22]
Eleutherococcus spp. Inflorescences	Antioxidant and anti-inflammatory agent	*E. gracilistylus* (16.4±0.05% Hyal inhibition), *E. giraldii* (60.7±0.01%, Hyal inhibition), *E. senticosus* (57.5±0.05% Hyal inhibition).	[80]

Humulus Lupulus L.<br>(Hop Flowers)	Inhibition of bone resorption. Nitric oxide production. Anticancer agent. Estrogenic activity, aromatic agent in beer	quercetin (IC$_{50}$= 54.63± 3,16% at 200 μM), rutin (IC$_{50}$=61.87±5.48% at 200 μM), kaempferol (IC~~50~~=50.75±3.78% at 200 μM) and isorhamnetin (IC$_{50}$=50.75±3.78% at 200 μM), β-sitosterol (no inhibition) daucosterol (no inhibition)	[81]
Ononis spinosa L.<br>*(Restharrow roots)*<br>*(Fabaceae)*	Inflammations of the urinary tract	Dickloromethane extract (IC$_{50}$=0.19 mg/ml)<br>Isolated subractions: (86%& and 92% at 1 mg/ml)<br>Sativanone (IC$_{50}$=150.70 μM at 250 μM)	[82]
Pothos scandens L.<br>*(Araceae)*	Skin disorders, asthma, cancer	Pothobanoside A (46.7% Hyal inhibition at 200 μM)	[73,83]
Phyllanthus muellerianus Exell (*Kuntze*) (Euphorbiaceae)	Healing agent against wounds and other infections<br>Aqueous extracts of the stem bark show antimicrobial character against Streptococcus and Clostridium species	Hydroalcoholic extract (1:1 v/v) (IC$_{50}$= 80 μg/ml of Hyal-1)<br>Hydroalcoholic extracts fractions: (57.1% and 66.5% inhibition of Hyal-1) Three hydroalcoholic subractions (94%, 100% and 84% Hyal-1 inhibition at a concentration of 1 mg/ml)<br>**Isolated constituents:**<br>Chebulanin (IC$_{50}$=132 μM)<br>Mucic acid (43.8% Hyal-1 inhibition at 250 μM)<br>Furosine isomers (21.3% Hyal-1 inhibition at 250 μM)<br>Quercetin rutinoside (21.3% Hyal-1 inhibition at 250 μM)<br>kaempferol (8.9% Hyal-1 inhibition at 250 μM)	[84,85]
Keiskea japonica<br>Lamiaceae)	Antioxidant, anti-inflammatoyry, antidiuretic properties	80% Acetone extract (IC$_{50}$=608 μg/ml)<br>**Isolated constituents:**<br>shimobashiric acid C (88.7% Hyal inhibition at 596 μM)<br>rosmarinic acid (86.5% Hyal inihibition 309 μM)<br>acacenin7-O-β-D-glucuropyranoside (86.5% Hyal inhibition at 267 μM)	[86]
Clethra barbinervis<br>(Lamiaceae)	Anti-inflammatory, anti-allergic, anti-aging properties	Aqueous extract (88.6% Hyal inhibition at 2.0 mg/ml)<br>**Isolated constituents:** epicatechin (IC$_{50}$= 0.94 mM)<br>**triterpene saponins**:<br>ryobusaponin B (IC$_{50}$=1.25 mM), ryobusaponin C (IC$_{50}$=0.68 mM)<br>hemsganoside B (IC$_{50}$ =0.82 mM)	[87]
Barathranthus nodiflorus	Antioxidant and anti-inflammatory properties, free radical scavengers	Ethanolic bark extracts (IC$_{50}$=42.31±2.00 %)	[88]
Diospyros ebenum	Antioxidant and anti-inflammatory properties, free radical scavengers	Ethanolic bark extracts (IC$_{50}$= 41.60±1.18 %)	[88]
Acronychia pedunculata	Antioxidant, antibacterial and anti-inflammatory properties, free radical scavengers	Ethanolic bark extract (IC$_{50}$= 36.60±1.02 %)	[88]
Flacourtia indica	Anti-inflammatory, antioxidant, diuretic properties, Treatment against rheumatism	Ethanolic plant extract (IC$_{50}$=36.67±2.23 %).	[88]

Prismatomeris tetrandra (Roxb.) K. Schum	Wounds, bronchitis, snakebites	Ursolic acid (IC_{50}=103.18±1.70 μM), 3β, 19, 23-trihydroxyurs-12-en-28-oic acid (286.95±10.28 μM) and 3β-acetylolean-12-en-28-oic acid (1466.5±2.37 μM).	[89]
Scilla scilloides Druce (Liliacease)	Medicinal agent for blood circulatory activation, dermal disorders, antidote, antimicrobial, anticancer	Ethyl acetate bulb extract (IC_{50}= 169 μg/ml)<br>**Homoisoflavones:**<br>Scillavone B (IC_{50}=748 μM)<br>3-(3, 4-Dihydroxybenzylidene)-5,7-dihydroxy-6-methoxy—chroman-4-one (IC50 =887 μM)	[90]
Cimicifuga Rhizoma (mixture of the Rhizomes of *Cimicifuga dahurica* and *C. heracleifolia*)	Antipyretic, analgesic, would healing agent	Cimicifugic acids 50% Hyal inhibition at <200 μM	[91,92]
Gaultheria procumbens L. (*Estern teaberry, checkerberry)* (Ericaceae)	Northern traditional treatment	Chloroform extract (IC_{50}=282.15±10.38 μg/ml) which was 1.3 time)<br>**Terpenoid constituents** oleanolic acid (10.11% and ursolic acid (28.82%)	[70–72]
Clitoria Ternatea L. (*Butterfly pea)* (Fabaceae)	Nervous system disorders (stress, anxiety, depression etc)	Methanolic (IC_{50} =18.08 ± 0.46 μg/ml)<br>Ethyl acetate (IC_{50} =28.01 ± 0.48 μg/ml)<br>n-butanolic (IC_{50} =38.84 ± 0.41 μg/ml)	[18,19,93]
Takuran (Lamiaceae)	Menstrual disorder, menstrual cramps, cardiovascular diseases, anti-allergic agent	Clinopodic acid C (IC_{50}=80.1 μM), Lycopic acid A (IC_{50}=134 μM), Clinopodic acid E (IC_{50}=82.8 μM) and Lycopic acid B (IC_{50}=141 μM). Rosmarinic acid (IC_{50}=309 μM) Scizotenuin A (IC_{50}=241 μM).	[94]
Meehania urticifolia (*Makino*) (Lamiaceae)	Anti-inflammatory and antibacterial properties	Two isomers of rosmarinic acid (IC_{50}=275 μM and 183 μM)<br>Rosmarinic acid (IC_{50}=164 μM)	[95]
Carissa carandas (Apocynaceae)	Antipyretic, analgesic, anti-rheumatic, anti-inflammatory, anti-diabetic agent etc.	Steroid fraction of the plant's extract (IC_{50} = 5.19 mM)	[96]
Triphala guggulu (*Combination of three fruits: Phyllanthus emblica (amalaki or TI), Termilaia chebula (haritki or T2) and Terminalia belerica (bibhitaki or T3)*	Wound healings, ear-nose-throat system disorders	Hydroalcoholic extracts: (84.60±8.71%) of Hyal at a concentration of 4 mg/ml)<br>Aqueous extract: (85% Hyal inhibition at 0.10 mg/ml)<br>Separate constituents:<br>***P. emblica (T1)*** (100% Hyal inhibition at 0.30 mg/ml)<br>***T. chebula (T2)*** (100% Hyal inhibition at 15 mg/ml)<br>**T. belleirca (T3)** (no efficient Hyal inhibition)<br>(T1): (T2): (T3) 1:1:1 (100% Hyal inhibition at 0.30 mg/ml)	[97]
Eleutherococcus Maxim. Genus	Medicinal agents, dietary agents	Species: E. *gracilistylus* (IC_{50}= 16.4±0.05%),<br>*E. giraldii* (IC_{50}= 60.7±0.01%)<br>*E. senticocus* (IC_{50}=57.5±0.05%)	[80]
Eisenia bicyclis (*Brown alga)*	Antioxidant agent, food agent	8,8'- bieckol (IC_{50}=40 μM)<br>Dieckol (IC_{50}= 120 μM)<br>Phlorofucofuroeckol A (IC_{50}= 140 μM)<br>Acetylated derivatives of 8,8'-bieckol (IC_{50}= 15.1%)	[59,98]

Clinopodium gracile (Lamiaceae)	Anti-inflammatory, antitumor, antihyperglycemic properties, anti-hyaluronidase agent	Clinopodic acid J (IC_{50}=206 μM), Clinopodic acid K (IC_{50}=63 μM), Clinopodic acid L (IC_{50}=26 μM), Clinopodic acid M (IC_{50}=19 μM), Clinopodic acid N (IC_{50}=161 μM), Clinopodic acid O (IC_{50}=66 μM), Clinopodic acid P (IC_{50}=25 μM), Clinopodic acid Q (IC_{50}=165 μM), Rosmarinic acid (IC_{50}=226 μM), Clinopodic acid I (IC_{50}=112 μM), Clinopodic acid E (IC_{50}=40 μM), 8-epiblechnic acid (IC_{50}=653 μM) Lithospermic acid (IC_{50}=36μM), Salvianolic acid B (IC_{50}=107 μM), Salvianolic acid A (IC_{50}=206 μM), Cosmosiin (IC_{50}>1000 μM) apigenin-7-O-(6-O-malonyl)glucoside1 (IC_{50}=360 μM) apigenin-7-O-rutinoside (IC_{50}>1000) apiin (IC_{50}=533 μM) luteolin-7-O-glucoside (IC_{50}=695 μM) luteolin-7-O-(6-O-malinyl)glucoside (IC_{50}=324 μM) naringenin-7-O-rutinoside (IC_{50}>1000 μM)	[99,100,101,102]
Canavalia gladiate DC (Red sword beans of no fermentation)	Anti-inflammatory and antioxidant properties	IC_{50}=35.64±0.44% Hyal inhibition at a concentration of 5 mg/ml IC_{50}=45.73±0.78% Hyal inhibition at a concentration of 10 mg/ml IC_{50}=76.08±0.12% Hyal inhibition at a concentration of 25 mg/ml	[73]
Canavalia gladiate DC (Red fermented sword beans)	Anti-inflammatory and antioxidant properties	IC_{50}=39.28±0.59% Hyal inhibition at a concentration of 5 mg/ml IC_{50}=46.64±1.18% Hyal inhibition at a concentration of 10 mg/ml IC_{50}=77.37±0.19% Hyal inhibition at a concentration of 25 mg/ml	[73]

Table S4. Studied natural secondary metabolites for their inhibitory potency towards Hyaluronidase.

Inhibitor	***Chemical Family***	***Source***	***IC_{50}***	***Ref.***
Glycyrrhizin	Triterpenes		IC_{50}=0.440 mM, Hyal B *(Streptococcus agalactiae)* inhition IC_{50}=0.455 mM rHyal B (recombinant Hyal from *S. agalactiae*) inhibition	[59]
Glycyrhetinic acid	Triterpenes		IC_{50}=0.060 mM Hyal B *(Streptococcus agalactiae)* inhibition IC_{50}=0.080 mM rHyal B (recombinant Hyal from *S. agalactiae*) inhibition	[59]
Gypsophila saponin 2	Trieterpenoid Saponin glucosides		IC_{50}=108 μM Human Hyal-1	[79]

SA1657	Trieterpenoid Saponin glucosides		IC_{50}= 371 μM Human Hyal-1	[79]
SA1641	Trieterpenoid Saponin glucosides		IC_{50}=177 μM Human Hya;-1	[79]
Glycyrrhizinic acid	Triterpenes		IC_{50}=177 μM Human Hual-1 inhibition	[79]
β-caryophyllene	Essential oils	*Melaleuca leucadendron* Linn. Essential oils extract	IC_{50}= 4.17 μg/ml	[103]
1.8-cineol	Essential oils	*Melaleuca leucadendron* Linn Essential oils extract	IC_{50}=1.17 mg/ml	[103]
Naringenin	Flavonoids		IC_{50}=9.58±0.25% at 200 μM	[104]
7-O-tert-butoxycarbonylmethyl naringenin	Flavonoid derivatives		IC_{50}=30.68±0.21% at 200 μM	[104]
7-O-butyl naringenin	Flavonoid derivatives		IC_{50}=44.84±0.28% at 200 μM	[104]
7-O-(a-methoxycarbonyl)benzyl naringenin	Flavonoid derivatives		IC_{50}=5.80±0.13%% at 200 μM	[104]
7-O-(BnO-L-Leu-carbonylmethyl) naringenin	Flavonoid derivatives		IC_{50}=18.72±0.43% at 200 μM	[104]
liquiritigenin	Flavanone	*Glycyrrhiza glabra*	Weak Hyal inhibition (IC_{50}= 740 μM)	[105]
			IC_{50}=680±43 μmol/L	
isoliquiritegin	Flavanone	*Glycyrrhiza glabra*	Potent Hyal inhibition (IC_{50}= 64 μM)	[106]
Baicalein	Flavone	*Scutellaria baicalensis*	Low Hyal inhibition (IC_{50}=165 μM)	[107]
paeniflorin	Phenolic derivative	*Paeonia albiflora*	Potent Hyal inhibition	[107]

3. Activity of Tyrosinases

Tyrs have a double enzymatic activity, in the presence of O_2: **1)** they can act as monophenolases (cresolases) and hydroxylate monophenols in their ortho-position and **2)** they can act as diphenolases (catecholases) and oxidize *o*-diphenols into *o*-quinones. Tyrs prefer substrates with L- stereochemistry, like L-DOPA and L-tyrosine. The main reaction of Tyr is the oxidation of L-tyrosine into dopaquinone, a product which leads to the formation of eumelanin (brown or black melanin) and pheomelanin (red to yellow melanin). The enzyme's inhibitory activity is utilized in various ways: **a)** Reduction of the intermediate *o*-dopaquinone to dopa. There are a lot of reducing agents and the most common is ascorbic acid; **b)** Synthesis of *o*-dopaquinone derivatives, which produce colorless products, when they react with dopaquinone. For example, alkyl thiols can interact with dopaquinone; **c)** Use of substrates which form products unable to continue the reaction; **d)** Use of acidic, basic or other Tyr inhibitors which interrupt its function.[108]

3.1. Biological activity of Tyrosinases[109]

Melanin is formed through various steps of cyclic reactions and oxidative polymerizations.[110,111] The formation of melanin demands both the presence of the amino acid

tyrosine and Tyr.[112,113] In a first step, tyrosine is transformed into dihydroxyphenylalanine (DOPA) by Tyr, followed by a transformation to dopaquinone. In human cells, dopaquinone is autoxidized to dopachrome through dopachrome tautomerase, and then to 5,6-dihydroxyindole (DHI) or dihydroxyindole-2-carboxylic acid (DHICA) through DHICA oxidase, leading to the production of the brown-black pigment eumelanin. If there is cysteine or glutathione, dopaquinone is transformed to cysteinyl DOPA or glutathione DOPA, leading to the formation of the red-yellow pigment, pheomelanin.[110,113,114]

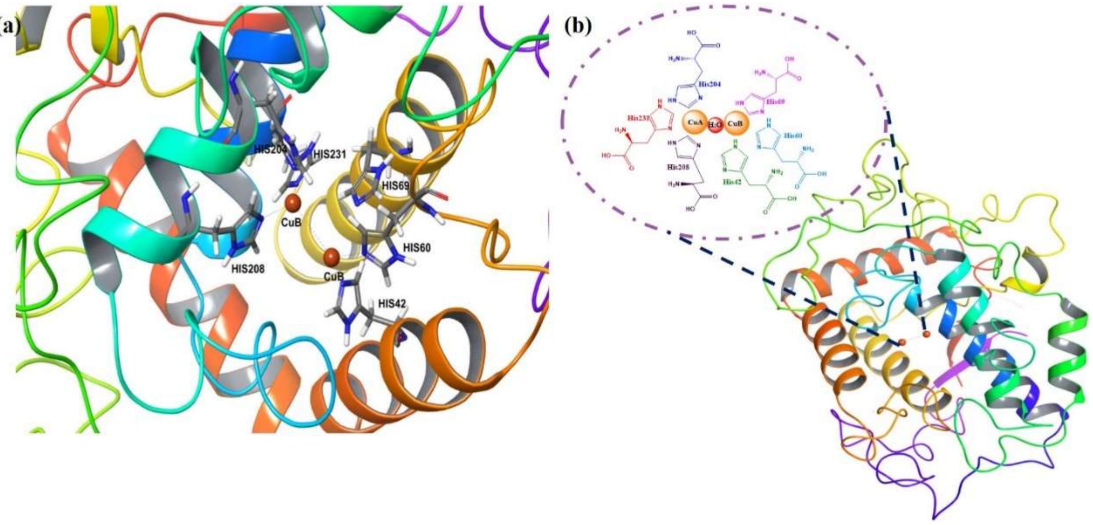

Figure S4. (a) Structure of Tyrosinase (PDB ID: 3NM8), indicating the catalytic sited amino acids. **(b)** Illustration of Tyrosinase's catalytic sited amino acids (PDB ID: 3NM8).

3.2. The importance of the number of –OH towards Tyrosinase inhibition

According to many studies, the number and the position of –OH groups in the aromatic ring of a compound is the main regulator of the rate of Tyr inhibition. Three flavanol glucosidic compounds were found to show Tyr inhibitory activity of different values: (2S, 3S)-2,3-*trans*-dihydromorin-7-*O*-β-D-glucoside with two –OH groups at 2' and 4' positions of B ring showed the most potent inhibitory activity, dihydrokaempferol-7-*O*-β-D-glucoside with one –OH group at 4' position of B ring showed strong inhibitory activity and taxifolin- 7-*O*-β-D-glucopyranoside with two –OH groups at 3' and 4' positions of B ring showed weaker inhibitory activity than the previous two glucosides. Two flavanones were found to show strong Tyr inhibitory activity: *trans*-dihydromorin and steppogenin with two –OH groups at 2' and 4' positions of B ring, whereas taxifolin, taxifolin-7-methyl ether with two –OH at 3' and 4' in B ring and dihydrokaempferol and naringenin with one -OH at 4' B ring showed weak Tyr inhibitory activity. On the contrary, previous studies showed that taxifolin is an efficient Tyr inhibitor,[115,116] whereas other studies reported that it can show potent oxidation activity.[117,118] Comparing steppogenin to *trans*-dihydromorin, the most potent inhibition activity belonged to steppogenin (IC_{50}=2.52 μM), with a double bond in C ring. Moreover, the compounds (2S,3S)-2,3-trans-dihydromorin-7-*O*-β-D-glucoside, *trans*-dihydromorin, dihydrokaempferol-7-*O*-β-D-glucoside and dihydrokaempferol with a glucosylated C-7 at ring A, were found to be weak Tyr inhibitors.[119] Two glycosylated isoflavones, orobol-8-C-glucoside and sphaerobioside showed more Tyr inhibitory activity than their non-glycosylated forms. Moreover, the presence of an –OMe group at C-7 and an isoprenyl group at 6 or /and 8 position at A ring or 5' position at B-ring enhanced their inhibitory activity, as for the isoflavones and isoprenylisoflavones: orobol, genistein, santal, glycyrrhisoflavone, wighteone, and 6, 8-

diprenylorobol. These results were different from previous studies.[120] Additionaly, in this study xanthone derivatives like physcion, 1, 5-dihydroxy-3, 6-dimethoxy-xanthen-9-one, cudraxanthone H and dulxanthone-B showed weak Tyr inhibitory activity. On the contrary, another study showed that physcion had the same value of Tyr inhibition activity, with kojic acid.[121] Furthermore the results showed that the presence of –OH groups at the positions 2 and 4 of an aromatic ring, increase the Tyr inhibitory activity, as among the 28 compounds, the strongest Tyr inhibitors found to be: (2S,3S)-2,3-trans-dihydromorin-7-O-β-D-glucoside, trans-dihydromorin, oxyresveratrol, steppogenin and quercetin.

In addition, studies on the isolated quercetin aglycone from the plant, Heterotheca inuloides[122], has been reported to have potent mushroom Tyr inhibitory activity at a concentration of 0.7 mM (IC_{50}=22 μg/mL), whereas its glucosides showed no significant anti-Tyr potency. This ensures the fact that a free –OH group at C-3 plays major role in the inhibition of Tyr. Similar studies on the isolated quercetin derivatives from the plant Cudrania cochinchinensis,[119] confirmed the fact the following glycosylated compounds in C-3 potistion showed lower Tyr inhibitory activity: quercetin-7-O-b-D-glucoside (IC_{50}= 143.0 mM) and quercetin- 3, 7-di-O-b-D-glucoside (IC_{50} > 1000 mM).[121] Structure characterization reported that quercetin interacts with the active site of mushroom Tyr, preventing the binding with L-DOPA, whereas the sugar moiety in the glucosides does not allow the interaction with the active site of the enzyme.

The living organisms have the potency of creating sun-light protecting ways, and, thus, a lot of new compounds are developed. Many studies,[124] identified the presence of (-)-*N*-formylanonaine, when they isolated the extract form ***Michelia alba***, and proposed it as Human Tyr inhibitor, as well as an antioxidant. Its anti-Tyr activity was stronger against Human Tyr than mushroom Tyr. In addition, the authors supported that (-)-**N**-formylanonaine was not cytotoxic. According to mechanistic studies, it has been reported that the interaction was achieved through bonds in the enzyme's active site, between the ligands and the Cu^{2+} ions. Another study[125] made on extracts from ***Castanea ehnryi***, reported that they contain lignin glycosides, like 2,3-dihydro-2-[4-(β)-glucopyranosyl, (1→2)-[β-glucopyranosyl-(1→6)]-β-glucopyranosyloxy)-3-methoxyphenyl]-3-(hydroxymethyl)-7-methoxy-5-benzofuranpropanol, when tested towards mushroom Tyr activity, were found to behave as *o*-diphenolase inhibitors similar to that of kojic acids. Another study[126] made on extracts from stem barks of ***Acer buergerianum*** showed that their phytochemicals contain compounds like 3-*O*-demethylnikoenoside, an aromatic glycoside, and 11 other compounds which were tested for their melanogenesis-inhibitory activities in *α*-melanocyte stimulating hormone (*α*-MSH)-stimulated B16 melanoma cells, and 3-*O*-demethylnikoenoside showed similar activity with that of kojic acid. Additionally, another study made on extracts from ***Artocarpus obtusus***,[127] reported that ***A. obtusus*** phytochemicals contain three xanthone derivatives from which pyranocycloartobiloxanthone A showed similar mushroom Tyr inhibitory activity to kojic acid.

Table S5. Plants, extracts and isolated compounds that have been studied for their inhibitory properties towards Tyrosinase.

Plant	*Medicinal use*	*Inhibition towards Tyr*	*Ref.*
Morus australis	Antioxidant, anti-inflammatory, anticancer properties, treatment against postprandial hypoglycemic disorders, anti-tyrosinase agent, cosmetics and skin-	**Isolated chalcones**:<br>(E)-1,3-bis(2,4-dixydroxyphenyl)1-prop-2-en-1-one (1) (IC_{50}=0.21 μM)<br>(E)- 1-(2,4-dihydroxy-3-(30methylbut-2-en-1-yl)phenyl)-3-(2,4-dihydroxyphenyl) prop-2-en-1-one (2) (IC_{50}=0.82 μM)<br>(1'R, 2'R, 3''R)-2'-(2,4-dihydroxy-3-(3-methylbut-2-en-1-yl)benzyl)-3''- (E)-(2,4-dixydroxyphenyl)-1-hydroxyallyl-5'-methyl-1',2',3'',6'-tetrahydro-[1,1',3'',1''-terphenyl]-2,2'',4,6''-	[128,129]

	whitening agent, food agent, production of wine and vinegar	tetraol (3) (IC_{50}=4.62 μM) (E)-1-(2,4-dihydroxy-3-((Z)-4-hydroxy-3-methylbut-2-en-prop-2-en-1-one (4) (IC_{50}=0.17 μM)	
Gastrodia elata	Treatment against neurodegenerative disorders, paralysis, stroke, dementia, vertigo and epilepsy	Bis-(4-hydroxybemzyl) sulfide (IC_{50}=0.53 μM, competitive inhibition)	[128]
Cassia fistula (Fabacee) (Golden shower)	Antioxidant, anticancer, antibacterial, antifungal, antidiabetic, Treatment against skin disorders	Flower extract: (IC_{50}=50-200 μg/ml)	[130–133]
Pyracantha fortunea	Digestive properties, cosmetic and skin-whitening agent	A (3,3'-dihydroxy-5'-methoxy-(1,1'-biphenyl)-4-O-β-D-glucoside<br>B (4'-hydroxy-2,3',5'-trimethoxy-(1,1'-biphenyl)-2'-O-β-D-glucoside<br>C (4'-hydroxy-3,5'-dimethoxy-(1,1'-biphenyl)-2-O-β-D-glucoside<br>D (2,4'-dihydroxy-3-5'-dimethoxy-(1,1'-biphenyl)-3-O-β-D-glucoside (IC_{50}=0.07 mM)<br>E 3,4'-dihydroxy-3',5'-dimethoxy-(1,1'-biphenyl)-4-O-β-D-glucoside	[134]
Crataegus pinnatifida (Hawthorn) (Rosaceae)	Medicinal agent, skin treatment, cosmetic agent, food agent	A: 8-O-4'-neolignan-9'-glucopyranoside (37.58% Tyr inhibition at 500 μg/ml)<br>B: (7R,8S)-erythro-3,7,3'-trimethoxy-8-O-4'-neolignan-9'-O-β-D glucopyranoside (known as pinnatifidaninside B) (34.54% Tyr inhibition at 500 μg/ml)<br>C: pinnatifidaninside C (31.5% Tyr inhibition at 500 μg/ml)<br>D: pinnatifidaninside D (32.97% Tyr inhibition at 32.97%)<br>E: 7R,8S-dihydrodehydrodiconiferyl alcohol-9-O-β-D-glucoside (46.00% Tyr inhibition at 500 μg/ml)<br>F:7R,8S-dihydrodehydrodiconiferyl alcohol-9'-O-β-D-glucoside (58.15% Tyr inhibition at 500 μg/ml)	[135,136]
Humulus Lupulus	Sleep disorders, restlessness, excitability promotion, digestive agent, treatments against spasma, cough, fever, inflammation, earache, toothache, food agent	n-Hexane extract (no Tyr inhibition)<br>Acetone extract (no Tyr inhibition)<br>Methanol-1 extract (no Tyr inhibition)<br>Methanol-2 extract (no Tyr inhibition)<br>Methanol-3 extract (no Tyr inhibition)<br>25% aqueous ethanol extract (no Tyr inhibition)	[137]
Artocarpus xanthocarpus Merr. (Moraceae)	Free radical scavenging and antityrosinase propeties	artoxanthol (IC_{50}=5.7±0.3 μM, mixed type competitive inhibition)<br>alboctalol (IC_{50}=6.4±0.3 μM, mixed type competitive inhibition)<br>steppogenin[140] (IC_{50}=1.9±0.1 μM) (competitive inhibition)<br>norartocarpetin (IC_{50}=0.9±0.1 μM, competitive inhibition)<br>resveratrol (IC_{50}=4.9±0.3 μM) oxyresveratrol (IC_{50}=1.0±0.5 μM) (non-competitive inhibition) chlorophorin (IC_{50}=2.5±0.4 μM)<br>artoxanthocarpuone A (IC_{50}=59.3±3.7 μM, mixed type competitive inhibition)	[138,139,140]

		hydroxylakoochin A (IC_{50}=97.5±1.5 μM)<br>artoxanthochromate (IC_{50}=85.8±0.1 μM)<br>morusin (IC_{50}=75.0±4.1 μM)<br>albanin A (IC_{50}=58.2±5.1 μM)<br>cudraflavone C (IC_{50}=40.8±1.9 μM)	
Malus doumeri (Formosan Apple) (Rosaceae)	Antioxidant agent, HNE inhibitior, Matrix Metalloproteinase inhibitor, Tyrosinase inhibitor	phloretin (IC_{50}=28.99±3.57% Human tyrosinase inhibition),<br>phloridzin (IC_{50}=11.32±2.34% Human tyrosinase inhibition,<br>3-hydroxyphloridzin (IC_{50}=22.53±2.33% Human tyrosinase inhibition),<br>Quercetin (IC_{50}=35.84±2.94% Human tyrosinase inhibition),<br>chrysin (IC_{50}=22.96±5.63% Human tyrosinase inhibition),<br>chrysin-5-glucoside (IC_{50}=16.64±2.84% Human tyrosinase inhibition),<br>3-hydroxyphloretin (IC_{50}=80.50±1.40% Human tyrosinase inhibition, cellular Tyrosinase inhibition: IC_{50}=32 μM),<br>protocatechuic acid (IC_{50}=33,45±1.59% Human tyrosinase inhibition),<br>catechol (IC_{50}=78.13±0.47 % Human tyrosinase inhibition, cellular tyrosinase inhibition: 22 μM),<br>rutin<br>(IC_{50}=16.94±2.31% Human tyrosinase axctivity), pynosylvin (IC_{50}=31.85±1.92% Human tyrosinase inhibition)	[53,141]
Cinnamomum osmophloeum Kanehira	Antioxidant, anti-inflammatory and antibacterial properties, flavoring and food agent	Plant extracts: (medium inhibition of mushrooum Tyr at 200 μM)	[142]
Xanthium strumarium L.(Xanthii fructus) (Asteraceae)	Leucoderma, fever, headache	Ethyl acetate extract (IC_{50}=0.26 mg/ml)<br>Protocatechuic acid (IC_{50}=2.53±0.06 mM, competitive inhibition), chlorogenic acid (IC_{50}=1.05±0.06 mM, mxed-type inhibition), 3,5-di-O-caffeoylquinic acid (IC_{50}=1.07±0.08 mM, competitive inhibition), 1,5-di-O-caffeoylquinic acid (IC_{50}=1.19±0.03 mM, competitive inhibition), 1,3-di-O-caffeoylquinic acid (IC_{50}=1.67±0.08 mM, mixed-type inhibition), 1,3,5-tri-O-caffeoylquinic acid (IC_{50}=1.16±0.06 mM, mixed type inhibition)	[143]
Metasequoia glyptostroboides	Antioxidant, antibacterial, antifungal and antidermatophytic properties	Taxiquinone (52.32% Tyr inhibition at 1000 μg/ml)	[144]
Koompassia malaccensis	Anticiabetic, antioxidant, antimalarial, antidysentery and antifever properties	Taxifolin, flavanol rhamnosides (5.86-25.9% myshroom Tyr inhibition)	[145]
Aloe	Anti-inflammatory, anti-viral, anti-bacterial, anti-cancer anti-diabetic, anti-allergy properties, cosmetics agent,		[146–149]

	health drinks and beverages agent		
Chloranthus tianmushanensis	Anti-tyrosinase agent	Terpenoids extracted from leaves (potent Tyr inhibition in a dose dependent manner)	[150]
Heterothea inuloides (Arnica)	Skin disorders	Plant extracts (IC_{50}= 190 µg/ml)<br>Quercetin (IC_{50}= 22 µg/ml)<br>Kaempferol (IC_{50}= 67 µg/ml)	[151]
Buddleia coriacea (Logariaceae)	Antimelanogenic properties	Buddlenoid A (IC_{50}= 0.39 mM)<br>Buddlenoid B (IC_{50}= 0.44 mM)	[152]
Dillenia indica (Elephant apple) (Dilleniaceae)	Antitumour agent, flavoring agent	Betulinic acid (Monophenolase inhibitory activity at 80 µM, diphenolase inhibitory activity at 40 µM, non-competitive inhibitor)	[153]
Calceolaria talcana (Calceolariaceae)	Diureticm antimicrobial agent	Ethyl acetate extract (IC_{50}=97.7 µgml)<br>**Isolated constituents:**<br>verbascoside (IC_{50}=108.4 µM, competitive inhibitor)<br>martynoside (IC_{50}=177.7 µM, competitive inhibitor)<br>naphthaquinone (IC_{50}= 91.2 µM, competitive inhibitor)<br>quercetin (IC_{50}=50 µM, competitive inhibitor)<br>benzoic acid (IC_{50}=640 µM, mixed type inhibitor)<br>tannic acid (IC_{50}=22 µM, competitive inhibitor)	[154]
Berberis Aristata (Berberidaceae)	Hepatoprotective, antidiarrhoeal, cardiotonic, antidiabetic, antimicrobia, anticancer, anti-inflammatory agent	Methanolic extract (97% monophenolase inhibition at 110 µg/ml, competitive inhibition) (50% diphenolase inhibition at 412, 01 µg/ml, mixed type inhibition)<br>Aqueous extract (78% monphenolase inhibition at 110 µg/ml, competitive inhibition)50% diphenolase inhibition at 431.11 µg/ml, mixed tipe inhibition)	[155]
Polygonum cuspidatum (Polygonaceae)	Antibacterial, antioxidant, anti-inflammatory agent	Supeercritical carbon dioxide fruit extract (<10.0% mushroom Tyr inhibition at 20 µg/ml, <10.0% inhibition at 50 µg/ml, 14.8±1.23% inhibition at 100 µg/ml, 22.6±1.61% inhibition at 250 µg/ml)	[121,156,157]
Cudrania cochinchinensis	Rheumatism, hepatitis, gonorrhea, bruising, constuted wounds	95% Ethanolic extract (IC_{50}=36.3 µg/ml)<br>Root extract (IC_{50}=56.2 µg/ml)<br>Twig extract (IC_{50}>400 µg/ml)<br>Leaf extract (IC_{50}>400 µg/ml)<br>Isolated compounds:<br>oxyresveratrol (IC_{50}=2.33±0.24 µM), 2, 3-trans-dihydromorin (IC50=21.09±0.70 µM)<br>2, 3-cis-dihydromorin (IC_{50}=31.14±0.49 µM).<br>quercetin-7-O-β-D-glucoside (IC_{50}=143.037±2.16 µM),<br>kaempferol 7-O-β-D-glucopyranoside (IC_{50}>100 µM)<br>morin-7-O-β-D-glucoside<br>(IC_{50}= 196.33±4.47 µM)<br>quercetin-7-O-b-D-glucoside (IC50= 143.0 mM) and quercetin- 3, 7-di-O-b-D-glucoside (IC_{50} > 1000 mM)<br>kaempferol-7-O-b-glucopyranoside (low inhibition)<br>kaempferol-3,7-di-O-b-glucopyranoside (low inhibition),<br>dihydrokaempferol-7-O-b-D-qlucopyranoside (low inhibition)<br>aromadendrin (low inhibition)	[119,158]
Artocarpus heterophyllus	Antioxidant, anti-inflammatory, antiaging and	Artocarpfuranol (IC_{50} <50 µM), dihydromorin (IC_{50} <50 µM), steppogenin (IC_{50} <50 µM), norartocarpetin (IC_{50} <50 µM),	[120,138,159,160]

	antimelanogenic agent, food agent	artocarpanone (IC_{50} <50 μM), artocarpesin (IC_{50} <50 μM), and isoartocarpesin (IC_{50} <50 μM)	
Campylotropis hirtella (Legumisae)	Amenorrhea, metrorhagia, metrostaxis, gastric ulcers, benign prostate hyperplasia, food ingredient	Methanolic root barks extract (IC_{50}=60% at 20 μg/ml)<br>3'-geranyl-5,7,2',4'-tetrahydroxyisoflavanone (subs: L-tyrosine: IC_{50}=2.9±0.3 μM, subs: L-DOPA: IC_{50}=128.2±0.5 μM, competitive inhibition, with both substrates),<br>3'-geranyl-5,7,3',5'-tetrahy-droxyisoflavone (subs: L-tyrosine: IC_{50}=92.0±0.2 μM, subs: L-DOPA: IC_{50}>200 μM, competitive inhibition with L-DOPA as substrate),<br>Neuroflavane (subs: L-tyrosine: IC_{50}=0.03±0.006 μM, subs: L-DOPA: IC_{50}=0.5±0.03 μM, competitive inhibition with both substrates),<br>(E)-3-(3-(3,7-dimethylocta-2,6-dienyl)-2,4-dihydroxyphenyl)-3,5,7-trihydroxy-chroman-4-one (subs: L-tyrosine: IC_{50}=18.4±0.8 μM, subs: L-DOPA: IC_{50}=144.0±1.2 μM, competitive inhibition with both substrates)	[24,25,27,161]
Flemingia philippinensis	Antioxidant, anti-inflammatory, cytotoxicity, antiestrogenic, immunosuppressive properties, food agent	Root methanolic extract (80% Tyr inhibition at 30 μg/ml)<br>fleminchalcone A (**subs. L-tyrosine:** IC_{50}= 1.01 μM, subs. L-DOPA: IC_{50}=19.5 μM, monophenolase and diphenolase inhibitory activity, competitive inhibition)<br>fleminchalcone B (**subs. L-tyrosine:** IC_{50}=18.4 μM, **subs. L-DOPA:** IC_{50}=32.6 μM, monophenolase and diphenolase inhibitory activity competitive inhibition)<br>fleminchalcone C (**subs. L-tyrosine:** IC_{50}= 1.28μM, (**subs. L-DOPA:** IC_{50}= 5.22 μM, monophenolase and diphenolase inhibitory activity, competitive inhibition)<br>flemichin D (**subs. L-tyrosine:** IC_{50}= 1.79 μM, subs. L-DOPA: IC_{50}=7.48 μM, monophenolase and diphenolase inhibitory activity, competitive inhibition)<br>lupinifoin, (**subs. L-tyrosine:** IC_{50}= 11.2 μM, subs. L-DOPA: IC_{50}=84.10 μM, monophenolase and diphenolase inhibitory activity, competitive inhibition)<br>khonklonginol H (**subs. L-tyrosine:** IC_{50}= 4.96 μM, subs. L-DOPA: IC_{50}=20.4 μM, monophenolase and diphenolase inhibitory activity, competitive inhibition)	[41,129,162,163]
Herniaria glabra L.	Hypotension, antispasmodic and diuretic properties, treatments against urinary tract infections, cystitis, irritable bladder, skin disorders	Pure crude extract (7.39±0.59% Tyr inhibition at 1 mg/ml)<br>Saponin fraction (8.50±1.40% Tyr activity at 1 mg/ml)<br>bidesmoside herniaria saponin 8 (4.21±0.79% Tyr inhibition at 1 mg/ml)<br>bidesmoide herniaria saponin 10 (8.44±1.69% Tyr inhibition at 1 mg/ml)<br>bidesmoside herniaria saponin 11 (8.25±1.17% Tyr inhibition at 1 mg/ml)<br>bidesmoside herniaria saponin 12 (7.73±1.04% Tyr inhibition at 1 mg/ml)<br>bidesmoside herniaria saponin 13 (IC_{50}= 9.82±1.35% Tyr inhibition at 1 mg/ml)<br>monidesmoside herniaria saponin 16 (IC_{50}= 7.30±1.22% Tyr inhibition at 1 mg/ml)	[16]

		monodesmoside herniaria saponin 17 (IC_{50}= 3.64±0.72% Tyr inhibition at 1 mg/ml)<br>bidesmoside herniaria saponin 1 (IC_{50}= 8.77±1.27% Tyr inhibition at 1 mg/ml)<br>monodesmoside herniaria saponin 4 (IC_{50}= 3.39±1.69% Tyr inhibition at 1 mg/ml)<br>bidesmoside herniaria saponin 5 (IC_{50}= 9.75±1.53% Tyr inhibition at 1 mg/ml)<br>monodemoside hrniaria saponin 6 (IC_{50}= 2.18±0.97% Tyr inhibition at 1 mg/ml)<br>monodesmoside herniaria saponin 7 (IC_{50}= 8.40±0.50% Tyr inhibition at 1 mg/ml)	
Rhizophora mucrinata L. (Rhizophoraceae)	Main source of carbon, vitamins, proteins, minerals, fatty acids, energy for humans and living organisms, climate change regulator	Methanolic twig extract (IC_{50}= 145.31±1.39 mg KAE (kojic acid equivalent/g)<br>Methanolic leaf extract (IC_{50}=±144.02 mg KAE (kojic acid equivalent/g)	[38,164]
Eucalyptus globulus Labill (Timber tree)	Flu, rheumatism, dysentery, eczema	**Ethanolic extract isolated compounds:**<br>**isoiphionane sesquiterpene:**<br>3β,11-dihydroxyisoiphion-4-one (IC_{50}= 14.17 μM)<br>5-formyl-4-hydroxy-2-isopropyl-7-methylbenzofuran-6-O-β-D-glucopyranoside (known as eucalglobuide A) (IC_{50}= 57.08± 2.52 μM)<br>5-formyl-6-hydroxy-2-isopropyl-7-mthylbenzofuran (IC_{50}= 91.76± 3.41 μM)<br>4-O-β-D- glucopyranoside (eucalglobuside B) chromene glucoside (IC_{50}=49.16 ±0.12 μM)<br>5β, 11-dihydroxy-iphionan-4-one (IC_{50}= 10.08 μM)<br>Proximadiol (IC_{50}> 100 μM)<br>(-)-α- eudesmol (IC_{50}> 100 μM)<br>(-)-globulol (IC_{50}=9.79 μM)<br>4β, 10 α-aromadendranediol (IC_{50}> 100 μM) vomifoliol (IC_{50}> 100 μM)<br>Isololiolide (IC_{50}> 100 μM)<br>Eucalyptin (IC_{50}= 33.43±0.14 μM)<br>(+)-rhododendrol (IC_{50}=42.63±0.43 μM)<br>4-(4'-hydroxy-3'-methoxyphenyl)-2R-butanol (IC_{50}= 21.65 μM)<br>ursolic acid lactone (IC_{50}> 100 μM)<br>3β-acetoxyurs-11-en-28 13 olide, pinoresinol (IC_{50}= 74.57 ±0.26 μM).<br>2,5-dimethylhydroquinone (IC_{50}> 100 μM)	[165]
Mangifera indica L. (Mango) (Anacardiaceae)	Diabetes, respiratory disorders, antimicrobial, anti-osteoporosis, andi-cardiovascular agent, the aqueous leaves extracts are consumed as tea	Ethyl acetate extract (IC_{50}= 17.62±1.26 μg/ml)<br>n-butanol extract (IC_{50}= 117.84±9.62 μg/ml)<br>Aqueous extract (IC_{50}=557.92±27.18 μg/ml)<br>**Major inhibitors:** gallic acid, mangiferin, protocatecuic acid, hyperoside, quercitrin, quercetin-3-O-xyloside, derivatives pf benzophenone, epicatechin gallate, 1,2, 3, 4, 6-penta-O-galloy glucoside, luteolin-7-O-glucoside, kaempferol-3-O-glucoside, quercetin-3-O-rhamnoside	[166]

		**Minor inhibitors:** Isomangniferin, 6'-O-(p-hydroxybenzoyl) mangiferin, glycosidic derivatives of irriflophenone such as iriflophenone_3-C-(2',3',6'-tri-O-galloyl)-glucoside, glucosidic derivatives of maclurin [3-C-(2'-O-galloyl)-glucoside and maclurin 3-C-(2',3'-di-O-galloyl)-glucoside]	
Camellia Pollen	Antitoxic, anti-inflammatory, antioxidant, antimutagrnic agent, food supplement	Caffeine (IC_{50}=18.5±2.31 µg/ml, reversible, noncompetitive inhibition, Ki=80 µM)<br>Baicalein (IC_{50}= 21.7 µg/ml)<br>Brazilein (IC_{50}= 6.07 mg/ml)<br>Thobarbituric acid (IC_{50}=1.15 mg/ml)	[167]
Malcolmia littorea (L).	Anti-inflammatory, antioxidant agents, use for pharmaceutical, food and cosmetic applications	Methanolic root extract (IC_{50}= 24.96±0.19 mg KAE/g)<br>ethanolic root extract (IC_{50}=25.32±0/04 mg KAE/g) aqueous root extract (IC50= 6.28±0.45 mg KAE/g)<br>ethanolic aerial organ extract (IC_{50}= 25.78±0.18 mg KAE/g)<br>methanolic extract (IC_{50}= 26.48 ±0.12 mg KAE/g)<br>aqueous (IC_{50}= 5.32 ±0.08 mg KAE/g)<br>flower ethanolic extract (IC_{50}= 26.56±0.23 mg KAE/g)<br>methanolic (IC_{50}= 25.85±0.21 mg KAE/g)<br>aqueous (IC_{50}= 4.33±0.39 mg KAE/g)	[168]
Morinda morindoides (Baker) (Rubiaceae)	Hemorrhoids, rheumatism, gonorrhea, malaria, diarrhea, amebiasis	Aqueous seed extract (IC_{50}= 24.56±0.69 mg KAE/g)<br>Aqueous fruit extract (IC_{50}= 43.70±1.26 mg KAE/g)<br>Methanolic seeds extract (IC_{50}= 72.40±0.46 mg KAE/g)<br>Methanolic fruit extract (IC_{50}= 73.59±1.24 mg KAE/g)	[169–172]
Cakile Maritina Scop. (Sea rocket) (Brassicaceae or mustard)	Scurvy, digestive disorders, diuretic disorders, dandruff, food agents for flavor improvement (leaves), bread making (ground roots)	Aerial organs ethanolic extract (IC_{50}=25.9±0.13 mg/ml)<br>Aerial organs acetone extract (IC_{50}=24.7±0.13 mg/ml)<br>Aerial organs aqueous extract (IC_{50}= 19.9±0.12 mg/ml)<br>Fruit ethanolic extract (IC_{50}= 24.9±0.25 mg/ml)<br>Fruit acetone extract (IC_{50}= 24.0±0.33 mg/ml)<br>Fruit aqueous extract (IC_{50}= 6.16±0.30 mg/ml)	[173–175]
Leonurus japonicas (Yi Mu Cao) (Labiatae)	Dysmenorrhea, menoxenia, amenorrhea, ulcerations etc	10- methoxy-leonurine (IC_{50}= 91.8 ±2.9% Tyr inhibition at 100 µM, competitive inhibition (Ki= 1.6±0.7 µM)<br>Leonurine (IC_{50}= 85.6±1.8% Tyr inhibition at 100 µM, competitive inhibition, Ki=11.4±1.1 µM)<br>syringic acid (IC_{50}= 11.6±0.1% Tyr inhibition at 100 µM)<br>isouercitrin (IC_{50}= 1.8±5.9% Tyr inhibition at 100 µM)<br>leonurusoide E (IC_{50}= 8.3±0.6% Tyr inhibition at 100 µM)	[176,177]
Grapes	Wine production	Caftaric acid (IC_{50}= 30 µM)<br>Chlorogenic acid (IC_{50}= 42 µM)<br>Caffeic acid (IC_{50}= 65 µM)	[178]
Wulfenia Carinthiaca s.L. (National flower of Carinthia) (Plantaginaceae)	Ornamental plant, cosmetic agent	Aerial part methanolic extract (40% mushroom Tyr inhibition at 500 µg/ml)<br>**Methanolic extract isolated compounds:**<br>**Iridoid glucosides**:<br>plantmamajoside (IC_{50}= 0.11±3.61% mushroom Tyr inhibition at 500 µM), globularicisin (cis-globularin, (4.20±6.06% mushroom Tyr inhibition at 500 µM)<br>2'-O-Acetylplantamajoside (IC_{50}= 33.07±1.00 % mushroom Tyr inhibition at 500 µM),<br>globularin (79.59±1.62 % mushroom Tyr inhibition at 500 µM, IC_{50}= 41.94 µM)	[179]

		**Phenylethanoid glucosides:**<br>2',6''-O-Diacetylplantamajoside (IC_{50}= 29.76±4.24 % mushroom Tyr inhibition at 500 μM)<br>2'-O-Acetylisoplantamajoside (IC_{50}= 13.50±3.10 % mushroom Tyr inhibition at 500 μM)<br>baldaccioside (IC_{50}= 23.01±3.16 % mushroom Tyr inhibition at 500 μM), isoscrophularoside (IC_{50}= 48.49±2.08% mushroom Tyr inhibition at 500 μM) 2',6''-O-diacetylisoplantamajoside (IC_{50}= 26.14±3.18% mushroom Tyr inhibition at 500 μM)	
Neolentinus lepideus (Fr.) (Redhead and Ginns) (lentinus lepideus (Fr.) (Gloeophyllaceae)	Antimicrobial properties, cosmetic agents against melanoma, food intake (for edible mushrooms)	Culture filtrate extracts (72% Tyr inhibition at 1000 μg/ml)<br>**Isolated compounds:**<br>1, 3-dihydroisobenzofuran-4,5,7-triol (IC_{50}= 173 μg/ml, competitive inhibition)<br>5-methoxy-1,3-dihydroisobenzofuran-4,7-diol (IC_{50}= 263 μg/ml, competitive inhibition)	[180]
Asplenium trichomanes (Aspleniaceae)	Antitumour, antioxidant and antidiabetic properties	Aerial parts methanolic extract<br>4-ethylphenyl-6-O-96-deoxy-α-L-mannopyranosyl)-β-D-glucopyranoside (IC_{50}≥600 μM)	[181]
Scutellaria altissima (Lamiaceae)	Haemostatic, tonic, wound healing properties, food and beverage agent	Aerial parts methanolic extract<br>Globularin (IC_{50}=41.91 μM)	[181]
Pinus uncinata subsp. Uncinata (Pinaceae)	Antiseptic, astringent, diuretic, antispasmotic properties	Methanolic extract<br>Benzoic acid (IC_{50}≥551.53 μM)<br>Roseoside (IC_{50}≥1200 μM)<br>Dihydrovomifoliol-O-β-D-glucopyranoside (IC_{50}≥1200 μM)	[181]
Puearariae Lobatae Radix	Anti-diabetixc, anti-fever, anti-diarrheal aget, skin-whitening	Puerarin (IC_{50}=0.537 mg/ml, monophenolase activity, mixed-type inhibitor/ diphenolase activity: (Ka)- 1.45 mg/ml, mixed-type activation mechanism)	[182–184]
Pueraria thumbergiana (Kudzu) (Leguminosae)	Anti-inflammatory, anti-diabetic, anti-cardiovascular, anti-liver steatosis, anti-melanogenic, antipyretic, analgesic, muscle relaxant agent	Aerial part (potent mushroom tyrosinase inhibition)<br>Plant extracts (potent cellular tyrosinase inhibition in B16F10 cells, after stimulation with α-MSH)	[182–185]
Pueraria lobate Ohwi	Anti-inflammatory, antioxidant, anti-cardiovascular, antidiabetic agent etc	Purarin (45%-76% Tyr inhibition at a range of 0.5-8.0 mg/ml, IC_{50}= 1.23 mmol/L)	[182–185]
Vigna angularis	Hepatoprotective, anticancer, anti-inflammatory, antioxidant agent, food agent	Seeds extracted condensed tannins: (IC_{50}=130.0≥0.5 μg/ml, monophenolase inhibition, IC_{50}=35.10±2.0 μg/ml, diphenolase inhibition, mixed-type reversible mushroom tyrosinase inhibition)	[186]
Clausena lansium	Antidiabetic, anticancer and antioxidant properties	Plants extracted condensed tannins (IC_{50}=23.6±0.3 μg/ml, monophenolase inhibition)	[162,187]
Haworth	Antimicrobial, antioxidant properties	Fruit stone extracted condensed tannins (IC_{50}=37.00 ±05 μg/ml, monophenolase inhibition)	[188]

Avogado	Antioxidant and antifungal properties	Fruit stone extracted condensed tannins (IC_{50}=40.00±1.2 μg/ml, monophenolase inhibition)	[188]
Cudrania tricuspidata	Treatment against digestive apparatus tumor, anti-inflammatory, antifungal, anti-lipid peroxidative, α-glucosidase, antioxidative and cytotoxic properties	(2S,3S)-2,3-trans-dihydromorin-7-O-β-D-glucoside (IC_{50}= 93.17±1.55 μM)<br>taxifolin- 7-O-β-D-glucopyranoside (IC_{50}> 200 μM)<br>protocatechuic acid (IC_{50}> 500 μM) sphaerobioside (IC_{50}> 150 μM)<br>orobol-8-C-glucoside (IC_{50}> 200 μM)<br>dihydrokaempferol-7-O-β-D-glucoside (IC_{50}> 200 μM)<br>taxifolin (IC_{50}> 300 μM)<br>trans-dihydromorin (IC_{50}=21.54±0.84 μM)<br>oxyresveratrol (IC_{50}=2.85±0.26 μM)<br>dihydrokaempferol (IC_{50}> 100 μM) taxifolin 7-methyl ether (IC_{50}> 300 μM)<br>steppogenin (IC_{50}=2.52±0.66 μM) quercetin (IC_{50}=54.58±0.89 μM)<br>orobol (IC_{50}> 300 μM)<br>naringenin (IC_{50}> 500 μM)<br>genistein (IC_{50}> 300 μM)<br>santal (IC_{50}> 300 μM)<br>glycyrrhisoflavone (IC_{50}> 200 μM) wighteone (IC_{50}> 100 μM)<br>6,8-diprenylorobol (IC_{50}> 100 μM) 1,5-dihydroxy-3,6-dimethoxy-xanthen-9-one (IC_{50}> 300 μM) cudraxanthone H (IC_{50}≥ 200 μM)<br>alpinumisoflavone (IC_{50}> 200 μM)<br>8-(γ,γ-dimethylallyl)wighteone (IC_{50}> 200 μM)<br>dulxanthone-B (IC_{50}> 200 μM) cyclomorusin (IC_{50}> 200 μM)<br>5-methoxy-4,5-diphenyl-2(5H)-furanone (IC_{50}> 300 μM)<br>cycloaltilisin-7 (IC_{50}> 200 μM)	[189–191]
Green tea	(EGCG), (-) epigallocatechin (EGC). (-)-epicatechin (EC), (+)-catechin (C), caffeine (CAF)	(-)-epicatechin 3-O-gallate (ECG) (IC_{50}= 34.58 μM)<br>(-)-gallocatechin 3-O-gallate (GCG), (IC_{50}= 17.34 μM, competitive inhibition)<br>(-)epigallocatechin 3-O-gallate (EGCG) (IC_{50}=34.10 μM)	[192]
Dillenia indica		Triterpenoid	[153]
Glycyrrhiza species (Leguminosae)- Glycyrrhiza glabra	Skin-whitening agent	Glabridin (potentt tyrosinase inhibition)<br>Glabrene (potent tyrosinase inhibition)	[193]
Glycyrrhiza species (Leguminosae)- Glycyrrhiza uralensis	Skin-whitening agent	Ethyl acetate fraction from methanolic extract:<br>(Flavone) Licoisoflavone A (I_{50}> 100 μg/mL)<br>Coumarin (Glycycoumarin) (IC_{50}> 100 μg/mL)<br>Flavanone (3'-(γ, γ'- dimethylallyl)-kievitone (IC_{50}> 100 μg/mL)<br>Isoflavone (glycyrrhisoflavone) ic50=46.2±0.60 μg/mL, Anti-melanogenic activity on B16F10 melanoma cells (IC50= 63.7±6.8% at a concentration of 5 μg/mL)<br>Flavanone: Glyasperin C-3 (IC_{50}=0.13 ±0.01 μg/mL)<br>Flavanone: Glabridine C-5 (IC_{50}=0.25 μg/mL)	

Table S6. Studied natural secondary metabolites for their inhibitory properties towards Tyrosinase.

Inhibitor	*Chemical Family*	*Source*	*IC_{50}*	*Binding Properties*	*Ref.*
Kaempferol	Flavonoids				[123,151]
Quercetin	Flavonoids				[151,194]

Kuarinone	Flavonoids			[109]
Kushnol F	Flavonoids			[109]
Luteolin 4′-O-glucoside	Flavonoid glucosides			[168]
Luteolin 7-O-glucoside	Flavonoid glucosides			[168]
Morin	Flavonoid			[195]
Catechin	Flavonoid			[196]
Rhamnetin	Flavonoid		30.6% murine Tyr inhibition on B16 cells at 5 μM, 63.3% murine Tyr inhibition on B16 cells at 20 μM and 75.5% murine Tyr inhibition on B16 cells at 40 μM.	[197]
Gallic acid	Phenolic acids			[192]
1,2,3,4,6-Penta-O-galloyl-d-glucose (PGG)	Gallic acid derivative	Galla rhois	Strong inhibition	[109,196,198]
198(S)-N-trans-Feruloyloctopamine	Phenolic acid derivatives	Garlic skin	IC_{50}=5.3±1.8 μM	[199]
(+) catechin	Tannins	Green tea	IC_{50}=57.12 μM	[196]
(-)-epicatechin gallate (ECG)	Tannins	Green tea	IC_{50}=22.63 μM	[196]
(-)-epigallocatechin-3-O-gallate (EGCG)	Tannins	Green tea	IC_{50}=142.40 μM	[196]
β-arbutin	(hydroquinone β-D-glucopyranoside)		Potent Tyr inhibition, used as cosmetic agent	[200]
Deoxyarbutin	Synthetic hydroquinone derivative		Potent Tyr inhibition, used as cosmetic agent	[108,162,201]
Mequinol	Hydroquinone monomethyl ether		Potent Tyr inhibition, used as cosmetic agent	[202]
Licochalcone A	Chalcone	Glycyrrhiza species	Pontent mushroom Tyr inhibitor	[108,193]
Kuraridin	Chalcone			[203]
Kuraridinol	Chalcone			[203,204]
2,4, 2′, 4′-tetrahydroxy-3-(3-methyl-n-butenyl) chalcone	Chalcone		Potent Tyr inhibition	[128,162,193]
Resveratrol	Stilbenes		Strong Tyr inhibition (32 times higher Tyr inhibition than standard control kojic acid)	[121,205]
Trans-cinnamaldehyde	Aldehyde derivatives			[109,206]
(2E)-alkenals	Aldehyde derivatives			[109,196,206]
2-hydroxy-4-methoxybenzaldehyde	Aldehyde derivatives			[207,208]
Anisaldehyde	Aldehyde derivatives			[208,209]
Cuminaldehyde	Aldehyde derivatives			[210,211]

Cumic acid	Aldehyde derivatives			[210]
3,4-dihydroxycinnamic acid	Cinnamic acid derivatives			[209]
4-hydroxy-3-methoxycinnamic acid	Cinnamic acid derivatives			[209]
Glycolic acid		Grapes, sugarcane, beets	IC_{50}=83.00±14.00 µM 98.5% tyrosinase inhibition at a concentration of 200 µM, mixed-type reversible inhibition	[212] [213]

4. Software development: the ANTI-AGE Database

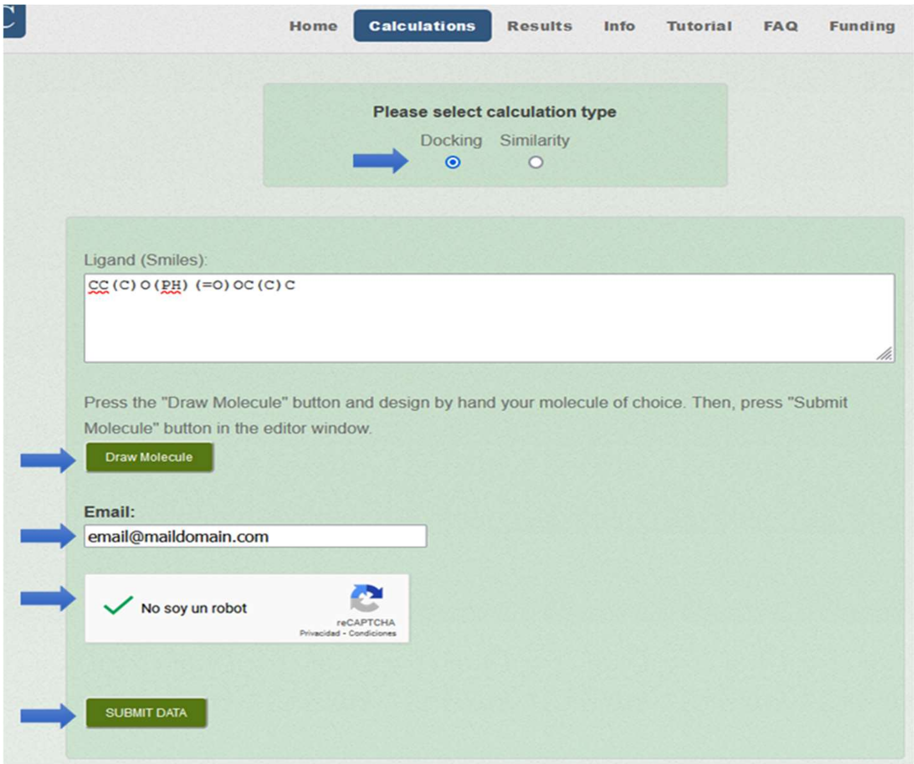

Figure S5. Representation of the first step of the determination of calculations in ANTIAGE-DB, by inserting the respective SMILES of the studied compound.

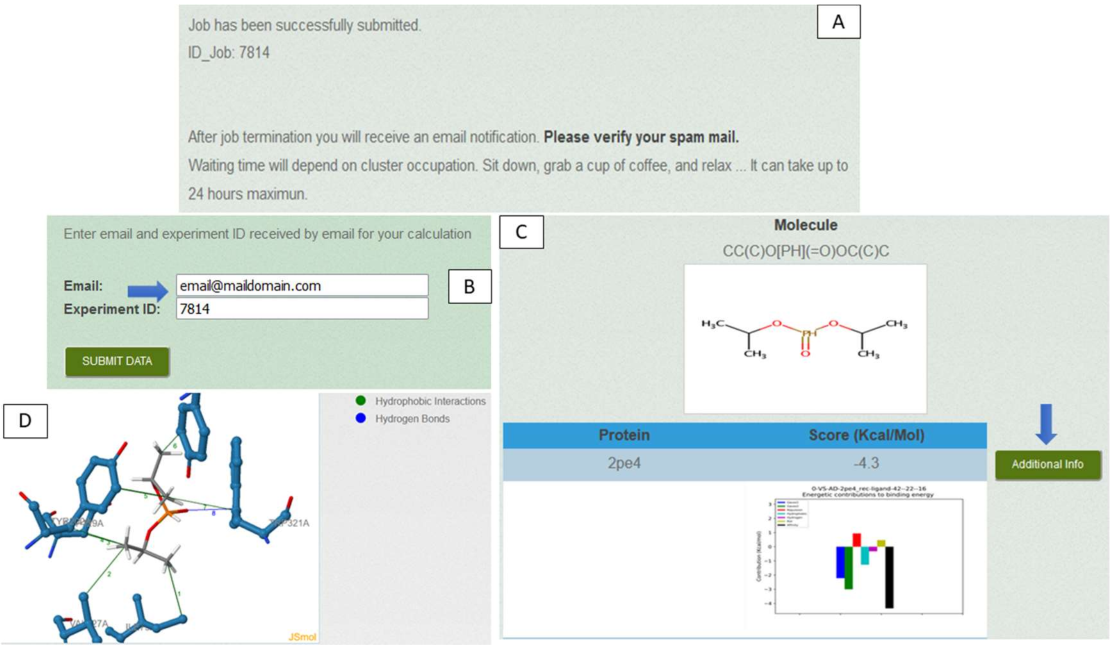

Figure S6. Detailed representation of the whole procedure for the submission of calculations in the ANTIAGE-DB. The respective arrows are explained in **Section C.2.3**.

Table S7. Illustration of the results given with the two softwares (NC-DB and Maestro) a) Elastase with caffeic acid, b) Hyaluronidase with quercetin and c) Tyrosinase with betulinic acid.

a) Elastase-Caffeic acid

NC-DB RESULTS			MAESTRO RESULTS		
Hydrophobic Interactions	Hydrogen Bonds	pi-Stacking	Hydrophobic Interactions	Hydrogen Bonds	pi-Stacking
PHE192	PHE41	PHE192	PHE192	VAL41	HIS57
	GLY193			GLY193	
	VAL216			LEU216	

b) Hyaluronidase-Quercetin

NC-DB RESULTS			MAESTRO RESULTS		
Hydrophobic Interactions	Hydrogen Bonds	pi-Stacking	Hydrophobic Interactions	Hydrogen Bonds	pi-Stacking
GLU131	GLU131	TYR202	TYR202	GLU131	TYR247
TYR202	TYR202	TYR261	TYR247	ASP292	ARG134
TYR247	GLY203	ARG265		ARG134	ARG265
	TYR210	ASP292			
	SER245				

c) Tyrosinase-Betulinic acid

NC-DB RESULTS				MAESTRO RESULTS			
Metal Complexes	Metal Complexes	Hydrophobic Interactions	Hydrogen Bonds	Metal Complexes	Metal Complexes	Hydrophobic Interactions	Hydrogen Bonds
HIS42	HIS204	PHE197	VAL218	42 HIS	204 HIS	VAL217	ASN205
HIS60	HIS208	HIS231	PRO201	60 HIS	208 HIS	PHE197	

	**ASN205**		**231 HIS**	**ASN205**
	**VAL217**			**VAL218**
	**VAL218**			
	**PRO219**			

Table S8. Illustration of the results given with the two softwares (NC-DB and Maestro) for the compound Auricoulasin[42], potent Elastase inhibitor.

**NC-DB RESULTS**			**MAESTRO RESULTS**		
**Hydrophobic Interactions**	**Hydrogen Bonds**	**pi-Stacking**	**Hydrophobic Interactions**	**Hydrogen Bonds**	**pi-Stacking**
**LEU143**	**PHE41**	**HIS57**	**143 LEU**	**41 PHE**	**57 HIS**
**PHE192**	**SER195**		**192 PHE**	**195 SER**	
	**SER214**		**41 PHE**	**214 SER**	

REFERENCES

1. Hess, G.P.; McConn, J.; Ku, E.; McConkey, G. Studies of the activity of chymotrypsin. *Philos. Trans. R. Soc. Lond. B. Biol. Sci.* **1970**, *257*, 89–104.
2. Tamada, T.; Kinoshita, T.; Kurihara, K.; Adachi, M.; Ohhara, T.; Imai, K.; Kuroki, R.; Tada, T. Combined high-resolution neutron and X-ray analysis of inhibited elastase confirms the active-site oxyanion hole but rules against a low-barrier hydrogen bond. *J. Am. Chem. Soc.* **2009**, *131*, 11033–11040, doi:10.1021/ja9028846.
3. Siedle, B.; Hrenn, A.; Merfort, I. Natural compounds as inhibitors of human neutrophil elastase. *Planta Med.* **2007**, *73*, 401–420, doi:10.1055/s-2007-967183.
4. Lee, W.L.; Downey, G.P. Leukocyte elastase: physiological functions and role in acute lung injury. *Am. J. Respir. Crit. Care Med.* **2001**, *164*, 896–904, doi:10.1164/ajrccm.164.5.2103040.
5. Henderson, R. Structure of crystalline alpha-chymotrypsin. IV. The structure of indoleacryloyl-alpha-chyotrypsin and its relevance to the hydrolytic mechanism of the enzyme. *J. Mol. Biol.* **1970**, *54*, 341–54.
6. Tsai, Y.-F.; Hwang, T.-L. Neutrophil elastase inhibitors: a patent review and potential applications for inflammatory lung diseases (2010 - 2014). *Expert Opin. Ther. Pat.* **2015**, *25*, 1145–58, doi:10.1517/13543776.2015.1061998.
7. Wolfram Bode, Edgar Meyer Jr., J.C.P. Perspectives in Biochemistry. Human Leukocyte and PorcinePancreatic Elastase: X-ray Crystal Structures,. Mechanism, Substrate Specificity . *Biochemistry* **1989**, *28*, 1951–1963.
8. Löser, B.; Kruse, S.O.; Melzig, M.F.; Nahrstedt, A. Inhibition of neutrophil elastase activity by cinnamic acid derivatives from Cimicifuga racemosa. *Planta Med.* **2000**, *66*, 751–753, doi:10.1055/s-2000-9563.
9. Feng, L.; Liu, X.; Zhu, W.; Guo, F.; Wu, Y.; Wang, R.; Chen, K.; Huang, C.; Li, Y. Inhibition of human neutrophil elastase by pentacyclic triterpenes. *PLoS One* **2013**, *8*, 1–11, doi:10.1371/journal.pone.0082794.
10. Thring, T.S.; Hili, P.; Naughton, D.P. Anti-collagenase, anti-elastase and anti-oxidant activities of extracts from 21 plants. *BMC Complement. Altern. Med.* **2009**, *9*, 27, doi:10.1186/1472-6882-9-27.
11. Maity, N.; Nema, N.K.; Abedy, M.K.; Sarkar, B.K.; Mukherjee, P.K. Exploring tagetes erecta linn flower for the elastase, hyaluronidase and MMP-1 inhibitory activity. *J. Ethnopharmacol.* **2011**, *137*, 1300–1305, doi:10.1016/j.jep.2011.07.064.
12. Xu, G.-H.; Kim, Y.-H.; Choo, S.-J.; Ryoo, I.-J.; Yoo, J.-K.; Ahn, J.-S.; Yoo, I.-D. Chemical constituents from the leaves of Ilex paraguariensis inhibit human neutrophil elastase. *Arch. Pharm. Res.* **2009**, *32*, 1215–1220, doi:10.1007/s12272-009-1905-7.
13. Nema, N.K.; Maity, N.; Sarkar, B.; Mukherjee, P.K. Cucumis sativus fruit-potential antioxidant, anti-hyaluronidase, and anti-elastase agent. *Arch. Dermatol. Res.* **2011**, *303*, 247–252, doi:10.1007/s00403-010-1103-y.
14. Battinelli, L.; Daniele, C.; Cristiani, M.; Bisignano, G.; Saija, A.; Mazzanti, G. In vitro antifungal and anti-elastase activity of some aliphatic aldehydes from Olea europaea L. fruit. *Phytomedicine* **2006**, *13*, 558–63, doi:10.1016/j.phymed.2005.09.009.
15. An, B.-J.; Kwak, J.-H.; Park, J.-M.; Lee, J.-Y.; Park, T.-S.; Lee, J.-T.; Son, J.-H.; Jo, C.; Byun, M.-W. Inhibition of Enzyme Activities and the Antiwrinkle Effect of Polyphenol Isolated from the Persimmon Leaf (Diospyros kaki folium) on Human Skin. *Dermatologic Surg.* **2006**, *31*, 848–855, doi:10.1111/j.1524-4725.2005.31730.
16. Kozachok, S.; Pecio, Ł.; Orhan, I.E.; Deniz, F.S.S.; Marchyshyn, S.; Oleszek, W. Reinvestigation of Herniaria glabra L. saponins and their biological activity. *Phytochemistry* **2020**, *169*, 112162, doi:10.1016/j.phytochem.2019.112162.
17. Moon, J.-Y.; Yim, E.-Y.; Song, G.; Lee, N.H.; Hyun, C.-G. Screening of elastase and tyrosinase inhibitory activity from Jeju Island plants. *EurAsian J. Biosci.* **2010**, *53*, 41–53, doi:10.5053/ejobios.2010.4.0.6.

18. Nema, N.K.; Maity, N.; Sarkar, B.K.; Mukherjee, P.K. Matrix metalloproteinase, hyaluronidase and elastase inhibitory potential of standardized extract of *Centella asiatica*. *Pharm. Biol.* **2013**, *51*, 1182–1187, doi:10.3109/13880209.2013.782505.
19. Mukherjee, P.; Maity, N.; Nema, N.; Sarkar, B. Standardized Clitoria ternatea leaf extract as hyaluronidase, elastase and matrix-metalloproteinase-1 inhibitor. *Indian J. Pharmacol.* **2012**, *44*, 584, doi:10.4103/0253-7613.100381.
20. Wittenauer, J.; Mäckle, S.; Sußmann, D.; Schweiggert-Weisz, U.; Carle, R. Inhibitory effects of polyphenols from grape pomace extract on collagenase and elastase activity. *Fitoterapia* **2015**, *101*, 179–187, doi:10.1016/j.fitote.2015.01.005.
21. Boje, K.; Lechtenberg, M.; Nahrstedt, A. New and Known Iridoid- and Phenylethanoid Glycosides from Harpagophytum procumbens and their in vitro Inhibition of Human Leukocyte Elastase. *Planta Med.* **2003**, *69*, 820–825, doi:10.1055/s-2003-43225.
22. Piwowarski, J.P.; Kiss, A.K.; Kozłowska-Wojciechowska, M. Anti-hyaluronidase and anti-elastase activity screening of tannin-rich plant materials used in traditional Polish medicine for external treatment of diseases with inflammatory background. *J. Ethnopharmacol.* **2011**, *137*, 937–941, doi:10.1016/j.jep.2011.05.039.
23. Tan, X.F.; Kim, D.W.; Song, Y.H.; Kim, J.Y.; Yuk, H.J.; Wang, Y.; Curtis-Long, M.J.; Park, K.H. Human neutrophil elastase inhibitory potential of flavonoids from Campylotropis hirtella and their kinetics. *J. Enzyme Inhib. Med. Chem.* **2016**, *31*, 16–22, doi:10.3109/14756366.2015.1118683.
24. Han HY, Wang XH, Wang NL, et al. Lignans isolated from Campylotropis hirtella (Franch.) Schindl. decreased prostate specific antigen and androgen receptor expression in LNCaP cell. *J Agric Food Chem* **2008**, *56*, 6928–35.
25. Li XP, Xuan BX, Shou QY, S.Z. New flavonoids from Campylotropis hirtella with immunosuppressive activity. *Fitoterapia* **2014**, *95*, 220–8.
26. Shou QY, Fu RZ, Tan Q, S.Z. Geranylated flavonoids from the roots of Campylotropis hirtella and their immunosuppressive activities. *J Agric Food Chem* **2009**, *57*, 6712–19.
27. Pientaweeratch, S.; Panapisal, V.; Tansirikongkol, A. Antioxidant, anti-collagenase and anti-elastase activities of Phyllanthus emblica, Manilkara zapota and silymarin: an in vitro comparative study for anti-aging applications. *Pharm. Biol.* **2016**, *54*, 1865–1872, doi:10.3109/13880209.2015.1133658.
28. Ma J, Luo XD, Protiva P, Yang H, Ma C, Basile MJ, W.; IB, K.E. Bioactive novel polyphenols from the fruit of Manilkara zapota (Sapodilla). *J Nat Prod.* **2003**, *66*, 983– 986.
29. Khurram, M.; Lawton, L.A.; Edwards, C.; Iriti, M.; Hameed, A.; Khan, M.A.; Khan, F.A.; ur Rahman, S. Rapid bioassay-guided isolation of antibacterial clerodane type diterpenoid from Dodonaea viscosa (L.) Jaeq. *Int. J. Mol. Sci.* **2015**, *16*, 20290–20307, doi:10.3390/ijms160920290.
30. Uddin, Z.; Li, Z.; Song, Y.H.; Kim, J.Y.; Park, K.H. Visconata: A rare flavonol having long chain fatty acid from Dodonaea viscosa which inhibits Human neutrophil elastase (HNE). *Tetrahedron Lett.* **2017**, *58*, 2507–2511, doi:10.1016/j.tetlet.2017.05.059.
31. Krenn, L.; Wollenweber, E.; Steyrleuthner, K.; Görick, C.; Melzig, M.F. Contribution of methylated exudate flavonoids to the anti-inflammatory activity of Grindelia robusta. *Fitoterapia* **2009**, *80*, 267–269, doi:10.1016/j.fitote.2009.03.001.
32. Stahl-Biskup E. Grindelia. In: BlaschekW, Hänsel R, Keller K, R.J.; Rimpler H, Schneider G, E. Hagers Handbuch der Pharmazeutischen Praxis, 5th ed. Folgeband 2: Drogen A-K. Berlin. *Springer* **1998**, 812.
33. Kim, J.Y.; Lee, J.H.; Song, Y.H.; Jeong, W.M.; Tan, X.; Uddin, Z.; Park, K.H. Human neutrophil elastase inhibitory alkaloids from chelidonium majus L. *J. Appl. Biol. Chem.* **2015**, *58*, 281–285, doi:10.3839/jabc.2015.044.
34. Saleem, M.; Nazir, M.; Hussain, H.; Tousif, M.I.; Elsebai, M.F.; Riaz, N.; Akhtar, N. Natural Phenolics as Inhibitors of the Human Neutrophil Elastase (HNE) Release: An Overview of Natural Anti-inflammatory Discoveries during Recent Years. *Antiinflamm. Antiallergy. Agents Med. Chem.* **2018**, *17*, 70–94, doi:10.2174/1871523017666180910104946.
35. Lee, S.M.; Song, Y.H.; Uddin, Z.; Ban, Y.J.; Park, K.H. Prenylated flavonoids from Epimedium Koreanum Nakai and their human neutrophil elastase inhibitory effects. *Rec. Nat. Prod.* **2017**, *11*, 514–520, doi:10.25135/rnp.66.17.05.090.
36. H. Ma, X. He, Y. Yang, M. Li, D.H. and Z.J. (2011). The genus Epimedium: an ethnopharmacological and phytochemical review. *J. Ethnopharmacol. 1* **2011**, *34*, 519–541.
37. Xu, G.H.; Ryoo, I.J.; Kim, Y.H.; Choo, S.J.; Yoo, I.D. Free radical scavenging and antielastase activities of flavon ids from the fruits of Thuja orientalis. *Arch. Pharm. Res.* **2009**, *32*, 275–282, doi:10.1007/s12272-009-1233-y.
38. Sadeer, N.B.; Rocchetti, G.; Senizza, B.; Montesano, D.; Zengin, G.; Uysal, A.; Jeewon, R.; Lucini, L.; Mahomoodally, M.F. Untargeted metabolomic profiling, multivariate analysis and biological evaluation of the true mangrove (Rhizophora mucronata lam.). *Antioxidants* **2019**, *8*, doi:10.3390/antiox8100489.
39. Zhang, J.; Xu, H.Y.; Wu, Y.J.; Zhang, X.; Zhang, L.Q.; Li, Y.M. Neutrophil elastase inhibitory effects of pentacyclic triterpenoids from Eriobotrya japonica (loquat leaves). *J. Ethnopharmacol.* **2019**, *242*, 111713, doi:10.1016/j.jep.2019.01.037.
40. Cha, D.S., Eun, J.S., Jeon, H. Anti-inflammatory and antinociceptive properties of the leaves of Eriobotrya japonica. *J. Ethnopharmacol.* **2011**, *134*, 305–312.
41. Kim, J.Y.; Wang, Y.; Uddin, Z.; Song, Y.H.; Li, Z.P.; Jenis, J.; Park, K.H. Competitive neutrophil elastase inhibitory isoflavones from the roots of Flemingia philippinensis. *Bioorg. Chem.* **2018**, *78*, 249–257, doi:10.1016/j.bioorg.2018.03.024.
42. M. Chen, S.Q. Luo, J.H.C. Studies on the chemical constituents of Flemingia philippinensis. *Acta Pharm. Sin.* **1991**, *26*, 42–48.
43. H. Li, M. Yang, J. Miao, X.M. Simultaneous chromatographic fingerprinting and quantitative analysis of Flemingia philippinensis by LC–DAD. *Chromatographia* **2009**, *70*, 447–454.

44. Melzig, M.F.; Henke, K. Inhibition of thrombin activity by selected natural products in comparison to neutrophil elastase. *Planta Med.* **2005**, *71*, 787–789, doi:10.1055/s-2005-871253.
45. Xing, X.; Yang, X.; Cao, Y. Study of Ellagic Acid as a Natural Elastase Inhibitor by Spectroscopic Methods. *J. Appl. Spectrosc.* **2016**, *83*, 149–155, doi:10.1007/s10812-016-0259-4.
46. Steinbrecher, T.; Case, D.A.; Labahn, A. A multistep approach to structure-based drug design: Studying ligand binding at the human neutrophil elastase. *J. Med. Chem.* **2006**, *49*, 1837–1844, doi:10.1021/jm0505720.
47. Filip, R.; López, P.; Giberti, G.; Coussio, J.; Ferraro, G. Phenolic compounds in seven South American Ilex species. *Fitoterapia* **2001**, *72*, 774–8.
48. Tatefuji, T.; Izumi, N.; Ohta, T.; Arai, S.; Ikeda, M.; Kurimoto, M. Isolation and identification of compounds from Brazilian propolis which enhance macrophage spreading and mobility. *Biol. Pharm. Bull.* **1996**, *19*, 966–70.
49. Góngora, L.; Giner, R.M.; Máñez, S.; Recio, M. del C.; Schinella, G.; Ríos, J.L. Effects of caffeoyl conjugates of isoprenyl-hydroquinone glucoside and quinic acid on leukocyte function. *Life Sci.* **2002**, *71*, 2995–3004.
50. Choi, S.Z.; Lee, S.O.; Choi, S.U.; Lee, K.R. A new sesquiterpene hydroperoxide from the aerial parts of Aster oharai. *Arch. Pharm. Res.* **2003**, *26*, 521–5.
51. Hrenn, A.; Steinbrecher, T.; Labahn, A.; Schwager, J.; Schempp, C.M.; Merfort, I. Plant phenolics inhibit neutrophil elastase. *Planta Med.* **2006**, *72*, 1127–1131, doi:10.1055/s-2006-946700.
52. Sartor, L.; Pezzato, E.; Garbisa, S. (-)Epigallocatechin-3-gallate inhibits leukocyte elastase: potential of the phyto-factor in hindering inflammation, emphysema, and invasion. *J. Leukoc. Biol.* **2002**, *71*, 73–9.
53. Leu, S.J.; Lin, Y.P.; Lin, R.D.; Wen, C.L.; Cheng, K.T.; Hsu, F.L.; Lee, M.H. Phenolic constituents of Malus doumeri var. formosana in the field of skin care. *Biol. Pharm. Bull.* **2006**, *29*, 740–5.
54. Ram K., S.; Amit, R.; Munglu, M.; Vinay Kumar, D.; Jaya, D.; Arvind K., J. Review on skin aging and compilation of scientific validated medicinal plants, prominence to flourish a better research reconnoiters in herbal cosmetic. *Res. J. Med. Plant* 2013, *7*, 1–22.
55. Kacem, R.; Meraihi, Z. EŠects of Essential Oil Extracted from Nigella sativa (L.) Seeds and Its Main Components on Human Neutrophil Elastase Activity. *Yakugaku Zasshi* **2006**, *126*, 301–305, doi:10.1248/yakushi.126.301.
56. Ying, Q.L.; Rinehart, a R.; Simon, S.R.; Cheronis, J.C. Inhibition of human leucocyte elastase by ursolic acid. Evidence for a binding site for pentacyclic triterpenes. *Biochem. J.* **1991**, *277*, 521–526, doi:/pmc/articles/PMC1151264/.
57. Siedle, B.; Cisielski, S.; Murillo, R.; Lo, B.; Castro, V.; Klaas, C.A.; Hucke, O.; Labahn, A.; Melzig, M.F.; Merfort, I.; et al. Sesquiterpene Lactones as Inhibitors of Human Neutrophil Elastase. *Bioorg. Med. Chem.* **2002**, *10*, 2855–2861.
58. Stern, R.; Jedrzejas, M.J. Hyaluronidases: Their genomics, structures, and mechanisms of action. *Chem. Rev.* **2006**, *106*, 818–839, doi:10.1021/cr050247k.
59. Załuski, D.; Cieśla, Ł.; Janeczko, Z. Chapter 7 – The Structure–Activity Relationships of Plant Secondary Metabolites with Antimicrobial, Free Radical Scavenging and Inhibitory Activity toward Selected Enzymes; 2015; Vol. 45; ISBN 9780444634733.
60. Chao, K.L.; Muthukumar, L.; Herzberg, O. Structure of human hyaluronidase-1, a hyaluronan hydrolyzing enzyme involved in tumor growth and angiogenesis. *Biochemistry* **2007**, *46*, 6911–20, doi:10.1021/bi700382g.
61. Machiah, D.K.; Girish, K.S.; Gowda, T.V. A glycoprotein from a folk medicinal plant, Withania somnifera, inhibits hyaluronidase activity of snake venoms. *Comp. Biochem. Physiol. C. Toxicol. Pharmacol.* **2006**, *143*, 158–61, doi:10.1016/j.cbpc.2006.01.006.
62. Leanpolchareanchai, J.; Pithayanukul, P.; Bavovada, R.; Saparpakorn, P. Molecular docking studies and anti-enzymatic activities of thai mango seed kernel extract against snake venoms. *Molecules* **2009**, *14*, 1404–1422, doi:10.3390/molecules14041404.
63. Marković-Housley, Z.; Miglierini, G.; Soldatova, L.; Rizkallah, P.J.; Müller, U.; Schirmer, T. Crystal structure of hyaluronidase, a major allergen of bee venom. *Structure* **2000**, *8*, 1025–1035, doi:10.1016/S0969-2126(00)00511-6.
64. Meyer, K. Hyaluronidases. In The Enzymes. *(New York Acad. Press.* **1971**, *3rd Editio*, 307–320.
65. Kreil, G. Hyaluronidases - a group of neglected enzymes. *Protein Sci.* **1995**, *4*, 1666–1669, doi:10.1002/pro.5560040902.
66. Nawy, S.S.; Csóka, A.B.; Mio, K.; Stern, R. Hyaluronidase activity and hyaluronidase inhibitors. Assay using a microtiter-based system. *Methods Mol. Biol.* **2001**, *171*, 383–9, doi:10.1385/1-59259-209-0:383.
67. Jedrzejas, M.J.; Stern, R. Structures of vertebrate hyaluronidases and their unique enzymatic mechanism of hydrolysis. *Proteins Struct. Funct. Bioinforma.* **2005**, *61*, 227–238, doi:10.1002/prot.20592.
68. Selenge, E.; Odontuya, G.; Murata, T.; Sasaki, K.; Kobayashi, K.; Batkhuu, J.; Yoshizaki, F. Phytochemical constituents of Mongolian traditional medicinal plants, Chamaerhodos erecta and C. altaica, and its constituents prevents the extracellular matrix degradation factors. *J. Nat. Med.* **2013**, *67*, 867–875, doi:10.1007/s11418-013-0748-1.
69. Selenge, E.; Murata, T.; Tanaka, S.; Sasaki, K.; Batkhuu, J.; Yoshizaki, F. Monoterpene glycosides, phenylpropanoids, and acacetin glycosides from Dracocephalum foetidum. *Phytochemistry* **2014**, *101*, 91–100, doi:10.1016/j.phytochem.2014.02.007.
70. Michel, P.; Owczarek, A.; Matczak, M.; Kosno, M.; Szymański, P.; Mikiciuk-Olasik, E.; Kilanowicz, A.; Wesołowski, W.; Olszewska, M.A. Metabolite profiling of eastern teaberry (Gaultheria procumbens L.) lipophilic leaf extracts with hyaluronidase and lipoxygenase inhibitory activity. *Molecules* **2017**, *22*, 1–16, doi:10.3390/molecules22030412.
71. Liu, W.-R.; Qiao, W.-L.; Liu, Z.-Z.; Wang, X.-H.; Jiang, R.; Li, S.-Y.; Shi, R.-B.; She, G.-M. Gaultheria: Phytochemical and Pharmacological Characteristics. *Molecules* **2013**, *18*, 12071–12108, doi:10.3390/molecules181012071.

72. Nikolic, M.; Markovic, T.; Mojovic, M.; Pejin, B.; Savic, A. P.; T.; Markovic, D.; Stevic, T.; Sokovic, M. Chemical Composition and Biological Activity of Gaultheria Procumbens L. Essential Oil. *Ind. Crop. Prod.* **2013**, *49*, 561–567.
73. Scotti, L.; Kumar Singla, R.; Mitsugu Ishiki, H.; Jaime B. Mendonca, F.; Sobral da Silva, M.; Maria Barbosa Filho, J.; Tullius Scotti, M. Recent Advancement in Natural Hyaluronidase Inhibitors. *Curr. Top. Med. Chem.* **2016**, *16*, 2525–2531, doi:10.2174/1568026616666160414123857.
74. Citalingam, K.; Zareen, S.; Shaari, K.; Ahmad, S. Effects of Payena dasyphylla (Miq.) on hyaluronidase enzyme activity and metalloproteinases protein expressions in interleukin-1β stimulated human chondrocytes cells. *BMC Complement. Altern. Med.* **2013**, *13*, 213, doi:10.1186/1472-6882-13-213.
75. Ippoushi, K.; Yamaguchi, Y.; Itou, H.; Azuma, K.; Higashio, H. Evaluation of Inhibitory Effects of Vegetables and Herbs on Hyaluronidase and Identification of Rosmarinic Acid as a Hyaluronidase Inhibitor in Lemon Balm (Melissa officinalis L.). *Food Sci. Technol. Res.* **2000**, *6*, 74–77.
76. Lee, K.K.; Cho, J.J.; Park, E.J.; Choi, J.D. Anti-elastase and anti-hyaluronidase of phenolic substance from Areca catechu as a new anti-ageing agent. *Int. J. Cosmet. Sci.* **2001**, *23*, 341–346, doi:10.1046/j.0412-5463.2001.00102.x.
77. Sendker, J.; Böker, I.; Lengers, I.; Brandt, S.; Jose, J.; Stark, T.; Hofmann, T.; Fink, C.; Abdel-Aziz, H.; Hensel, A. Phytochemical Characterization of Low Molecular Weight Constituents from Marshmallow Roots (Althaea officinalis) and Inhibiting Effects of the Aqueous Extract on Human Hyaluronidase-1. *J. Nat. Prod.* **2017**, *80*, 290–297, doi:10.1021/acs.jnatprod.6b00670.
78. González-Peña, D.; Colina-Coca, C.; Char, C.D.; Cano, M.P.; De Ancos, B.; Sánchez-Moreno, C. Hyaluronidase inhibiting activity and radical scavenging potential of flavonols in processed onion. *J. Agric. Food Chem.* **2013**, *61*, 4862–4872, doi:10.1021/jf3054356.
79. Orlando, Z.; Lengers, I.; Melzig, M.F.; Buschauer, A.; Hensel, A.; Jose, J. Autodisplay of human hyaluronidase Hyal-1 on Escherichia coli and identification of plant-derived enzyme inhibitors. *Molecules* **2015**, *20*, 15449–15468, doi:10.3390/molecules200915449.
80. Załuski, D.; Olech, M.; Kuźniewski, R.; Verpoorte, R.; Nowak, R.; Smolarz, H.D. LC-ESI-MS/MS profiling of phenolics from Eleutherococcus spp. inflorescences, structure-activity relationship as antioxidants, inhibitors of hyaluronidase and acetylcholinesterase. *Saudi Pharm. J.* **2017**, *25*, 734–743, doi:10.1016/j.jsps.2016.11.002.
81. Liu, M.; Yin, H.; Dong, J.; Xiao, L.; Liu, G.; Qian, Z.; Miao, J. Inhibition and interaction with hyaluronidase by compounds from hop (humulus lupulus l) flowers. *Asian J. Chem.* **2013**, *25*, 10262–10266, doi:10.14233/ajchem.2013.15260.
82. Addotey, J.N.; Lengers, I.; Jose, J.; Gampe, N.; Béni, S.; Petereit, F.; Hensel, A. Isoflavonoids with inhibiting effects on human hyaluronidase-1 and norneolignan clitorienolactone B from Ononis spinosa L. root extract. *Fitoterapia* **2018**, *130*, 169–174, doi:10.1016/j.fitote.2018.08.013.
83. Muhit, M.A.; Izumikawa, M.; Umehara, K.; Noguchi, H. Phenolic constituents of the Bangladeshi medicinal plant Pothos scandens and their anti-estrogenic, hyaluronidase inhibition, and histamine release inhibitory activities. *Phytochemistry* **2016**, *121*, 30–37, doi:10.1016/j.phytochem.2015.10.009.
84. Addotey, J.N.; Lengers, I.; Jose, J.; Hensel, A. Hyal-1 inhibitors from the leaves of Phyllanthus muellerianus (Kuntze) Excell. *J. Ethnopharmacol.* **2019**, *236*, 326–335, doi:10.1016/j.jep.2019.03.022.
85. Brusotti, G., Cesari, I., Frassà, G., Grisoli, P., Dacarro, C., Caccialanza, G. Antimicrobial properties of stem bark extracts from Phyllanthus muellerianus (Kuntze) Excell. *J. Ethnopharmacol.* **2011**, *135*, 797–800.
86. Murata, T.; Miyase, T.; Yoshizaki, F. Hyaluronidase inhibitors from Keiskea japonica. *Chem. Pharm. Bull.* **2012**, *60*, 121–128, doi:10.1248/cpb.60.121.
87. Murata, T.; Suzuki, A.; Mafune, N.; Sato, E.; Miyase, T.; Yoshizaki, F. Triterpene saponins from Clethra barbinervis and their hyaluronidase inhibitory activities. *Chem. Pharm. Bull.* **2013**, *61*, 134–143, doi:10.1248/cpb.c12-00566.
88. Perera, H.D.S.M.; Samarasekera, J.K.R.R.; Handunnetti, S.M.; Weerasena, O.V.D.S.J.; Weeratunga, H.D.; Jabeen, A.; Choudhary, M.I. In vitro pro-inflammatory enzyme inhibition and anti-oxidant potential of selected Sri Lankan medicinal plants. *BMC Complement. Altern. Med.* **2018**, *18*, 1–15, doi:10.1186/s12906-018-2335-1.
89. Abdullah, N.H.; Thomas, N.F.; Sivasothy, Y.; Lee, V.S.; Liew, S.Y.; Noorbatcha, I.A.; Awang, K. Hyaluronidase inhibitory activity of pentacylic triterpenoids from prismatomeris tetrandra (Roxb.) K. schum: Isolation, synthesis and QSAR Study. *Int. J. Mol. Sci.* **2016**, *17*, doi:10.3390/ijms17020143.
90. Nishida, Y.; Sugahara, S.; Wada, K.; Toyohisa, D.; Tanaka, T.; Ono, M.; Yasuda, S. Inhibitory effects of the ethyl acetate extract from bulbs of Scilla scilloides on lipoxygenase and hyaluronidase activities. *Pharm. Biol.* **2014**, *52*, 1351–1357, doi:10.3109/13880209.2014.891140.
91. Iwanaga, A.; Kusano, G.; Warashina, T.; Miyase, T. Hyaluronidase inhibitors from "cimicifugae Rhizoma" (a mixture of the rhizomes of cimicifuga dahurica and C. heracleifolia). *J. Nat. Prod.* **2010**, *73*, 573–578, doi:10.1021/np900675n.
92. Sakai, S.; Ochiai, H.; Nakajima, K.; Terasawa, K. Inhibitory effect of ferulic acid on macrophage inflammatory protein-2 production in a murine macrophage cell line, RAW264.7. *Cytokine* **1997**, *9*, 242–8, doi:10.1006/cyto.1996.0160.
93. Mukherjee, P.K.; Kumar, V.; Kumar, N.S.; Heinrich, M. The Ayurvedic medicine Clitoria ternatea--from traditional use to scientific assessment. *J. Ethnopharmacol.* **2008**, *120*, 291–301, doi:10.1016/j.jep.2008.09.009.
94. Murata, T.; Watahiki, M.; Tanaka, Y.; Miyase, T.; Yoshizaki, F. Hyaluronidase inhibitors from Takuran, Lycopus lucidus. *Chem. Pharm. Bull.* **2010**, *58*, 394–397, doi:10.1248/cpb.58.394.

95. Murata, T.; Miyase, T.; Yoshizaki, F. Hyaluronidase inhibitory rosmarinic acid derivatives from Meehania urticifolia. *Chem. Pharm. Bull.* **2011**, *59*, 88–95, doi:10.1248/cpb.59.88.
96. Patil, S.; Bhadane, B.; Shirsath, L.; Patil, R.; Chaudhari, B. Steroidal fraction of Carissa carandas L. inhibits microbial hyaluronidase activity by mixed inhibition mechanism. *Prep. Biochem. Biotechnol.* **2019**, *49*, 298–306, doi:10.1080/10826068.2018.1541811.
97. Sumantran, V.N.; Kulkarni, A.A.; Harsulkar, A.; Wele, A.; Koppikar, S.J.; Chandwaskar, R.; Gaire, V.; Dalvi, M.; Wagh, U. V Hyaluronidase and collagenase inhibitory activities of the herbal formulation Triphala guggulu. *J. Biosci.* **2007**, *32*, 755–61.
98. Fayad, S.; Nehmé, R.; Tannoury, M.; Lesellier, E.; Pichon, C.; Morin, P. Macroalga Padina pavonica water extracts obtained by pressurized liquid extraction and microwave-assisted extraction inhibit hyaluronidase activity as shown by capillary electrophoresis. *J. Chromatogr. A* **2017**, *1497*, 19–27, doi:10.1016/j.chroma.2017.03.033.
99. Murata, T.; Sasaki, K.; Sato, K.; Yoshizaki, F.; Yamada, H.; Mutoh, H.; Umehara, K.; Miyase, T.; Warashina, T.; Aoshima, H.; et al. Matrix metalloproteinase-2 inhibitors from Clinopodium chinense var. parviflorum. *J. Nat. Prod.* **2009**, *72*, 1379–84, doi:10.1021/np800781t.
100. Nagai, M.; Noguchi, M.; Iizuka, T.; Otani, K.; Kamata, K. Vasodilator effects of des(alpha-carboxy-3,4-dihydroxyphenethyl)lithospermic acid (8-epiblechnic acid), a derivative of lithospermic acids in salviae miltiorrhizae radix. *Biol. Pharm. Bull.* **1996**, *19*, 228–32.
101. P. K. Agrawal and R. P. Rastogi "13C NMR spectroscopy of flavonoids,." *Heterocycles* **1981**, *16*, 2181–2236.
102. Aoshima, H.; Miyase, T.; Warashina, T. Caffeic Acid Oligomers with Hyaluronidase Inhibitory Activity from Clinopodium gracile. *Chem. Pharm. Bull.* **2012**, *60*, 499–507, doi:10.1248/cpb.60.499.
103. Pujiarti, R.; Ohtani, Y. and Ichura, H. Antioxidant, Anti-Hyaluronidase and Antifungal Activities of Melaleuca leucadendron Linn. Leaf Oils. *J. Wood Sci.* **2012**, *58*, 429–436.
104. Moon, S.; Kim, K.; Lee, N.; Han, Y.; Nah, S.; Cho, S.G.; Park, Y.; Paik, H. Inhibitory Effects of Naringenin and Its Novel Derivatives on Hyaluronidase. *Food Sci. Biotechnol.* **2009**, *18*, 267–270.
105. Zeng, H.J.; Yang, R.; You, J.; Qu, L.B.; Sun, Y.J. Spectroscopic and Docking Studies on the Binding of Liquiritigenin with Hyaluronidase for Antiallergic Mechanism. *Scientifica (Cairo).* **2016**, *2016*, doi:10.1155/2016/9178097.
106. Khan, M.T.H. Novel Tyrosinase Inhibitors From Natural Resources - Their Computational Studies. *Curr. Med. Chem.* **2012**, *19*, 2262–2272, doi:CMC-EPUB-20120313-011 [pii].
107. Ryu, H.W.; Song, H.H.; Shin, I.S.; Cho, B.O.; Jeong, S.H.; Kim, D.Y.; Ahn, K.S.; Oh, S.R. Suffruticosol A isolated from Paeonia lactiflora seedcases attenuates airway inflammation in mice induced by cigarette smoke and LPS exposure. *J. Funct. Foods* **2015**, *17*, 774–784, doi:10.1016/j.jff.2015.06.036.
108. Lee, S.Y.; Baek, N.; Nam, T.G. Natural, semisynthetic and synthetic tyrosinase inhibitors. *J. Enzyme Inhib. Med. Chem.* **2016**, *31*, 1–13, doi:10.3109/14756366.2015.1004058.
109. Parvez, S.; Kang, M.; Chung, H.-S.; Bae, H. Naturally occurring tyrosinase inhibitors: mechanism and applications in skin health, cosmetics and agriculture industries. *Phytother. Res.* **2007**, *21*, 805–16, doi:10.1002/ptr.2184.
110. Raper, H.S.; The anaerobic oxidases. *Physiol Rev .* **1928**, *8*, 245– 282.
111. Olivares, C.; Jiménez-Cervantes, C.; Lozano, J.A.; Solano, F.; García-Borrón, J.C. The 5,6-dihydroxyindole-2-carboxylic acid (DHICA) oxidase activity of human tyrosinase. *Biochem. J.* **2001**, *354*, 131–9.
112. Shi, Y.-L.; Benzie, I.F.F.; Buswell, J.A. Role of tyrosinase in the genoprotective effect of the edible mushroom, Agaricus bisporus. *Life Sci.* **2002**, *70*, 1595–608, doi:10.1016/S0024-3205(01)01546-6.
113. Kobayashi, T.; Vieira, W.D.; Potterf, B.; Sakai, C.; Imokawa, G.; Hearing, V.J. Modulation of melanogenic protein expression during the switch from eu- to pheomelanogenesis. *J. Cell Sci.* **1995**, *108 (Pt 6*, 2301–9.
114. Borges, C.R.; Roberts, J.C.; Wilkins, D.G.; Rollins, D.E. Relationship of Melanin Degradation Products to Actual Melanin Content: Application to Human Hair. *Anal. Biochem.* **2001**, *290*, 116–125, doi:10.1006/abio.2000.4976.
115. Miyazawa, M.; Tamura, N. Inhibitory compound of tyrosinase activity from the sprout of Polygonum hydropiper L. (Benitade). *Biol. Pharm. Bull.* **2007**, *30*, 595–7.
116. An, S.M.; Kim, H.J.; Kim, J.-E.; Boo, Y.C. Flavonoids, taxifolin and luteolin attenuate cellular melanogenesis despite increasing tyrosinase protein levels. *Phyther. Res.* **2008**, *22*, 1200–1207, doi:10.1002/ptr.2435.
117. Willför, S.M.; Ahotupa, M.O.; Hemming, J.E.; Reunanen, M.H.T.; Eklund, P.C.; Sjöholm, R.E.; Eckerman, C.S.E.; Pohjamo, S.P.; Holmbom, B.R. Antioxidant activity of knotwood extractives and phenolic compounds of selected tree species. *J. Agric. Food Chem.* **2003**, *51*, 7600–6, doi:10.1021/jf030445h.
118. Tsimogiannis, D.I.; Oreopoulou, V. Free radical scavenging and antioxidant activity of 5,7,3′,4′-hydroxy-substituted flavonoids. *Innov. Food Sci. Emerg. Technol.* **2004**, *5*, 523–528, doi:10.1016/j.ifset.2004.05.006.
119. Zheng, Z.-P.; Zhu, Q.; Fan, C.-L.; Tan, H.-Y.; Wang, M. Phenolic tyrosinase inhibitors from the stems of Cudrania cochinchinensis. *Food Funct.* **2011**, *2*, 259–64, doi:10.1039/c1fo10033e.
120. Zheng, Z.-P.; Cheng, K.-W.; To, J.T.-K.; Li, H.; Wang, M. Isolation of tyrosinase inhibitors from Artocarpus heterophyllus and use of its extract as antibrowning agent. *Mol. Nutr. Food Res.* **2008**, *52*, 1530–8, doi:10.1002/mnfr.200700481.
121. Leu, Y.-L.; Hwang, T.-L.; Hu, J.-W.; Fang, J.-Y. Anthraquinones from Polygonum cuspidatum as tyrosinase inhibitors for dermal use. *Phytother. Res.* **2008**, *22*, 552–6, doi:10.1002/ptr.2324.

122. Kubo, I.; Kinst-Hori, I.; Ishiguro, K.; Chaudhuri, S.K.; Sanchez, Y.; Ogura, T. Tyrosinase inhibitory flavonoids from heterotheca inuloides and their structural functions. *Bioorganic Med. Chem. Lett.* **1994**, *4*, 1443–1446, doi:10.1016/S0960-894X(01)80510-2.
123. Kubo, I.; Kinst-Hori, I. Flavonols from saffron flower: tyrosinase inhibitory activity and inhibition mechanism. *J. Agric. Food Chem.* **1999**, *47*, 4121–5.
124. Wang, H.-M.; Chen, C.-Y.; Chen, C.-Y.; Ho, M.-L.; Chou, Y.-T.; Chang, H.-C.; Lee, C.-H.; Wang, C.-Z.; Chu, I.-M. (−)-N-Formylanonaine from Michelia alba as a human tyrosinase inhibitor and antioxidant. *Bioorg. Med. Chem.* **2010**, *18*, 5241–5247, doi:10.1016/j.bmc.2010.05.045.
125. Wu, B.; Zhang, X.; Wu, X. New lignan glucosides with tyrosinase inhibitory activities from exocarp of Castanea henryi. *Carbohydr. Res.* **2012**, *355*, 45–49, doi:10.1016/j.carres.2012.04.009.
126. Akihisa, T.; Orido, M.; Akazawa, H. et al. Melanogenesis-inhibitory activity of aromatic glycosides from the stem bark of Acer buergerianum. *Chem Biodivers* **2013**, *10*, 167–175.
127. Hashim, N.M.; Rahmani, M.; Ee, G.C.L.; Sukari, M.A.; Yahayu, M.; Amin, M.A.M.; Ali, A.M.; Go, R. Antioxidant, Antimicrobial and Tyrosinase Inhibitory Activities of Xanthones Isolated from Artocarpus obtusus F.M. Jarrett. *Molecules* **2012**, *17*, 6071–6082, doi:10.3390/molecules17056071.
128. Chen, W.-C.; Tseng, T.-S.; Hsiao, N.-W.; Lin, Y.-L.; Wen, Z.-H.; Tsai, C.-C.; Lee, Y.-C.; Lin, H.-H.; Tsai, K.-C. Discovery of highly potent tyrosinase inhibitor, T1, with significant anti-melanogenesis ability by zebrafish in vivo assay and computational molecular modeling. *Sci. Rep.* **2015**, *5*, 7995, doi:10.1038/srep07995.
129. Pillaiyar, T.; Manickam, M.; Namasivayam, V. Skin whitening agents: medicinal chemistry perspective of tyrosinase inhibitors. *J. Enzyme Inhib. Med. Chem.* **2017**, *32*, 403–425, doi:10.1080/14756366.2016.1256882.
130. Limtrakul, P.; Yodkeeree, S.; Thippraphan, P.; Punfa, W.; Srisomboon, J. Anti-aging and tyrosinase inhibition effects of Cassia fistula flower butanolic extract. *BMC Complement. Altern. Med.* **2016**, *16*, 497, doi:10.1186/s12906-016-1484-3.
131. Luximon-Ramma, A.; Bahorun, T.; Soobrattee, M.A.; Aruoma, O.I. Antioxidant activities of phenolic, proanthocyanidin, and flavonoid components in extracts of Cassia fistula. *J. Agric. Food Chem.* **2002**, *50*, 5042–7.
132. Manonmani, G.; Bhavapriya, V.; Kalpana, S.; Govindasamy, S.; Apparanantham, T. Antioxidant activity of Cassia fistula (Linn.) flowers in alloxan induced diabetic rats. *J. Ethnopharmacol.* **2005**, *97*, 39–42, doi:10.1016/j.jep.2004.09.051.
133. Bhalodia, N.R.; Shukla, V.J. Antibacterial and antifungal activities from leaf extracts of Cassia fistula l.: An ethnomedicinal plant. *J. Adv. Pharm. Technol. Res.* **2011**, *2*, 104–9, doi:10.4103/2231-4040.82956.
134. Dai, Y.; Zhou, G.; Kurihara, H.; Ye, W.; Yao, X. Biphenyl Glycosides from the Fruit of *Pyracantha f ortuneana*. *J. Nat. Prod.* **2006**, *69*, 1022–1024, doi:10.1021/np0600853.
135. Wang C.; Jia, Z. Lignan, phenylpropanoid and iridoid glycosides from Pedicularis torta. *Phytochemistry* **1997**, *45*, 159–166.
136. Huang, X.X.; Liu, Q.B.; Wu, J.; Yu, L.H.; Cong, Q.; Zhang, Y.; Lou, L.L.; Li, L.Z.; Song, S.J. Antioxidant and Tyrosinase Inhibitory Effects of Neolignan Glycosides from Crataegus pinnatifida Seeds. *Planta Med.* **2014**, *80*, 1732–1738, doi:10.1055/s-0034-1383253.
137. Cömert Önder, F.; Ay, M.; Aydoğan Türkoğlu, S.; Tura Köçkar, F.; Çelik, A. Antiproliferative activity of *Humulus lupulus* extracts on human hepatoma (Hep3B), colon (HT-29) cancer cells and proteases, tyrosinase, β -lactamase enzyme inhibition studies. *J. Enzyme Inhib. Med. Chem.* **2016**, *31*, 90–98, doi:10.3109/14756366.2015.1004060.
138. Arung, E.T.; Shimizu, K.; Kondo, R. Structure-activity relationship of prenyl-substituted polyphenols from Artocarpus heterophyllus as inhibitors of melanin biosynthesis in cultured melanoma cells. *Chem. Biodivers.* **2007**, *4*, 2166–71, doi:10.1002/cbdv.200790173.
139. Jin, Y.J.; Lin, C.C.; Lu, T.M.; Li, J.H.; Chen, I.S.; Kuo, Y.H.; Ko, H.H. Chemical constituents derived from Artocarpus xanthocarpus as inhibitors of melanin biosynthesis. *Phytochemistry* **2015**, *117*, 424–435, doi:10.1016/j.phytochem.2015.07.003.
140. Ko, H.-H.; Jin, Y.-J.; Lu, T.-M.; Chen, I.-S. A novel monoterpene-stilbene adduct with a 4,4-dimethyl-2,3-diphenylchromane skeleton from Artocarpus xanthocarpus. *Chem. Biodivers.* **2013**, *10*, 1269–75, doi:10.1002/cbdv.201200377.
141. Lin, Y.P.; Hsu, F.L.; Chen, C.S.; Chern, J.W.; Lee, M.H. Constituents from the Formosan apple reduce tyrosinase activity in human epidermal melanocytes. *Phytochemistry* **2007**, *68*, 1189–1199, doi:10.1016/j.phytochem.2007.02.001.
142. Lee, M.G.; Kuo, S.Y.; Yen, S.Y.; Hsu, H.F.; Leung, C.H.; Ma, D.L.; Wen, Z.H.; Wang, H.M.D. Evaluation of cinnamomum osmophloeum kanehira extracts on tyrosinase suppressor, wound repair promoter, and antioxidant. *Sci. World J.* **2015**, *2015*, doi:10.1155/2015/303415.
143. Wang, Z.; Hwang, S.H.; Huang, B.; Lim, S.S. Identification of tyrosinase specific inhibitors from Xanthium strumarium fruit extract using ultrafiltration-high performance liquid chromatography. *J. Chromatogr. B Anal. Technol. Biomed. Life Sci.* **2015**, *1002*, 319–328, doi:10.1016/j.jchromb.2015.08.030.
144. Bajpai, V.K.; Park, Y.-H.; Na, M.; Kang, S.C. α-Glucosidase and tyrosinase inhibitory effects of an abietane type diterpenoid taxoquinone from Metasequoia glyptostroboides. *BMC Complement. Altern. Med.* **2015**, *15*, 84, doi:10.1186/s12906-015-0626-3.
145. Batubara, I.; Kuspradini, H.; Mitsunaga, T. Anti-acne and Tyrosinase Inhibition Properties of Taxifolin and Some Flavanonol Rhamnosides 45 from Kempas (Koompassia malaccensis) Anti-acne and Tyrosinase Inhibition Properties of Taxifolin and Some Flavanonol Rhamnosides from Kempas (Koompassia malaccensis).
146. Jones, K.; Hughes, J.; Hong, M.; Jia, Q.; Orndorff, S. Modulation of melanogenesis by aloesin: A competitive inhibitor of tyrosinase. *Pigment Cell Res.* **2002**, *15*, 335–340, doi:10.1034/j.1600-0749.2002.02014.x.

147. Yagi, A.; Kanbara, T.; Morinobu, N. Inhibition of mushroom-tyrosinase by aloe extract. *Planta Med.* **1987**, *53*, 515–7, doi:10.1055/s-2006-962798.
148. Jin, Y.H.; Lee, S.J.; Chung, M.H.; Park, J.H.; Park, Y.I.; Cho, T.H.; Lee, S.K. Aloesin and arbutin inhibit tyrosinase activity in a synergistic manner via a different action mechanism. *Arch. Pharm. Res.* **1999**, *22*, 232–6.
149. Choi, S.; Lee, S.-K.; Kim, J.-E.; Chung, M.-H.; Park, Y.-I. Aloesin inhibits hyperpigmentation induced by UV radiation. *Clin. Exp. Dermatol.* **2002**, *27*, 513–5.
150. Wu, B.; Chen, J.; Qu, H.; Cheng, Y. Complex sesquiterpenoids with tyrosinase inhibitory activity from the leaves of Chloranthus tianmushanensis. *J. Nat. Prod.* **2008**, *71*, 877–80, doi:10.1021/np070623r.
151. Kubo, I.; Kinst-Hori, I.; Chaudhuri, S.K.; Kubo, Y.; Sánchez, Y.; Ogura, T. Flavonols from Heterotheca inuloides: Tyrosinase inhibitory activity and structural criteria. *Bioorganic Med. Chem.* **2000**, *8*, 1749–1755, doi:10.1016/S0968-0896(00)00102-4.
152. Kubo, I.; Yokokawa, Y. Two tyrosinase inhibiting flavonol glycosides from Buddleia coriacea. *Phytochemistry* **1992**, *31*, 1075–1077, doi:10.1016/0031-9422(92)80084-R.
153. Biswas, R.; Chanda, J.; Kar, A.; Mukherjee, P.K. Tyrosinase inhibitory mechanism of betulinic acid from Dillenia indica. *Food Chem.* **2017**, *232*, 689–696, doi:10.1016/j.foodchem.2017.04.008.
154. Muñoz, E.; Avila, J.G.; Alarcón, J.; Kubo, I.; Werner, E.; Céspedes, C.L. Tyrosinase inhibitors from calceolaria integrifolia s.l.: Calceolaria talcana aerial parts. *J. Agric. Food Chem.* **2013**, *61*, 4336–4343, doi:10.1021/jf400531h.
155. Biswas, R.; Mukherjee, P.K.; Chaudhary, S.K. Tyrosinase inhibition kinetic studies of standardized extract of Berberis aristata. *Nat. Prod. Res.* **2016**, *30*, 1451–1454, doi:10.1080/14786419.2015.1062376.
156. Lee, C.C.; Chen, Y.T.; Chiu, C.C.; Liao, W.T.; Liu, Y.C.; David Wang, H.M. Polygonum cuspidatum extracts as bioactive antioxidaion, anti-tyrosinase, immune stimulation and anticancer agents. *J. Biosci. Bioeng.* **2015**, *119*, 464–469, doi:10.1016/j.jbiosc.2014.09.008.
157. Lee, M. H.; Kao, L.; and Lin, C.C. Comparison of the antioxidant and transmembrane permeative activities of the different Polygonum cuspidatum extracts in phospholipid-based microemulsions. *J. Agric. Food Chem.* **2011**, *59*, 9135–9141.
158. Zhang, P.C.; Feng Z.M., and W.Y. Flavonoids, Including an Unusual Flavonoids-Mg2+ Salt, from Roots of Cudrania cochinchinensis. *Phytochemistry* **2005**, *66*, 2759–2765.
159. Nguyen, N.T.; Nguyen, M.H.K.; Nguyen, H.X.; Bui, N.K.N.; Nguyen, M.T.T. Tyrosinase inhibitors from the wood of Artocarpus heterophyllus. *J. Nat. Prod.* **2012**, *75*, 1951–5, doi:10.1021/np300576w.
160. Arung, E.T.; Yoshikawa, K.; Shimizu, K.; Kondo, R. Isoprenoid-substituted flavonoids from wood of Artocarpus heterophyllus on B16 melanoma cells: cytotoxicity and structural criteria. *Fitoterapia* **2010**, *81*, 120–3, doi:10.1016/j.fitote.2009.08.001.
161. Tan, X.; Song, Y.H.; Park, C.; Lee, K.W.; Kim, J.Y.; Kim, D.W.; Kim, K.D.; Lee, K.W.; Curtis-Long, M.J.; Park, K.H. Highly potent tyrosinase inhibitor, neorauflavane from Campylotropis hirtella and inhibitory mechanism with molecular docking. *Bioorganic Med. Chem.* **2016**, *24*, 153–159, doi:10.1016/j.bmc.2015.11.040.
162. Zolghadri, S.; Bahrami, A.; Hassan Khan, M.T.; Munoz-Munoz, J.; Garcia-Molina, F.; Garcia-Canovas, F.; Saboury, A.A. A comprehensive review on tyrosinase inhibitors. *J. Enzyme Inhib. Med. Chem.* **2019**, *34*, 279–309, doi:10.1080/14756366.2018.1545767.
163. Wang, Y.; Curtis-Long, M.J.; Lee, B.W.; Yuk, H.J.; Kim, D.W.; Tan, X.F.; Park, K.H. Inhibition of tyrosinase activity by polyphenol compounds from Flemingia philippinensis roots. *Bioorganic Med. Chem.* **2014**, *22*, 1115–1120, doi:10.1016/j.bmc.2013.12.047.
164. Hubert, J.; Angelis, A.; Aligiannis, N.; Rosalia, M.; Abedini, A.; Bakiri, A.; Reynaud, R.; Nuzillard, J.M.; Gangloff, S.C.; Skaltsounis, A.L.; et al. In vitro dermo-cosmetic evaluation of bark extracts from common temperate trees. *Planta Med.* **2016**, *82*, 1351–1358, doi:10.1055/s-0042-110180.
165. Lin, Q.M.; Wang, Y.; Yu, J.H.; Liu, Y.L.; Wu, X.; He, X.R.; Zhou, Z.W. Tyrosinase inhibitors from the leaves of Eucalyptus globulus. *Fitoterapia* **2019**, *139*, 104418, doi:10.1016/j.fitote.2019.104418.
166. Shi, F.; Xie, L.; Lin, Q.; Tong, C.; Fu, Q.; Xu, J.; Xiao, J.; Shi, S. Profiling of tyrosinase inhibitors in mango leaves for a sustainable agro-industry. *Food Chem.* **2020**, *312*, 126042, doi:10.1016/j.foodchem.2019.126042.
167. Yang, Y.; Sun, X.; Ni, H.; Du, X.; Chen, F.; Jiang, Z.; Li, Q. Identification and Characterization of the Tyrosinase Inhibitory Activity of Caffeine from Camellia Pollen; 2019; Vol. 67; ISBN 8618205990.
168. Castañeda-Loaiza, V.; Placines, C.; Rodrigues, M.J.; Pereira, C.G.; Zengin, G.; Neng, N.R.; Nogueira, J.M.F.; Custódio, L. In vitro enzyme inhibitory and anti-oxidant properties, cytotoxicity and chemical composition of the halophyte Malcolmia littorea (L.) R.Br. (Brassicaceae). *Nat. Prod. Res.* **2020**, *0*, 1–4, doi:10.1080/14786419.2020.1719484.
169. Sinan, K.I.; Llorent-Martínez, E.J.; Bene, K.; Mahomoodally, M.F.; Lobine, D.; Aktumsek, A.; Zengin, G. Novel insights into the fruit and seed extracts of Morinda morindoides (Baker) Milne-Redh: HPLC-ESI-Q-TOF-MS profiling, antioxidant, and enzyme inhibitory propensities. *J. Food Biochem.* **2020**, *44*, 1–9, doi:10.1111/jfbc.13169.
170. Cimanga, R., Mukenyi, P., Kambu, O., Tona, G., Apers, S., Totte, J., Vlietinck, A. The spasmolytic activity of extracts and some isolated compounds from the leaves of Morinda morindoides (Baker) Milne-Redh. (Rubiaceae). *J. Ethnopharmacol.* **2010**, *127*, 215–220.
171. Marie-Genevieve, O., Robin, O. P., Gregory, G., Catherine, L., & C.; M. Cytotoxic effect induced by Morinda morindoides leaf extracts in human and murine leukemia cells. *African J. Biotechnol.* **2010**, *9*, 6560–6565.

172. Tona, L., Mesia, K., Ngimbi, N., Chrimwami, B., Okond'Ahoka, C.; K., Totte, J. In-vivo antimalarial activity of Cassia occidentalism, Morinda morindoides and Phyllanthus niruri. *Ann. Trop. Med. Parasitol.* **2001**, *95*, 47–57.
173. Placines, C.; Castañeda-Loaiza, V.; Rodrigues, M.J.; Pereira, C.G.; Stefanucci, A.; Mollica, A.; Zengin, G.; Llorent-Martínez, E.J.; Castilho, P.C.; Custódio, L. Phenolic profile, toxicity, enzyme inhibition, in silico studies, and antioxidant properties of cakile maritima scop. (brassicaceae) from Southern Portugal. *Plants* **2020**, *9*, 1–24, doi:10.3390/plants9020142.
174. Davy, A.J.; Scott, R.; Cordazzo, C.V. Biological flora of the British Isles: Cakile maritima Scop. *J. Ecol.* **2006**, *94*, 695–71.
175. Fuochi, V.; Barbagallo, I.; Distefano, A.; Puglisi, F.; Palmeri, R.; Rosa, M.D.I.; Giallongo, C.; Longhitano, L.; Fontana, P.; Sferrazzo, G.. et al. Biological properties of Cakile maritima Scop. (Brassicaceae) extracts. *Eur. Rev. Med Pharmacol. Sci.* **2019**, *23*, 2280–2292.
176. Kim, J.H.; Leem, H.H.; Lee, G.Y. The guanidine pseudoalkaloids 10-methoxy- leonurine and leonurine act as competitive inhibitors of tyrosinase. *Biomolecules* **2020**, *10*, doi:10.3390/biom10020174.
177. Zhong, W.-M.; Cui, Z.-M.; Liu, Z.-K.; Yang, Y.; Wu, D.-R.; Liu, S.-H.; Long, H.; Sun, H.-D.; Dang, Y. -J. . X.; W.-L. Three minor new compounds from the aerial parts of Leonurus japonicas. *Chinese Chem. Lett.* **2015**, *26*, 1000–1003.
178. Honisch, C.; Osto, A.; Dupas de Matos, A.; Vincenzi, S.; Ruzza, P. Isolation of a tyrosinase inhibitor from unripe grapes juice: A spectrophotometric study. *Food Chem.* **2020**, *305*, 125506, doi:10.1016/j.foodchem.2019.125506.
179. Mutschlechner, B.; Rainer, B.; Schwaiger, S.; Stuppner, H. Tyrosinase Inhibitors from the Aerial Parts of Wulfenia carinthiaca Jacq. *Chem. Biodivers.* **2018**, *15*, 4–11, doi:10.1002/cbdv.201800014.
180. Ishihara, A.; Ide, Y.; Bito, T.; Ube, N.; Endo, N.; Sotome, K.; Maekawa, N.; Ueno, K.; Nakagiri, A. Novel tyrosinase inhibitors from liquid culture of Neolentinus lepideus. *Biosci. Biotechnol. Biochem.* **2018**, *82*, 22–30, doi:10.1080/09168451.2017.1415125.
181. Revoltella, S.; Rainer, B.; Waltenberger, B.; Pagitz, K.; Schwaiger, S.; Stuppner, H. HPTLC Autography Based Screening and Isolation of Mushroom Tyrosinase Inhibitors of European Plant Species. *Chem. Biodivers.* **2019**, *16*, doi:10.1002/cbdv.201800541.
182. Han, E.B.; Chang, B.Y.; Kim, D.S.; Cho, H.K.; Kim, S.Y. Melanogenesis inhibitory effect of aerial part of Pueraria thunbergiana in vitro and in vivo. *Arch. Dermatol. Res.* **2014**, *307*, 57–72, doi:10.1007/s00403-014-1489-z.
183. Qu, L.; Song, K.; Zhang, Q.; Guo, J.; Huang, J. Simultaneous determination of six isoflavones from puerariae lobatae radix by CPE-HPLC and effect of puerarin on tyrosinase activity. *Molecules* **2020**, *25*, doi:10.3390/molecules25020344.
184. Lim DW, Lee C, Kim IH, K.Y. Anti-inflammatory effects of total isoflavones from Pueraria lobata on cerebral ischemia in rats. *Molecules* **2013**, *18*, 10404–10412.
185. Xiong Y, Yang Y, Yang J, Chai H, Li Y, Jia Z, W.Z. Tectoridin, an isoflavone glycoside from the flower of Pueraria lobata, prevents acute ethanol-induced liver steatosis in mice. *Toxicology* **2010**, *276*, 64–72.
186. Chai, W.M.; Wei, Q.M.; Deng, W.L.; Zheng, Y.L.; Chen, X.Y.; Huang, Q.; Ou-Yang, C.; Peng, Y.Y. Anti-melanogenesis properties of condensed tannins from: Vigna angularis seeds with potent antioxidant and DNA damage protection activities. *Food Funct.* **2019**, *10*, 99–111, doi:10.1039/c8fo01979g.
187. Wolfender, J.-L.; Marti, G.; Thomas, A.; Bertrand, S. Current approaches and challenges for the metabolite profiling of complex natural extracts. *J. Chromatogr. A* **2015**, *1382*, 136–164, doi:10.1016/j.chroma.2014.10.091.
188. Brewer, M.S. Natural Antioxidants: Sources, Compounds, Mechanisms of Action, and Potential Applications. *Compr. Rev. Food Sci. Food Saf.* **2011**, *10*, 221–247, doi:10.1111/j.1541-4337.2011.00156.x.
189. Zheng, Z.-P.P.; Tan, H.-Y.Y.; Chen, J.; Wang, M. Characterization of tyrosinase inhibitors in the twigs of Cudrania tricuspidata and their structure-activity relationship study. *Fitoterapia* **2013**, *84*, 242–247, doi:10.1016/j.fitote.2012.12.006.
190. Hano, Y.; Matsumoto, Y.; Shinohara, K., Sun, J.Y.; Nomura, T. Cudraflavones C and D, two new prenylflavones from the root bark of Cudrania tricuspidata (Carr.). *Bur. Heterocycles* **1990**, *31*, 1339–1341.
191. Zheng, Z.-P.; Liang, J.-Y.; Hu, L.-H. Water-Soluble Constituents of Cudrania tricuspidata (Carr.) Bur. *J. Integr. Plant Biol.* **2006**, *48*, 996–1000, doi:10.1111/j.1744-7909.2006.00227.x.
192. No, J.K.; Soung, D.Y.; Kim, Y.J.; Shim, K.H.; Jun, Y.S.; Rhee, S.H.; Yokozawa, T.; Chung, H.Y. Inhibition of tyrosinase by green tea components. *Life Sci.* **1999**, *65*, PL241–PL246, doi:10.1016/S0024-3205(99)00492-0.
193. Chang, T.-S. An Updated Review of Tyrosinase Inhibitors. *Int. J. Mol. Sci.* **2009**, *10*, 2440–2475, doi:10.3390/ijms10062440.
194. Chen, Q.-X.; Kubo, I. Kinetics of Mushroom Tyrosinase Inhibition by Quercetin. *J. Agric. Food Chem.* **2002**, *50*, 4108–4112, doi:10.1021/jf011378z.
195. Xie, L.-P.; Chen, Q.-X.; Huang, H.; Wang, H.-Z.; Zhang, R.-Q. Inhibitory effects of some flavonoids on the activity of mushroom tyrosinase. *Biochemistry. (Mosc).* **2003**, *68*, 487–91.
196. Kim, Y.-J.; Uyama, H. Tyrosinase inhibitors from natural and synthetic sources: structure, inhibition mechanism and perspective for the future. *Cell. Mol. Life Sci.* **2005**, *62*, 1707–1723, doi:10.1007/s00018-005-5054-y.
197. Kim, Y.J. Rhamnetin attenuates melanogenesis by suppressing oxidative stress and pro-inflammatory mediators. *Biol. Pharm. Bull.* **2013**, *36*, 1341–7, doi:10.1248/bpb.b13-00276.
198. Seo, S.Y.; Sharma, V.K.; Sharma, N. Mushroom tyrosinase: Recent prospects. *J. Agric. Food Chem.* **2003**, *51*, 2837–2853, doi:10.1021/jf020826f.

199. Wu, Y.; Wu, Z.R.; Chen, P.; Yang-Li; Deng, W.R.; Wang, Y.Q.; Li, H.Y. Effect of the tyrosinase inhibitor (S)-N-trans-feruloyloctopamine from garlic skin on tyrosinase gene expression and melanine accumulation in melanoma cells. *Bioorganic Med. Chem. Lett.* **2015**, *25*, 1476–1478, doi:10.1016/j.bmcl.2015.02.028.
200. Yi Dai, †; Guang-xiong Zhou, ‡; Hiroshi Kurihara, ‡; Wen-cai Ye, ‡ and; Xin-sheng Yao*, †,‡ Biphenyl Glycosides from the Fruit of Pyracantha fortuneana. **2006**, doi:10.1021/NP0600853.
201. Ebanks, J.P.; Wickett, R.R.; Boissy, R.E. Mechanisms regulating skin pigmentation: The rise and fall of complexion coloration. *Int. J. Mol. Sci.* **2009**, *10*, 4066–4087, doi:10.3390/ijms10094066.
202. Draelos, Z.D. Skin lightening preparations and the hydroquinone controversy. *Dermatol. Ther.* **2007**, *20*, 308–13, doi:10.1111/j.1529-8019.2007.00144.x.
203. Kim, S.J.; Son, K.H.; Chang, H.W.; Kang, S.S.; Kim, H.P. Tyrosinase inhibitory prenylated flavonoids from Sophora flavescens. *Biol. Pharm. Bull.* **2003**, *26*, 1348–50.
204. Hyun, S.K.; Lee, W.-H.; Jeong, D.M.; Kim, Y.; Choi, J.S. Inhibitory effects of kurarinol, kuraridinol, and trifolirhizin from Sophora flavescens on tyrosinase and melanin synthesis. *Biol. Pharm. Bull.* **2008**, *31*, 154–8.
205. Shin, N.H.; Ryu, S.Y.; Choi, E.J.; Kang, S.H.; Chang, I.M.; Min, K.R.; Kim, Y. Oxyresveratrol as the potent inhibitor on dopa oxidase activity of mushroom tyrosinase. *Biochem. Biophys. Res. Commun.* **1998**, *243*, 801–3, doi:10.1006/bbrc.1998.8169.
206. Cruz-Vega, D.; Verde-Star, M.J.; Salinas-Gonzalez, N.R.; Rosales-Hernandez, B.; Estrada-Garcia, I.; Mendez-Aragon, P.; Carranza-Rosales, P.; Gonzalez-Garza, M.; Castro-Garza, J. Review of pharmacological effects of Glycyrrhiza radix and its bioactive compounds. *Zhongguo Zhong Yao Za Zhi* **2009**, *22*, 557–559, doi:10.1002/ptr.
207. Kubo, I.; Kinst-Hori, I. 2-Hydroxy-4-methoxybenzaldehyde: a potent tyrosinase inhibitor from African medicinal plants. *Planta Med.* **1999**, *65*, 19–22.
208. Ha, T.J.; Tamura, S.; Kubo, I. Effects of mushroom tyrosinase on anisaldehyde. *J. Agric. Food Chem.* **2005**, *53*, 7024–8, doi:10.1021/jf047943q.
209. Lee, H.-S. Tyrosinase inhibitors of Pulsatilla cernua root-derived materials. *J. Agric. Food Chem.* **2002**, *50*, 1400–3.
210. Isao Kubo, and I.K.-H. Tyrosinase Inhibitors from Cumin. **1998**, doi:10.1021/JF980226+.
211. Jiménez, M.; Chazarra, S.; Escribano, J.; Cabanes, J.; García-Carmona, F. Competitive inhibition of mushroom tyrosinase by 4-substituted benzaldehydes. *J. Agric. Food Chem.* **2001**, *49*, 4060–3.
212. D. Datta and S. Kumar Modeling using response surface methodology and optimization using differential evolution of reactive extraction of glycolic acid,. *Chem Eng Commun, 202,* 59–69.
213. Ma, D.; Tu, Z.C.; Wang, H.; Zhang, L.; He, N.; McClements, D.J. Mechanism and kinetics of tyrosinase inhibition by glycolic acid: A study using conventional spectroscopy methods and hydrogen/deuterium exchange coupling with mass spectrometry. *Food Funct.* **2017**, *8*, 122–131, doi:10.1039/c6fo01384h.
